# Brain contrastive modeling reveals depression subtypes with distinct treatment response and progression

**DOI:** 10.1038/s41746-026-03011-8

**Published:** 2026-07-20

**Authors:** Kaizhong Zheng, Xinhu Zheng, Hongyu Shi, Liangjun Chen, Lingjiang Li, DIRECT consortium, Li-Ping Cao, Guan-Mao Chen, Jian-Shan Chen, Tao Chen, Tao-Lin Chen, Yu-Qi Cheng, Zhao-Song Chu, Shi-Xian Cui, Xi-Long Cui, Zhao-Yu Deng, Qi-Yong Gong, Wen-Bin Guo, Can-Can He, Zheng-Jia-Yi Hu, Qian Huang, Xin-Lei Ji, Feng-Nan Jia, Li Kuang, Bao-Juan Li, Feng Li, Hui-Xian Li, Tao Li, Tao Lian, Yi-Fan Liao, Xiao-Yun Liu, Yan-Song Liu, Zhe-Ning Liu, Yi-Cheng Long, Jian-Ping Lu, Jiang Qiu, Xiao-Xiao Shan, Tian-Mei Si, Peng-Feng Sun, Chuan-Yue Wang, Hua-Ning Wang, Xiang Wang, Ying Wang, Yu-Wei Wang, Xiao-Ping Wu, Xin-Ran Wu, Yan-Kun Wu, Chun-Ming Xie, Guang-Rong Xie, Peng Xie, Xiu-Feng Xu, Zhen-Peng Xue, Hong Yang, Hua Yu, Min-Lan Yuan, Yong-Gui Yuan, Ai-Xia Zhang, Jing-Ping Zhao, Ke-Rang Zhang, Wei Zhang, Zi-Jing Zhang, Chao-Gan Yan, Dewen Hu, Karl J. Friston, Huaning Wang, Baojuan Li, Badong Chen

**Affiliations:** 1 National Key Laboratory of Human-Machine Hybrid Augmented Intelligence, National Engineering Research Center for Visual Information and Applications, and Institute of Artificial Intelligence and Robotics, Xi’an Jiaotong University, Xi’an, China; 2 Intelligent Transportation Thrust, The Hong Kong University of Science and Technology (Guangzhou), Guangzhou, China; 3 Department of Psychiatry, The Second Xiangya Hospital, Central South University, Changsha, China; 4 Department of Intelligence Science and Technology, College of Intelligence Science and Technology, National University of Defense Technology, Changsha, China; 5 The Wellcome Department of Imaging Neuroscience, Institute of Neurology, University College London, London, UK; 6 Xijing Hospital, Fourth Military Medical University, Xi’an, China; 7 School of Biomedical Engineering, Fourth Military Medical University, Xi’an, China; 8 Affiliated Brain Hospital of Guangzhou Medical University, Guangzhou, China; 9 The First Affiliated Hospital of Jinan University, Guangzhou, China; 10 Department of Radiology, The First Affiliated Hospital, College of Medicine, Zhejiang University, Hangzhou, China; 11 Huaxi Magnetic Resonance Research Center, Department of Radiology, West China Hospital of Sichuan University, Chengdu, China; 12 Research Unit of Psychoradiology, Chinese Academy of Medical Sciences, Chengdu, China; 13 Department of Psychiatry, First Affiliated Hospital of Kunming Medical University, Kunming, China; 14 Chinese Academy of Sciences Key Laboratory of Behavioral Science, Institute of Psychology, Chinese Academy of Sciences, Beijing, China; 15 Sino-Danish College, University of Chinese Academy of Sciences, Beijing, China; 16 Sino-Danish Center for Education and Research, Graduate University of Chinese Academy of Sciences, Beijing, China; 17 Department of Psychiatry and National Clinical Research Center for Mental Disorders, The Second Xiangya Hospital of Central South University, Changsha, China; 18 Department of Neurology, Affiliated ZhongDa Hospital of Southeast University, Nanjing, China; 19 Department of Psychiatry, The First Affiliated Hospital of Chongqing Medical University, Chongqing, China; 20 Department of Clinical Psychology, Suzhou Psychiatric Hospital, The Affiliated Guangji Hospital of Soochow University, Suzhou, China; 21 Beijing Anding Hospital, Capital Medical University, Beijing, China; 22 Affiliated Mental Health Center and Hangzhou Seventh People’s Hospital, Zhejiang University School of Medicine, Hangzhou, China; 23 Mental Health Center and Psychiatric Laboratory, West China Hospital of Sichuan University, Chengdu, China; 24 Department of Psychosomatics and Psychiatry, Zhongda Hospital, School of Medicine, Southeast University, Nanjing, China; 25 Shenzhen Kangning Hospital, Shenzhen, China; 26 Faculty of Psychology, Southwest University, Chongqing, China; 27 National Clinical Research Center for Mental Disorders (Peking University Sixth Hospital) and Key Laboratory of Mental Health, Ministry of Health (Peking University), Beijing, China; 28 Xi’an Central Hospital, Xi’an, China; 29 Institute of Neuroscience, Chongqing Medical University, Chongqing, China; 30 Chongqing Key Laboratory of Neurobiology, Chongqing, China; 31 Department of Neurology, The First Affiliated Hospital of Chongqing Medical University, Chongqing, China; 32 West China Hospital of Sichuan University, Chengdu, China; 33 First Hospital of Shanxi Medical University, Taiyuan, China

**Keywords:** Computational biology and bioinformatics, Diseases, Medical research, Neurology, Neuroscience

## Abstract

Major Depressive Disorder (MDD) is highly heterogeneous, limiting treatment efficacy. Despite efforts to delineate patient heterogeneity through subtyping, current approaches remain limited by noise, lack of clinical applicability, and insufficient external validation. Crucially, they focus on subtyping while neglecting staging information (e.g., illness duration). We developed BrainCVAE, a contrastive variational autoencoder, to disentangle MDD-specific neural features. Applying BrainCVAE to fALFF-derived resting-state fMRI from 1590 patients and 1308 controls identified two subtypes: Subtype 1 with hyperactivity in visual, attention, and default mode networks, and Subtype 2 with hypoactivity. Subtypes were validated in 1276 patients across independent centers. Subtype 1 showed superior responsiveness to pharmacological (SSRIs, SNRIs) and non-pharmacological (rTMS) interventions. Cross-sectional analyses revealed subtype-specific differences in DMN profiles across illness duration: Subtype 1 shifted from hyper- to hypoactivity, whereas Subtype 2 remained consistently hypoactive. In an independent dataset, illness duration correlated negatively with symptom reduction (*r* = −0.5565, 95% CI = (−0.8123, −0.1210), *p* = 0.0165). Datasets were ethically approved and registered on ClinicalTrials.gov: XJ_QG (NCT05577481, May 24, 2023), SAINT (NCT04653337, Oct 21, 2020), XJ_KG (NCT05544071, May 24, 2023). Integrating subtyping with illness staging bridges neurobiological heterogeneity and disease progression, providing a clinically actionable framework for precision treatment in MDD.

## Introduction

Major depressive disorder (MDD) is a severe mental illness that affects more than 320 million people worldwide, posing a significant global health and economic burden^1,2^. As the leading cause of disability globally, MDD has significant impacts on daily life, work, and relationships^3–5^. MDD is a highly heterogeneous clinical syndrome^1^. The pronounced heterogeneity substantially limits treatment efficacy, as evidenced by the persistently low initial remission rates, which remain around 30%^6^. Furthermore, the efficacy of antidepressant medications compared to placebo is modest, with Cohen’s *d* estimated at approximately 0.3, highlighting the limited therapeutic benefit^7–9^. This clinical heterogeneity poses a major challenge for disease diagnosis and treatment prediction, yet its underlying neural mechanisms remain largely unknown.

To address this challenge, researchers have pursued precise subtyping approaches to delineate the underlying heterogeneity, with the goal of improving treatment efficacy. Symptom-based classification schemes have identified four distinct subtypes of MDD^10,11^. However, clinical symptoms exhibit complex interactions with underlying biological substrates and may fluctuate with age and disease trajectory. In recent years, the Research Domain Criteria framework was introduced, redefining MDD as a disorder of brain circuits and advocating for biologically informed subtyping beyond symptom-based classifications^12^. Leveraging objective neuroimaging modalities such as structural magnetic resonance imaging, functional connectivity (FC) measures, and fractional anisotropy (FA) measures has enabled deeper investigation into depression subtypes^13–16^, as summarized in Supplementary Table 1. Most of these studies have focused primarily on structural or resting-state features. More recently, Tozzi et al. incorporated both task-free and task-evoked fMRI to identify six biotypes in depression and anxiety, which differed in network connectivity, symptom severity, cognitive performance, and treatment response^15^. These data-driven subtyping analyses have identified homogeneous subgroups of MDD patients who exhibit unique psychopathology and demonstrate similar patterns of brain dysfunction^17^. These efforts aim to decode the biological mechanisms underlying this heterogeneity, laying the foundation for more targeted and effective therapeutic interventions.

However, several technical challenges remain to be addressed. A primary challenge is to ensure that the identified subtypes accurately capture the intrinsic biological features of MDD, while mitigating the influence of confounding factors, such as genetic and environmental effects^18,19^. Furthermore, despite growing interest in linking MDD subtypes to treatment outcomes^16,20–22^, the association remains poorly delineated, with the underlying neural mechanisms of response heterogeneity still elusive. For example, hierarchical clustering has revealed MDD subtypes associated with responses to rTMS^17^, while heterogeneity through discriminative analysis (HYDRA) has identified subtypes that differentially respond to SSRI (Selective Serotonin Reuptake Inhibitor) and placebo treatments^23^. However, the specific neural mechanisms underlying the differential treatment responses observed across these subtypes remain to be fully elucidated. Lastly, while some studies have incorporated external validation cohorts, a significant portion of research still lacks rigorous validation, particularly concerning the consistency of treatment responses across identified subtypes. This gap limits the broader applicability and generalizability of the findings.

Depression is also a disease that evolves dynamically over time. Growing evidence suggests that the onset and progression of depression may evolve over the course of the illness^24–26^. In its early stages, depression is often characterized by subtle, nonspecific symptoms related to anxiety and mood disturbance, including worry, irritability, somatic pain, poor concentration, sadness, apathy, fatigue, sleep disruption, appetite changes, and psychomotor alterations. These features may progressively evolve into increased depressive symptoms (e.g., persistently low mood), ultimately culminating in chronic, treatment-refractory depression^27^. Furthermore, neuroimaging studies reveal dynamic changes in brain structure and function across different disease stages^28–30^. However, previous studies have primarily employed cross-sectional designs, deriving subtypes based solely on spatial patterns while overlooking clinically relevant illness-related variables (e.g., illness duration). The relationship between MDD subtypes and illness duration remains incompletely understood.

In this study, we focused on MDD subtyping and investigated how subtype characteristics evolve with illness duration and relate to treatment outcomes. First, we proposed a novel pre-training framework, BrainCVAE (**Brain C**ontrastive **V**ariational **A**uto**E**ncoder), which robustly captured MDD-specific features, and enabled identification of distinct MDD subtypes based on these features. Crucially, rather than using raw voxel-wise fALFF values directly, BrainCVAE utilizes fALFF maps as inputs to decode high-dimensional imaging data into disentangled latent representations. The specific disentanglement process, which isolates disease-unique variations from shared normative patterns, is illustrated in Fig. 6a. These features are designed to provide a more precise characterization of the underlying neural mechanisms of MDD, filtered out common physiological variance. Second, clinical outcomes following medication and rTMS were compared across patient subtypes. In addition, we used MDD-specific features to predict medication and rTMS treatment responses (i.e., to establish the predictive validity, in relation to treatment response). Third, we examined neuroimaging patterns and clinical characteristics across MDD subtypes in relation to illness duration. Finally, we validate the generalization and reliability using six independent datasets.

## Results

**Fig. 1:**
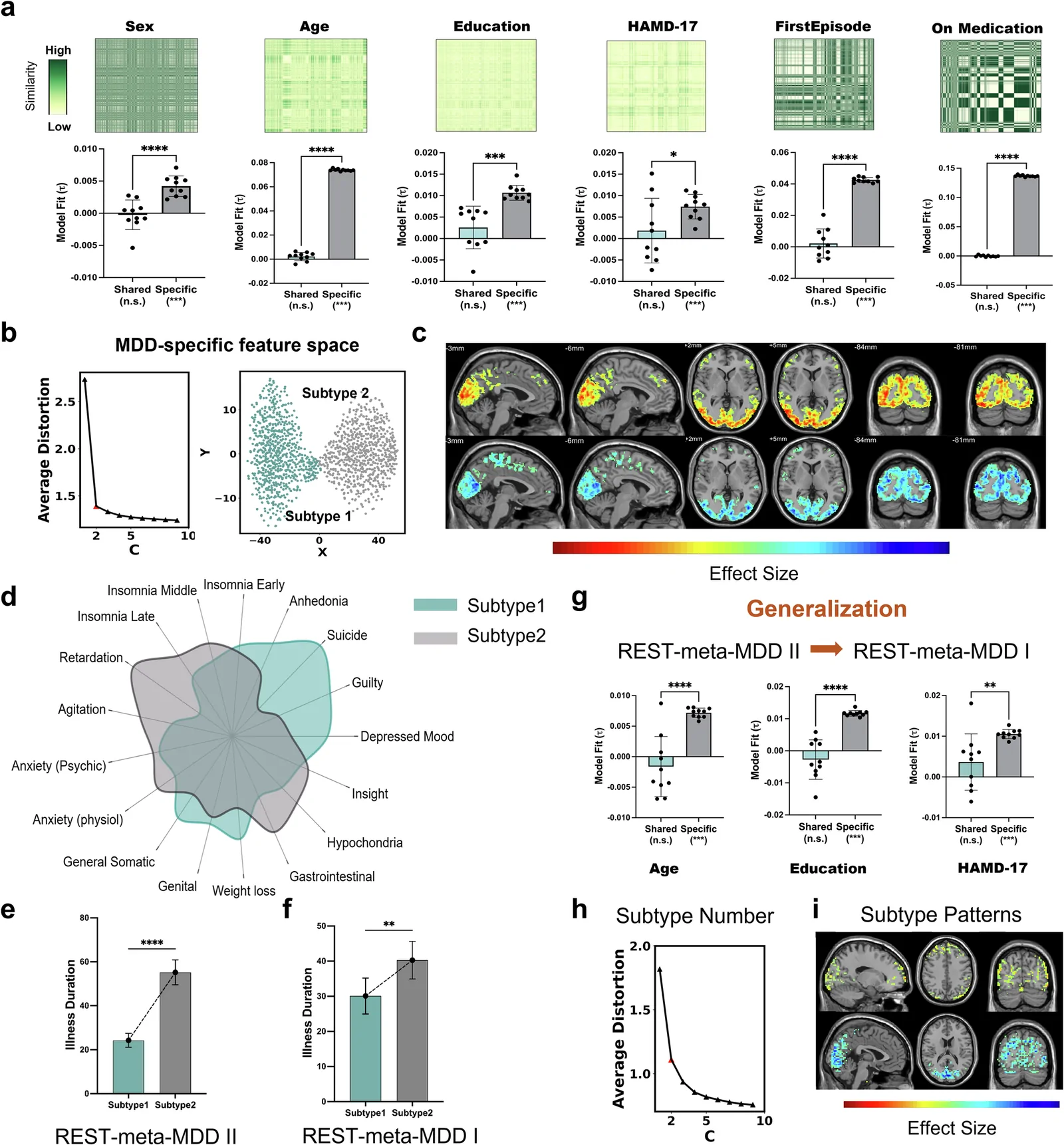
Brain Contrastive Variational Autoencoders (BrainCVAEs) identify two MDD subtypes. **a** Similarity of neurofunctional matrices and different clinical characteristics, including sex, age, education, HAMD-17, first episode and medication status. **b** Optimal number of subtypes. MDD-specific features are best captured by two clusters. Scatterplot depicts individual subjects’ functional data from subtype 1 (green) and subtype 2 (gray) groups, projected onto two-dimensional *t*-distributed stochastic neighbor embedding (*t*-SNE) dimensions computed from the MDD-specific features^79^. **c** Brain patterns of subtype 1 and subtype 2. False discovery rate (FDR)-corrected voxel-wise comparison of functional volume differences between subtype 1 (top row) and subtype 2 (bottom row) and controls are displayed on transverse, sagittal, and coronal sections. The color bar illustrates the degree of group differences (*t*-statistic) between distinct MDD subtypes and healthy control participants. **d** Clinical Profiles of subtype 1 and subtype 2. Summary results of the differences in 17 depressive symptoms between the two subtypes. Differences in illness duration across subtypes in both **e** REST-meta-MDD II and **f** REST-meta-MDD I datasets. **g** Generalization. The results generalize to a new dataset (REST-meta-MDD I). Participants with the same characteristics associated with increased risk of MDD are more similar in MDD-specific, but not shared. **h** Optimal clustering on the REST-meta-MDD I. **i** AlphaSim-corrected voxel-wise comparison of functional volume differences between subtype 1 (top row) and subtype 2 (bottom row) and controls on the REST-meta-MDD I. *p < 0.05, *** *p* < 0.001, ****p < 0.0001.

**Fig. 2:**
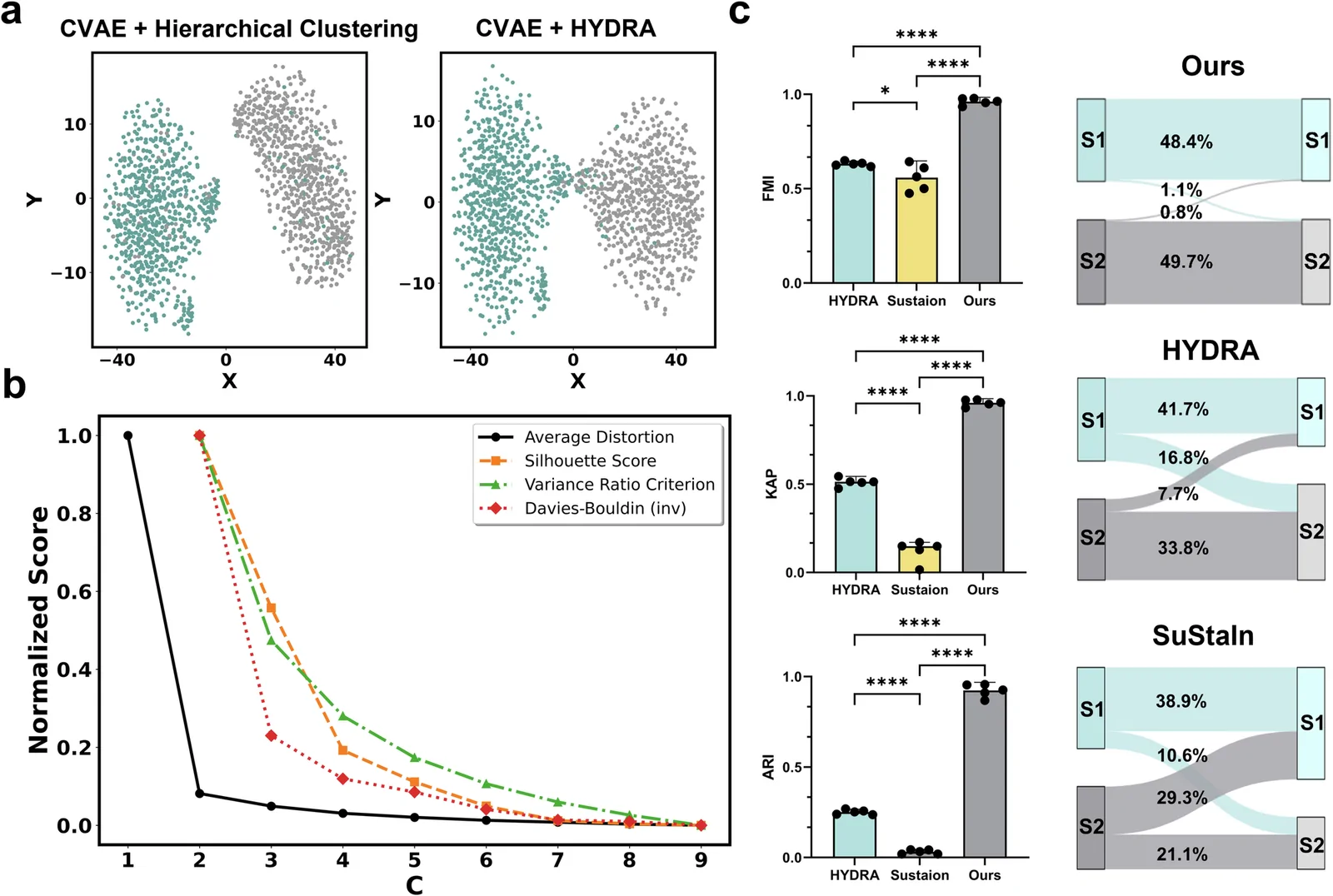
Reproducibility and reliability of subtype analysis. **a** t-SNE visualization of MDD subtypes identified using CVAE + Hierarchical Clustering (left) and CVAE + HYDRA (right). **b** The optimal number of clusters identified by multiple clustering validation metrics (Average Distortion, Silhouette Score, Variance Ratio Criterion, and Reverse Davies-Bouldin). **c** Comparison of clustering consistency and stability across three methods: HYDRA, SuStaIn and Ours. FMI Fowlkes–Mallows Index, KAP Cohen’s *κ*, and ARI Adjusted Rand Index, S1 subtype 1, S2 subtype 2. *p < 0.05, ***p < 0.001, ****p < 0.0001.

We used the largest global neuroimaging dataset of MDD (REST-meta-MDD II) to identify MDD-specific features and characterize MDD subtypes. Furthermore, we examined the relationship between these subtypes and treatment responses. Finally, we investigated the temporal evolution of pattern alterations in both subtypes. Table 1 demonstrates the demographic characteristics of participants across six datasets. REST-meta-MDD II was used for pre-training, our findings were validated across five independent external datasets. More details are provided in Supplementary Tables 2–12 and Figs. 1, 2.

**Table. 1:** Distribution of participants across the six cohorts

Cohort	Dataset 1	Dataset 2	Dataset 3	Dataset 4	Dataset 5	Dataset 6
MDD	1590	153	73	44	26	1248
Schizophrenia	0	0	0	0	0	0
Bipolar Disorder	0	0	0	0	0	0
Anxiety	0	0	0	0	0	0
Others	0	0	0	0	0	0
Medication	664	60	34	22	0	398
rTMS Treatment	0	0	39	22	26	0
HC	1308	97	0	0	0	1088
**Total**	**2898**	**250**	**73**	**44**	**26**	**2336**

Dataset 1 = REST-meta-MDD II, Dataset 2 = Xiangya, Dataset 3 = XJ-KG, Dataset 4 = XJ-QG, Dataset 5 = SAINT, Dataset 6 = REST-meta-MDD I major depressive disorder, *rTMS* repetitive transcranial magnetic stimulation, *HC* healthy controls.

### BrainCVAE reveals two optimal subtypes

To obtain MDD-specific features, we proposed BrainCVAE (see Methods) to separate MDD-specific features from shared features, ensuring that MDD-specific features correlated with clinical variations, while shared features were not associated with clinical changes. To validate the clinical relevance of MDD-specific features, we employed representational similarity analysis (RSA^31,32^, see Methods) to evaluate the association between the functional features generated by the BrainCVAE and individual variations in clinical characteristics among MDD patients (Fig. 1a). These characteristics included sex, age, education, first episode, medication status, as well as scores on the 17-item Hamilton Depression Rating Scale (HAMD-17). RSA demonstrated significantly enhanced Kendall τ correlations with clinical characteristics including sex (*t* = 5.04, *p* < 0.0001, *d* = 1.998, 95% CI = (0.0029, 0.0061), false discovery rate (FDR)-correction, Fig. 1a), age (*t* = 65.14, *p* < 0.0001, *d* = 20.618, 95% CI = (0.0692, 0.0742), FDR-correction, Fig. 1a), education (*t* = 4.87, *p* < 0.0001, *d* = 1.803, 95% CI = (0.0049, 0.0113), FDR-correction, Fig. 1a), HAMD-17 (*t* = 2.19, *p* = 0.047, *d* = 0.644, 95% CI = (−0.0006, 0.0118), FDR-correction, Fig. 1a), first episode (*t* = 13.55, *p* < 0.0001, *d* = 4.515, 95% CI = (0.0340, 0.0468), FDR-correction, Fig. 1a) and medication status (*t* = 210.60, *p* < 0.0001, *d* = 62.287, 95% CI = (0.1353, 0.1384), FDR-correction, Fig. 1a) in MDD-specific features compared with shared features. We further explored the relationship between head motion, age, education level, and brain activity (fALFF) in patients with MDD and healthy controls to validate our RSA results. The detailed findings are provided in the Supplementary Fig. 3-5.

Furthermore, we used K-means clustering methods to identify subtypes in patients with MDD based on MDD-specific features. The optimal number of subtypes was selected using the average distortion (Fig. 1b). A total of 802 patients with MDD were assigned to subtype 1, while 788 patients with MDD were assigned to subtype 2. In subtype 1, fALFF was significantly increased in middle occipital areas, parietal areas, middle and superior temporal areas and middle frontal areas compared with HCs (Fig. 1c). The converse was found in subtype 2: fALFF was significantly decreased in middle occipital areas, parietal areas, middle and superior temporal areas, and middle and superior frontal areas compared to HCs (Fig. 1c). The surface plots of both subtypes are provided in the Supplementary Fig. 6. These regions predominantly overlap with the visual network, attention network, and the posterior DMN subnetwork (precuneus).

### Subtype 1 and subtype 2 show different clinical profiles

To determine whether the subtypes exhibit distinct clinical symptom profiles, we compared the two groups across individual items of the 17-item Hamilton Depression Rating Scale (HAMD-17). We observed that subtype 1 predominantly manifests with affective symptoms, including anhedonia, suicide, guilty and depressed mood, while subtype 2 is more related with somatic symptoms, such as insomnia, retardation and gastrointestinal (Fig. 1d). Using Symptom Network Analysis clustering^33^ to corroborate our findings, we again observed that subtype 1 was characterized predominantly by affective symptoms, whereas subtype 2 was characterized predominantly by somatic symptoms (Supplementary Fig. 7). Additionally, significantly reduced illness duration was observed in subtype 1 compared with subtype 2 on the REST-meta-MDD II (*t* = −8.6013, *p* < 0.0001, *d* = −0.4916, 95% CI= (−37.3430, −23.4716), Fig. 1e) and the REST-meta-MDD I (*t* = −2.6089, *p* = 0.0092, *d* = −0.1706, 95% CI = (−17.8545, −2.5251), Fig. 1f).

### Generalization to an independent dataset

We tested generalization by performing an identical, independent analysis on the REST-meta-MDD I dataset, including 1248 MDD participants and 1088 HCs (Fig. 1g-i), without using features derived from REST-meta-MDD II. MDD-specific features similarly showed pronounced clinical relevance. Significantly reduced Kendall τ correlations with clinical characteristics including age (*t* = −5.585, *p* < 0.0001, *d* = −1.806, 95% CI = (−0.0123, −0.0053), FDR-correction, Fig. 1g), education (*t* = −7.338, *p* < 0.0001, *d* = −2.392, 95% CI = (−0.0187, −0.0101), FDR-correction, Fig. 1g) and HAMD-17 (*t* = −3.093, *p* = 0.0063, *d* = −0.982, 95% CI = (-0.0118, -0.0019), FDR-correction, Fig. 1g) were observed in shared features compared with MDD-specific features. Furthermore, we consistently identified two MDD subtypes (689 patients with subtype 1 and 599 patients with subtype 2) in the REST-meta-MDD I dataset (Fig. 1h). We observed a significant increase in fALFF in middle and inferior frontal, limbic areas in Subtype 1 compared to healthy controls, alongside a significant decrease in fALFF in middle occipital and temporal areas. The differences between Subtype 1 and Subtype 2 were primarily localized to the visual network, attention network, and the posterior DMN subnetwork, consistent with the functional patterns identified for these subtypes in the REST-meta-MDD II dataset (Fig. 1i). Supplementary Fig. 8 presents the glass brain representation of both subtypes in the REST-meta-MDD I dataset.

### Reproducibility and reliability of subtype analysis

**Fig. 3:**
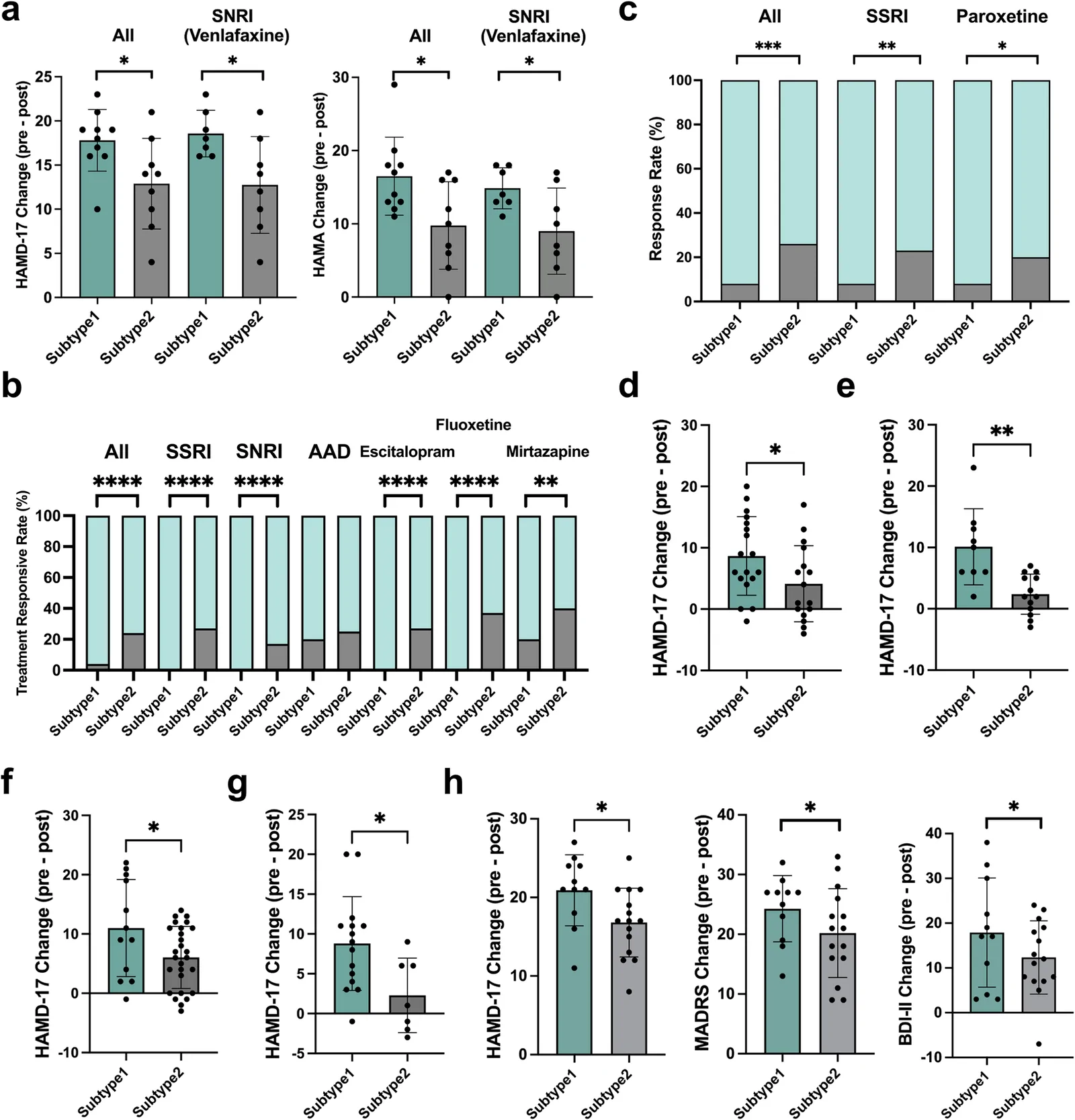
Clinical outcomes across the subtypes and different medication types on the REST-meta-MDD II, Xiangya, XJ-KG and XJ-QG datasets. **a** Difference in changes in HAMD-17 and HAMA across two subtypes following all medication treatment and SNRI Venlafaxine medication treatment in the 21-th site of REST-meta-MDD II. **b** Comparison of treatment responsive rate for all medication, medication-free, SSRI medication, SNRI medication, AAD medication and specific medications including Escitalopram, Fluoxetine and Mirtazapine between both subtypes in the first site of REST-meta-MDD II. **c** Comparison of response rate for all medication, SSRI medication and Paroxetine between both subtypes in the Xiangya dataset. The treatment response rate reflects the proportion of patients who are non-resistant to treatment. The response rate is defined as the proportion of responders or non-responders within the treated population. A responder is characterized by a reduction of ≥ 50% on the HAMD-17 following the specific medication treatment, while non-responders are those who do not meet this criterion. The blue bars represent the response rate (defined as the proportion of responders among all patients), while the gray bars represent the non-response rate (defined as the proportion of non-responders among all patients). **d** Difference in changes in HAMD-17 across two subtypes following SSRI sertraline medication treatment in the XJ-KG dataset. **e** Difference in changes in HAMD-17 across two subtypes following all medication treatment in the XJ-QG dataset. Depressive symptoms across the subtypes in three rTMS treatment groups including **f** XJ-KG, **g** XJ-QG and **h** SAINT dataset. The data is presented using a bar plot with mean values and standard deviation. The asterisks (*) indicate significant differences between the two subgroups using a two-sided Linear Mixed-Effects Model (LMM) with a *p*-value of less than 0.05. *p < 0.05, **p < 0.01, ***p < 0.001, ****p < 0.0001.

To assess the reproducibility and robustness of our clustering, we conducted three complementary validations. First, we extracted MDD-specific features using BrainCVAE, and subsequently re-analyzed these features with alternative algorithms (hierarchical clustering and HYDRA^34^). Across methods, two subtypes were reproduced consistently (Fig. 3a), and the labels from hierarchical clustering and HYDRA showed near-perfect concordance with those from K-means (disagreement <1%). Further details are provided in the Supplementary Figs. 9, 10.

Second, we compared various methods for determining the optimal number of clusters, including average distortion, silhouette score, variance ratio criterion, and Davies-Bouldin index (Fig. 3b). Our results showed that our method was highly stable and consistently identified two subtypes.

Third, to benchmark our approach against state-of-the-art subtyping methods (HYDRA^34^ and SuStaIn^35^), we evaluated the clustering stability of three methods using bootstrap resampling: in each iteration, 80% of the original dataset was randomly selected for re-clustering, and label changes were compared across iterations. The complete analysis pipeline was repeated 100 times, and a repeated-measures one-way analysis of variance (ANOVA) was performed on three metrics of the three methods: Fowlkes–Mallows Index (FMI^36^), Cohen’s *κ* (KAP^37^), and Adjusted Rand Index (ARI^38^). We observed a significant group effect for FMI (*F* (1.139, 4.557) = 109.5, *p* = 0.0002, ${\eta }^{2}$ = 0.9544, Fig. 2c), with significant effects likewise for KAP (*F* (1.145,4.580) = 462.2, *p* < 0.0001, ${\eta }^{2}$ = 0.9914, Fig. 2c) and ARI (*F* (1.370, 5.481) = 2564.1, *p* < 0.0001, ${\eta }^{2}$ = 0.9984, Fig. 2c). Post-hoc Tukey tests showed that our method significantly outperformed both HYDRA (FMI, *p* < 0.0001, MD (mean difference) = 0.330, 95% CI = (0.2929,0.3679); KAP, *p* < 0.0001, MD = 0.449, 95% CI = (0.3922,0.5046); ARI, *p* < 0.0001, MD = 0.672, 95% CI = (0.6137,0.7306)) and SuStaIn (FMI, *p* = 0.0007, MD = 0.403, 95% CI = (0.2803,0.5257); KAP, *p* < 0.0001, MD = 0.838, 95% CI = (0.7474,0.9289); ARI, *p* < 0.0001, MD = 0.892, 95% CI = (0.8463,0.9378)). The Sankey diagram depicted subtype label transitions across five repetitions for three methods, suggesting that our approach exhibited substantially greater stability than the alternatives. Further details about the HYDRA and SuStaIn models are available in the Supplementary Note 3. Interestingly, both HYDRA and SuStaIn likewise identified significant differences in illness duration between the two subtypes (Supplementary Fig. 9).

Finally, we investigated the influence of variables such as recurrent status, age, illness duration, medication, and sample size on our subtype analysis; the results can be found in Supplementary Fig. 11–14. The results indicated that our findings were robust and unaffected by these extraneous factors.

### Ablation study for the subtype analysis

We further conducted an additional ablation analysis focusing on key nodes of the default mode network (DMN), including the medial prefrontal cortex (mPFC), posterior cingulate cortex (PCC), left parietal cortex (LPC) and right parietal cortex (RPC). Specifically, we removed the voxels corresponding to each of these regions in turn from the fALFF input images and re-ran the BrainCVAE-based subtyping, thereby quantifying how the absence of each DMN node affected the stability of the subtyping analysis. We assessed the impact of each ablation by comparing the new subtype assignments with the original ones using three complementary clustering agreement metrics: the Fowlkes–Mallows index (FMI), Cohen’s *κ* (KAP) and the adjusted rand index (ARI), for which lower values indicate poorer clustering performance. As shown in Table 2, ablating the mPFC produced the largest decrease across all three indices, indicating that this region plays a particularly decisive role in distinguishing the two subtypes, whereas removal of PCC, LPC or RPC had comparatively smaller effects. These findings demonstrate that the BrainCVAE latent space can be meaningfully linked to specific DMN nodes.

**Table. 2:** Ablation study for subtype analysis using REST-meta-MDD II

Regions	mPFC	PCC	LPC	RPC
ARI	0.0076	0.3361	0.1391	0.1273
FMI	0.5041	0.7785	0.6723	0.6195
KAP	0.0911	0.3788	0.1438	0.1376

*mPFC* medial prefrontal cortex, *PCC* posterior cingulate cortex, *LPC* left parietal cortex and *RPC* right parietal cortex, *FMI* Fowlkes–Mallows Index, *KAP* Cohen’s *κ* and *ARI* Adjusted Rand Index.

### Subtype 1 and subtype 2 showed distinct responses to antidepressant treatment

We compared response rate and HAMD scores between subtypes using five independent datasets, including REST-meta-MDD II, Xiangya, XJ-KG, XJ-QG and SAINT datasets. Interestingly, subtype 1 consistently exhibited superior treatment response to both pharmacotherapy (SSRIs and SNRIs; Fig. 3a–e) and repetitive transcranial magnetic stimulation (rTMS; Fig. 3f–h).

For the medication treatment, patients with subtype 1 showed significantly enhanced 17-item Hamilton Depression Rating Scale (HAMD-17) changes following all medication treatment (subtype 1: 17.8 ± 3.5, subtype2: 12.9 ± 5.1; *t* = 2.537, *p* = 0.0331, *d* = 1.0803, 95% CI = (9.1201, 0.70208), FDR-correction) and SNRI medication treatment (subtype 1: 18.6 ± 2.6, subtype2: 12.8 ± 5.5; *t* = 2.831, *p* = 0.0331, *d* = 1.2461, 95% CI = (0.9064,10.7365), FDR-correction) compared with patients with subtype 2 in the 21-th site of REST-meta-MDD II (Fig. 3a). Similarly, significantly increased Hamilton Anxiety Rating Scale (HAMA) changes after all medication treatment (subtype 1: 16.5 ± 5.3, subtype 2: 9.8 ± 6.0; *t* = 2.622, *p* = 0.0331, *d* = 1.1391, 95% CI = (1.2584,12.1860), FDR-correction) and SNRI Venlafaxine medication treatment (subtype 1: 14.9 ± 2.8, subtype 2: 9.0 ± 5.9; *t* = 2.467, *p* = 0.0358, *d* = 1.1692, 95% CI = (0.5867,11.1276), FDR-correction) were observed in patients with subtype 1 compared to patients with subtype 2 (Fig. 3a). Moreover, we also verified our results with a retrospective and real-world dataset (first site of REST-meta-MDD II). Patients with subtype 1 were characterized by superior efficacy across all medication (treatment responsive rate of subtype 1 = 95.8%, treatment responsive rate of subtype 2 = 76.3%; ${\chi }^{2}$ = 16.61, *p* < 0.0001, FDR-correction), SSRI (Selective Serotonin Reuptake Inhibitor) medication (treatment responsive rate of subtype 1 = 100%, treatment responsive rate of subtype 2 = 73.7%; ${\chi }^{2}$ = 31.21, *p* < 0.0001, FDR-correction) and SNRI (Serotonin and Norepinephrine Reuptake Inhibitor) medication (treatment responsive rate of subtype 1 = 100%, treatment responsive rate of subtype 2 = 83.3%; ${\chi }^{2}$ = 18.58, *p* < 0.0001, FDR-correction) (Fig. 3b). For specific medications, patients with subtype 1 exhibited superior efficacy compared to patients with subtype 2 with SSRI Escitalopram (treatment responsive rate of subtype 1 = 100%, treatment responsive rate of subtype 2 = 72.7%; ${\chi }^{2}$ = 31.21, *p* < 0.0001, FDR-correction), SSRI Fluoxetine (treatment responsive rate of subtype 1 = 100%, response rate of subtype 2 = 62.5%; ${\chi }^{2}$ = 45.40, *p* < 0.0001, FDR-correction), AAD Mirtazapine (treatment responsive rate of subtype 1 = 80%, treatment responsive rate of subtype 2 = 60%; ${\chi }^{2}$ = 8.33, *p* = 0.0023, FDR-correction) (Fig. 3b). For the Xiangya dataset, patients with subtype 1 exhibited superior efficacy compared to patients with subtype 2 with all medication (response rate of subtype 1 = 92.0%, response rate of subtype 2 = 74.3%; ${\chi }^{2}$ = 11.48, *p* < 0.001, FDR-correction), SSRI medication (response rate of subtype 1 = 91.3%, response rate of subtype 2 = 71.4%; ${\chi }^{2}$ = 8.59, *p* = 0.0051, FDR-correction), SSRI Paroxetine (response rate of subtype 1 = 91.3%, response rate of subtype 2 = 80%; ${\chi }^{2}$ = 5.98, *p* = 0.0145, FDR-correction) (Fig. 3c). For the XJ-KG dataset, patients with subtype 1 showed significantly enhanced HAMD-17 changes following SSRI sertraline treatment (subtype 1: 8.7 ± 6.4, subtype2: 4.1 ± 6.2; *t* = 2.125, *p* = 0.0419, *d* = 0.7017, 95% CI = (0.1192, 8.9640), Fig. 3d). While for the XJ-QG dataset, patients with subtype 1 showed significantly enhanced HAMD-17 changes following all medication treatment (subtype 1: 10.1 ± 6.2, subtype2: 2.4 ± 3.3; *t* = 3.784, *p* = 0.0014, *d* = 1.5919, 95% CI = (3.0228, 10.9773)) compared with patients with subtype 2 (Fig. 3e). Here, the treatment response rate reflects the proportion of patients who are non-resistant to treatment. The response rate is defined as the proportion of responders within the treated population. A responder is characterized by a reduction of ≥50% on the HAMD-17 following the specific medication treatment, while non-responders are those who do not meet this criterion.

Furthermore, we further compared the differences in depressive symptoms between subtype 1 and subtype 2 after undergoing rTMS treatment on three independent datasets (XJ-KG, XJ-QG and SAINT datasets). Specifically, for the XJ-KG, patients with subtype 1 showed significantly enhanced HAMD-17 changes after rTMS treatment (subtype 1: 11.0 ± 8.2, subtype2: 6.0 ± 5.2; *t* = 2.129, *p* = 0.0404, *d* = 1.7763, 95% CI = (0.5608, 9.3651)) compared with patients with subtype 2 (Fig. 3f). For the XJ-QG, patients with subtype 1 showed significantly enhanced HAMD-17 changes after rTMS treatment (subtype 1: 8.8 ± 5.9, subtype2: 2.3 ± 4.7; *t* = 2.154, *p* = 0.0450, *d* = 1.1275, 95% CI = (1.3514,11.2486)) compared with patients with subtype 2 (Fig. 3g). Furthermore, for the SAINT dataset, patients with subtype 1 showed significantly enhanced HAMD-17 (subtype 1: 20.9 ± 4.5, subtype2: 16.8 ± 4.4; *t* = 2.972, *p* = 0.0225, *d* = 1.021, 95% CI = (1.0153,8.0998), FDR-correction), Montgomery-Asberg Depression Rating Scale (MADRS) (subtype 1: 24.3 ± 5.6, subtype2: 20.2 ± 7.4; *t* = 2.404, *p* = 0.0386, *d* = 0.807, 95% CI = (0.0880,10.6756), FDR-correction) and Beck Depression Inventory-II (BDI-II) scores (subtype 1: 17.9 ± 12.2, subtype 2: 12.3 ± 8.2; *t* = 2.214, *p* = 0.0386, *d* = 0.767, 95% CI = (0.2669,15.5882), FDR-correction) changes after rTMS treatment for 5 days compared with patients with subtype 2 (Fig. 3h).

Notably, during external validation, we did not retrain the model; instead, we applied the REST-meta-MDD II-trained BrainCVAE to the functional images of participants from external datasets using a parameter-free approach (without retraining or transfer learning). This methodology demonstrates substantial potential for clinical translation.

### Interaction between different subtypes and illness duration

We further explored how patterns associated with distinct depression subtypes vary in relation to illness duration. Specifically, we examined the relationship between different subtypes and illness duration across three dimensions: spatial brain patterns, symptom profiles, and treatment outcomes.

**Fig. 4:**
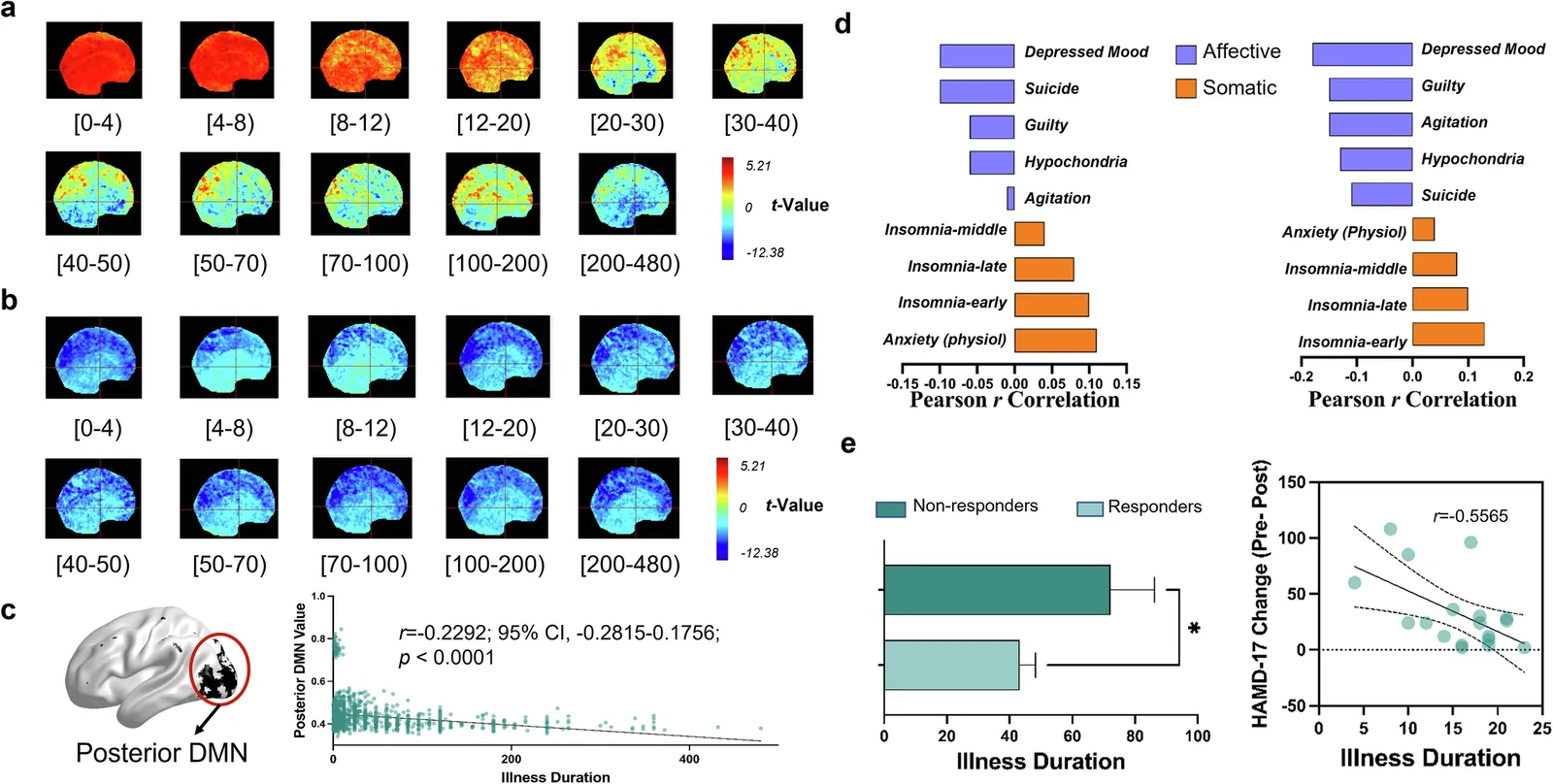
Association between different subtypes and illness duration. Brain patterns across illness duration in both subtypes. False discovery rate (FDR)-corrected voxel-wise evolution of functional volume differences between **a** subtype1, **b** subtype2 and HCs are displayed on a sagittal section following distinct illness duration intervals. Illness duration intervals are based on the distribution of illness duration (Supplementary Fig. 2). The color bar illustrates the degree of group differences (*t*-statistic) between MDD patients and healthy control participants. **c** Association between the mean functional value of the posterior default mode network (DMN) and illness duration. **d** Pearson’s correlation between each item of HAMD-17 and illness duration in both REST-meta-MDD II (left) and REST-meta-MDD I datasets (right). Psychiatric symptoms are represented in blue, while physical symptoms are shown in orange. **e** Comparison of illness duration between non-responders and responders, and the relationship between changes in HAMD-17 scores following medication treatment and illness duration.

For the spatial brain patterns, we compared the differences in functional patterns between patients with different subtypes and HCs across different illness duration intervals (Fig. 4a, b) on the REST-meta-MDD II. In subtype 1, brain patterns were consistent with a shift from relatively hyperactive to hypoactive profiles across illness duration (Fig. 4a), whereas subtype 2 remained consistently hypoactive across illness duration (Fig. 4b). Moreover, within the posterior DMN network (precuneus), the mean functional image value exhibited a significant negative correlation with illness duration (Pearson’s *r* = -0.2292, *p* < 0.0001, confidence interval (CI) = (-0.2815, 0.1756), Fig. 4c). The observed pattern suggests that, in our cross-sectional sample, individuals classified as Subtype 1 tend to show relatively hyperactive brain features in earlier illness stages and more hypoactive brain features in later stages. However, this pattern is derived from cross-sectional stratification rather than longitudinal follow-up, and therefore should be interpreted as an indirect inference rather than direct evidence of within-subject progression.

For the symptom profiles, we investigated the Pearson correlation between the 17-HAMD symptoms and the duration of the illness. We observed that affective symptoms, including depressed mood and guilty, progressively diminished as the illness advanced, whereas somatic symptoms, such as insomnia, tended to exacerbate over time (Fig. 4d).

We further examined the association between illness duration and treatment response. Specifically, we compared the differences in illness duration between non-responders and responders, and examined whether changes in HAMD-17 scores following medication treatment were associated with illness duration. Significantly reduced illness duration was observed in responders compared with non-responders on the REST-meta-MDD II (*t* = 2.364, *p* = 0.0191, mean responders = 43.20, mean non-responders = 72.15, Fig. 4e). A significant negative correlation was found between HAMD-17 changes and illness duration on the REST-meta-MDD II (Pearson’s *r* = −0.5565, *p* = 0.0165, CI = (−0.8123, −0.1210), Fig. 4e).

### Predicting treatment outcomes using MDD-Specific features

**Fig. 5:**
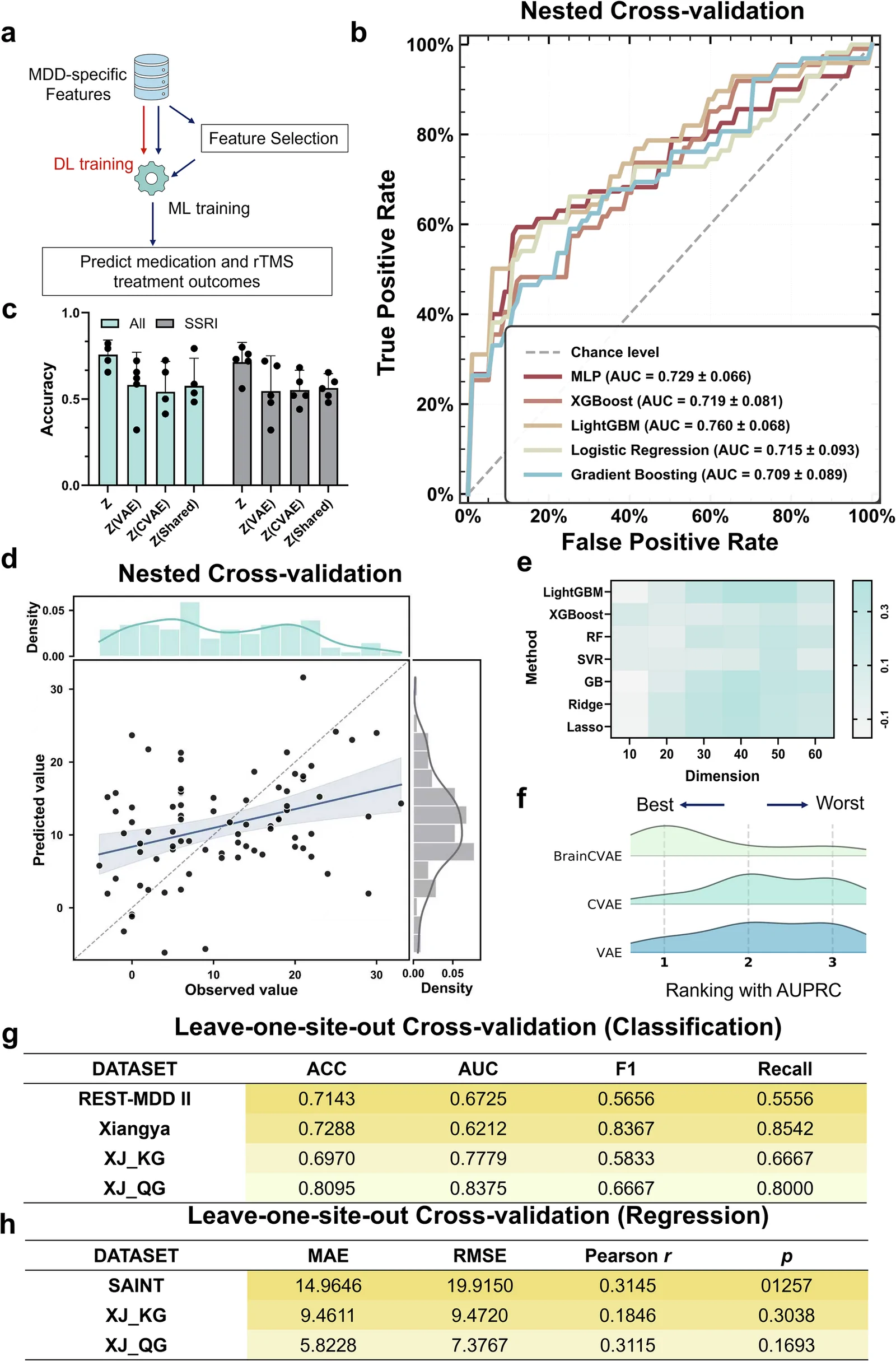
MDD-specific features disentangled from BrainCVAE characterize antidepressant response. **a** Overall scheme of treatment-response methods using machine-learning (ML) and deep-learning (DL) process. **b** ROC curves and accuracy results of DL and various ML methods for nested cross-validation classification of medication responders versus non-responders. **c** Accuracy comparison across different features (Z: MDD-specific features from BrainCVAE; Z_VAE_: features from VAE; Z_CVAE_: MDD-specific features from CVAE; Z_Shared_: shared features from BrainCVAE) for all medications (green) and SSRI medications (gray). **d** Nested cross-validation regression for HAMD-17 changes after rTMS. Central scatter plot shows observed vs. predicted values with regression line and 95% CI. Top and right panels display marginal distributions (histogram and kernel density estimate) of observed and predicted values, respectively. **e** Heatmap showing impact of feature dimensionality reduction from 60 to 10 on rTMS regression performance across different ML methods. **f** Ranking distributions of BrainCVAE, CVAE, and VAE based on AUPRC performance. **g** Leave-one-site-out cross-validation for classification across four datasets, showing accuracy (ACC), AUC, F1 score, and recall. **h** Leave-one-site-out cross-validation for regression across four datasets, showing MAE, RMSE, Pearson’s *r*, and *p*-values. RF random forest, SVR support vector regression, GB gradient boosting.

To further evaluate the clinical utility of the identified MDD-specific features, we assessed their potential as neuroimaging biomarkers for predicting treatment outcomes following medication and rTMS. Specifically, we implemented both machine learning (ML) and deep learning (DL) pipelines. In the DL pipeline, we directly used a two-layer multi-layer perceptron (MLP) as the classifier. In the ML pipeline, MDD-specific features were first subjected to dimensionality reduction and the resulting representations were then entered into downstream classifiers or regressors (Fig. 5a). To identify the optimal configuration, we systematically explored multiple model families: classifiers were drawn from eXtreme Gradient Boosting (XGBoost^39^), Light Gradient Boosting Machine (LightGBM^40^), logistic regression^41^, and gradient boosting (GB^42^); regressors from LightGBM, XGBoost, random forest (RF^43^), support vector regression (SVR^44^), ridge regression^45^, and least absolute shrinkage and selection operator (LASSO) regression^46^; and dimensionality reduction methods from principal component analysis (PCA^47^), ANOVA, and factor analysis^48^. The final model was defined as the combination of dimensionality reduction method and classifier/regressor that yielded the best performance under grid search.

All experimental results are reported based on nested cross-validation using three outer and five inner folds (Supplementary Fig. 15). The classification results are shown in the ROC curve in Fig. 5b, where we compared a range of DL and ML methods and found that the MLP achieved the highest accuracy of 75.74% (All medication: ACC = 0.7574 ± 0.0603, AUC = 0.7290 ± 0.0663, F1 = 0.7282 ± 0.0626, Recall = 0.6716 ± 0.0730, permutation test-validated using 1000 permutations, *p* < 0.001; SSRI medication: ACC = 0.7142 ± 0.0819, AUC = 0.7199 ± 0.0794, F1 = 0.6743 ± 0.1146, Recall = 0.6162 ± 0.1451, permutation test-validated using 1,000 permutations, *p* < 0.001).

Furthermore, we further developed ridge regression model combined with PCA to predict the changes in HAMD-17 following rTMS treatment. MDD-specific features were used to predict the treatment outcome with nested cross-validation with 3 outer and 5 inner folds (Fig. 5d; MAE = 7.4772±1.1722, RMSE = 9.4667±4.2272, Pearson’s *r* = 0.4131±0.1129, *p* = 0.1414±0.1116, permutation test-validated using 1000 permutations, *p* < 0.05). We further evaluated the relationship between ML model performance and the number of PCA components retained, which served as a basis for determining the optimal dimensionality for feature reduction (Fig. 5e).

To assess the generalizability of our features, we conducted leave-one-site-out cross-validation for both regression and classification (Fig. 5g-h). Our results demonstrated a certain degree of generalizability; however, the prediction of rTMS outcomes was not effective. This indicates that MDD-specific features may hold greater promise as biomarkers for medication treatment response. Moreover, under identical configurations, features extracted from BrainCVAE demonstrated superior performance compared to those from VAE and CVAE, highlighting the enhanced predictive power of BrainCVAE for treatment outcomes (Fig. 5c, f).

To assess clinical relevance, we benchmarked our proposed biomarker against several established alternatives: (i) whole-brain functional connectivity (FC); (ii) default-mode network (DMN) FC; (iii) graph-theoretical indices including betweenness centrality, degree, clustering coefficient, global efficiency and local efficiency; and (iv) clinical predictors (age, sex and pre-treatment Hamilton Depression Rating Scale (HAMD) scores). For robustness and to ensure a fair comparison, we trained all models using an identical two-layer multi-layer perceptron (MLP) pipeline. As summarized in Table 3, the MDD-specific features achieved the highest classification accuracy, supporting the added value of our biomarker and underscoring its potential clinical utility.

**Table. 3:** Nested cross-validation performance across different biomarkers in antidepressant treatment

Biomarkers	FC (Whole Brain)	FC (DMN)	Graph-theoretical measures	Clinical predictors	Ours
ACC	0.62	0.61	0.50	0.63	**0.76**
AUC	0.59	0.59	0.47	0.61	**0.73**
F1	0.40	0.47	0.32	0.38	**0.73**

*FC* functional connectivity, *DMN* default mode network.

## Discussion

In this study, we proposed BrainCVAEs to identify MDD-specific features. Based on these features, we uncovered two distinct subtypes of MDD. Subtype 1 was characterized by activation of the visual network, attention network and posterior DMN, whereas subtype 2 exhibited these networks suppression. Additionally, we found that subtype 1 is more sensitive to antidepressant treatment compared to subtype 2. Furthermore, we observed significant differences across subtypes along the illness duration dimension. Finally, we found that the MDD-specific features could be used as a neuroimaging biomarker for predicting treatment outcomes.

The methodological contributions of BrainCVAE can be summarized across four dimensions: representation, clustering, staging and reproducibility. In terms of representation, BrainCVAE effectively removes redundant information and identifies disease-relevant features. By comparing the common features and MDD-specific features extracted by CVAE, we found that only our method successfully distinguishes between the two. This advantage may stem from the introduction of a novel discriminative loss (HSIC), which calculates the mutual information between the two feature types without requiring knowledge of the data distribution (PDF) or the incorporation of additional neural networks. This enables more accurate computations, eliminates information loss, and significantly enhances the stability of model training.

In clustering, our framework provides a scalable and robust solution for delineating heterogeneity in large neuroimaging datasets. By adopting simple yet highly scalable clustering strategies such as K-means, the method maintains computational efficiency even when applied to high-dimensional feature spaces, enabling analyses at both ROI and voxel levels without compromising performance. Because cluster structure is derived directly from the intrinsic organization of the data, the resulting subgroups naturally capture meaningful variations across individuals. Furthermore, by integrating clustering with subsequent subtype and phenotype characterization, the approach yields subgroups that are both interpretable and clinically informative, offering a coherent representation of disease-related heterogeneity.

For staging, our framework infers progression patterns directly from the identified subtypes, allowing stage definitions to emerge organically from data-driven characteristics rather than from externally imposed thresholds. This strategy produces progression trajectories that more accurately reflect population-level diversity. By examining how subtype-specific features vary across the inferred stages, the method can reveal potential temporal patterns even for disorders such as MDD, where structural or functional imaging changes are subtle and heterogeneous. Grounding stage transitions in coherent and data-driven phenotypic profiles enhances interpretability and provides a flexible mechanism for characterizing potential disease evolution.

Regarding reproducibility, we trained on the largest available dataset and tested on the largest validation set, achieving reproducible conclusions. Additionally, we applied a parameter-free approach (without retraining or transfer learning) to several smaller datasets, which also yielded reproducible results. These findings underscore the robustness and reproducibility of our method.

We proposed BrainCVAE to obtain MDD-specific features. Based on these features, we identified two distinct MDD subtypes. Subtype 1 was characterized by enhanced activity in the visual network, attention network, and posterior DMN, while Subtype 2 exhibited suppression of these networks. Our findings are in line with previous studies^29,49^, revealing that the DMN is functionally impaired in MDD relative to healthy controls. Our results confirm the critical role of the DMN in the pathophysiology of MDD. Importantly, our findings offer a deeper understanding of the inconsistencies reported in prior research. While previous studies have reported conflicting results regarding DMN activity—some showing increased activity^49–51^ and others indicating reduced activity^29^—our study offers new insights by suggesting the existence of two distinct DMN subtypes: one characterized by heightened activity and the other by diminished activity.

We identified 2 MDD subtypes using BrainCVAE, which is in line with previous studies. Most MRI-based studies have identified two MDD subtypes, with subtype classification primarily based on functional connectivity and T1-weighted images (Supplementary Table 1). Moreover, in addition to MRI studies, Zhang et al^52^. also used high-density resting-state electroencephalography to identify two MDD subtypes and discovered unique functional connectivity patterns in the frontoparietal control network and DMN. Taken together, these findings suggest that a two-subtype framework may capture an important and relatively robust dimension of the biological heterogeneity of MDD.

Previous subtype studies collectively highlight that MDD is a profoundly heterogeneous disorder, and that this heterogeneity is reflected not only in symptom presentation but also in resting-state activity, task-evoked neural responses, and large-scale connectivity patterns. Neuroimaging-driven work, including Tozzi et al^15^., suggests that biologically grounded subtypes may capture clinically relevant variation and may partially explain why patients with apparently similar diagnoses often respond differently to the same treatment. Such findings are particularly important because they provide a potential mechanistic framework for understanding inconsistent responses to pharmacotherapy and neuromodulation, and may also shed light on treatment resistance in a subset of patients. In this sense, subtype research represents an important step toward precision psychiatry.

However, the existing literature remains far from convergent. One major challenge is that the number and nature of reported subtypes differ considerably across studies, with no clear consensus regarding the latent structure of MDD. This variability likely reflects methodological and sample-related factors, including cohort size, population characteristics, modality selection, analytic pipelines, and clustering assumptions, rather than a stable underlying taxonomy alone. Moreover, despite the growing number of subtype studies, only a limited subset has examined whether identified subtypes reliably predict treatment response in a prospective and clinically actionable manner^6,15,17^. Therefore, while current findings strongly support the relevance of biological heterogeneity in MDD, they also suggest that subtype models should be interpreted cautiously until they demonstrate reproducibility across datasets and clearer links to treatment selection and clinical outcomes.

We further examined the relationship between distinct subtypes and treatment response. Patients with subtype 1 showed superior treatment outcomes compared with those in subtype 2. These results align with a previous study identifying MDD subtypes linked to differential responses to rTMS^17^. Additionally, HYDRA has revealed subtypes that show distinct responses to SSRI (Selective Serotonin Reuptake Inhibitor) and placebo^23^. Compared to prior studies, our findings demonstrate a larger differential in treatment sensitivity between subtypes, underscoring the advantage of our approach. BrainCVAE more efficiently identifies features that are closely tied to the core nature of MDD, thereby providing a deeper insight into the biological mechanisms driving the disorder’s heterogeneity. Additionally, we employed three external validation datasets to confirm the robustness of our findings. Subtype 1 showed significantly better treatment outcomes than subtype 2 across different sites and treatment strategies, including both medication and non-medication treatment (rTMS).

The differential treatment responses observed between subtype 1 and subtype 2 may be attributed to distinct functional disturbances inherent to each subtype. In this study, subtype 1 showed increased functional activity in the visual network, attention and posterior DMN (precuneus) compared with HCs, while subtype 2 showed decreased functional activity in these areas. In addition, patients with subtype 1 were more sensitive to SSRI escitalopram, SSRI Fluoxetine and SNRI Venlafaxine medication than patients with subtype 2. Extensive evidence indicates that the antidepressant medication (e.g., escitalopram) could exert its effects through the modulation of specific brain regions, particularly the precuneus^53–56^. For example, escitalopram could attenuate precuneus activity during self-evaluation in healthy controls^55^. In addition, escitalopram could alleviate depressive symptoms by reducing precuneus activity^53,56^. Furthermore, there is evidence suggesting that the regulatory mechanism of rTMS is also associated with the modulation of the precuneus^57,58^. rTMS treatment has been demonstrated to diminish functional connectivity between the dorsolateral prefrontal cortex and the precuneus in patients with depression^57^. Low-frequency rTMS led to a reduction in activity in the left precuneus among responders, an effect not observed in non-responders^58^. Thus, the superior treatment response observed in subtype 1 compared to subtype 2 may be attributed to the attenuation of excessive activation in the precuneus by the medication and rTMS.

In this study, we found that brain spatial patterns and clinical symptoms varied across subtypes in relation to illness duration. These observations align with prior studies indicating that MDD is characterized by heterogeneity across illness course, involving alterations in both brain function and structure^28,29^. Clinically, a prolonged course of MDD is predictive of persistent depressive symptoms and a heightened risk of chronicity^59^. The severity of symptoms intensifies with an increasing number of episodes^60^. We found that patients with longer illness duration exhibited relatively greater somatic symptom burden compared to affective symptoms, suggesting potential illness stage–related differences in symptom profiles.

Thus, we further examined the relationship between MDD subtypes and illness duration. We observed that subtype-specific patterns differed across illness duration: patients with subtype 1 showed patterns consistent with a shift from relatively hyperactive to hypoactive brain activity profiles, whereas subtype 2 remained consistently characterized by hypoactive patterns. These findings suggest that subtype-specific patterns may vary systematically with illness duration. Previous studies have proposed that depressive subtypes may reflect positions along a continuum of severity rather than entirely distinct disease entities^25^. Our findings are broadly consistent with this framework and may provide potential markers related to illness staging, although longitudinal validation is required.

Furthermore, the duration of illness in subtype 1 was significantly shorter than in subtype 2, and individuals with subtype 1 appeared more responsive to treatment. These findings may reflect differences associated with illness duration across subtypes, with subtype 2 potentially representing a more treatment-resistant profile. In addition, we examined the relationship between treatment response and illness duration. Responders had significantly shorter illness duration compared to non-responders, and changes in HAMD scores following treatment were positively associated with illness duration. These results suggest that longer illness duration is associated with reduced treatment responsiveness in MDD. Previous studies have similarly reported that prolonged illness duration is linked to poorer treatment outcomes^61–63^. Together, these findings underscore the importance of early detection and timely intervention in improving treatment outcomes in depression.

Our findings have direct implications for how precision psychiatry can be operationalized in clinical trials and practice. In the context of depression treatment, particularly neuromodulation such as rTMS, illness duration (staging) should be systematically integrated with BrainCVAE-derived neurobiological subtypes within the clinical decision-making framework. Targets identified from neuroimaging-based computations at a single time point are unlikely to remain stable over a prolonged disease course and are therefore better conceptualized as dynamic neuromodulation targets that require updating as the illness progresses. The observation that differences between short-course and long-course subtypes cannot be attributed solely to baseline symptom severity further indicates that illness duration constitutes an additional, clinically meaningful dimension of heterogeneity beyond symptom burden.

These insights motivate a shift in clinical trial design. Whereas current pharmacological and neuromodulatory strategies are largely guided by symptom profile and severity, our data suggest that illness duration may be equally important, or in some contexts even more critical, for shaping treatment response. This challenges existing treatment heuristics and supports the use of staging-informed stratification in future studies. We propose that randomized controlled trials incorporate both subtype and stage as stratification variables and explicitly consider interactions between these two dimensions when evaluating treatment effects. For example, patients in the short course subtype with hyperactive default mode, visual and attention networks may be preferentially enriched in trials testing SSRI, SNRI or rTMS protocols targeting these systems, whereas patients in the long course subtype with persistently hypoactive default mode profiles may require alternative neuromodulation targets, higher treatment intensity or combined interventions.

Finally, our framework suggests a practical translational workflow. Resting state fMRI data can be entered into the BrainCVAE model to generate an individual-level subtype plus stage profile, which in turn can guide modality choice (pharmacological versus neuromodulatory), network-specific target selection and the dynamic adjustment of neuromodulation parameters over the course of illness.

The limitations of this study should be considered. First, we observed that subtype 2 exhibits limited responsiveness to both medication and rTMS. Whether this subtype is similarly resistant to other non-pharmacological treatments, such as Cognitive Behavioral Therapy (CBT), requires further investigation. Second, our interpretation of a potential shift from hyperactive to hypoactive DMN patterns in subtype 1 is based on cross-sectional comparisons and therefore does not allow inference about within-subject temporal progression. Longitudinal studies will be required to directly test and validate these observations. Third, we interpret the relationships between peripheral markers and the illness course as being disease-related, further studies that directly address these potential confounders (such as controlled trials or longitudinal studies) are necessary to validate these findings and explore the role of medication and comorbid conditions more rigorously. Fourth, due to the lack of detailed pharmacological information (e.g., dosage, duration, and medication classes), medication was modeled as a dichotomous variable, and its potential effects on subtype identification and stability could not be fully assessed. Fifth, the relatively small treatment cohort, particularly for rTMS, may limit the generalizability of treatment-response findings. Sixth, the mixed observational and interventional design may introduce heterogeneity that could influence the interpretation of treatment effects. Finally, additional research is needed to determine the applicability of our method for other mental disorders.

In this study, we identified two MDD subtypes with distinct treatment responses using contrastive learning. Subtype 1, characterized by heightened dysfunction in the visual, attention, and posterior DMN networks and predominantly psychiatric symptoms, showed greater responsiveness to pharmacotherapy and rTMS than subtype 2, which exhibited reduced network dysfunction and more pronounced somatic symptoms. Subtypes were strongly associated with illness duration, with distinct patterns in symptoms and brain activity observed across illness duration. In subtype 1, shorter illness duration was associated with relatively higher brain activity and greater treatment responsiveness, whereas longer illness duration was linked to more hypoactive patterns. In contrast, subtype 2 remained consistently hypoactive across illness duration and showed reduced responsiveness. These findings highlight subtype-specific differences associated with illness duration and are broadly consistent with stage-related heterogeneity in MDD.

## Methods

### Participants

In this study, we used 6 different cohorts, including REST-meta-MDD I, REST-meta-MDD II, Xiangya, XJ-KG, XJ-QG and SAINT. The XJ_QG, XJ_KG, and SAINT datasets were approved by the Ethics Committee of the First Affiliated Hospital, Fourth Military Medical University (Approval No. XJLL-KY20222111, KY20222165-F-1, and KY20202066-F-1, respectively) and registered at ClinicalTrials.gov (NCT05577481, NCT05544071, and NCT04653337, respectively). The Xiangya dataset was approved by the Ethics Committee of Zhumadian Psychiatric Hospital (Approval No. (2013) Ethics Review No. (002)) and registered in the Chinese Clinical Trial Registry (ChiCTR1800014591). The REST-meta-MDD I and REST-meta-MDD II datasets were obtained from public de-identified datasets, with the original studies approved by the local ethics committees of the participating sites.

Data of REST-meta-MDD I and REST-meta-MDD II collected by REST-meta-MDD Project from the Depression Imaging REsearch ConsorTium (DIRECT) are currently the largest resting-state fMRI datasets for MDD. In this study, we used a sample of (*N* = 2898, 1590 MDDs/1308 HCs) on the REST-meta-MDD II and a sample of (*N* = 2336, 1248 MDDs/1088 HCs) on the REST-meta-MDD I. Demographic data for REST-meta-MDD I and REST-meta-MDD II are presented in Supplementary Table 2 and 3, respectively.

Xiangya dataset was collected by the Zhumadian Psychiatric Hospital and its nearby communities from 2013 to 2018. According to inclusion and exclusion criteria, we selected a sample of (I = 60, 60 pre-treatment MDDs and corresponding post-treatment MDDs) from 153 patients with MDD at Xiangya. Demographic data for Xiangya are presented in Supplementary Table 4.

XJ-KG dataset is collected by the Department of Psychiatry at the First Affiliated Hospital, Air Force Military University. All participants were MDD patients with symptoms of anhedonia. In this study, we used a sample of (*N* = 73, 39 patients received the Stanford Accelerated Intelligent Neuromodulation Therapy (SAINT^64^) and 34 patients received SSRI sertraline medication) on the XJ-KG. Demographic data for XJ-KG are presented in Supplementary Table 5.

XJ-QG dataset is collected by the Department of Psychiatry at the First Affiliated Hospital, Air Force Military University. All participants were MDD patients with symptoms of emotional blunting. In this study, we used a sample of (*N* = 44, 22 patients received the Stanford Accelerated Intelligent Neuromodulation Therapy (SAINT) and 22 patients received SSRI or SNRI medication) on the XJ-QG. Demographic data for XJ-QG are presented in Supplementary Table 6.

SAINT dataset is collected by the Department of Psychiatry at the First Affiliated Hospital, Air Force Military University. All participants received the Stanford Accelerated Intelligent Neuromodulation Therapy (SAINT). In this study, we used a sample of (*N* = 26, 26 pre-treatment MDDs and corresponding post-treatment MDDs) on the SAINT. Demographic data for SAINT are presented in Supplementary Table 7.

More details of REST-meta-MDD, Xiangya, XJ-KG, XJ-QG and SAINT datasets are described in Supplementary Note 2.

### Preprocessing

Standard preprocessing of the REST-meta-MDD I and REST-meta-MDD II were done using the Data Processing Assistant for Resting-State fMRI (DPARSF, http://rfmri.org/DPARSF). Functional fMRI data of Xiangya, XJ-KG, XJ-QG and SAINT were preprocessed using the Graph Theoretical Network Analysis (GRETNA, https://www.nitrc.org/projects/gretna). Preprocessing steps encompassed discarding the initial 10 volumes, slice-timing correction, head motion correction, space normalization, and temporal bandpass filtering (0.01-0.1 Hz). The Friston 24-parameter model was employed to regress out head motion effects and linear regression was applied to eliminate the influences of white matter (WM), cerebrospinal fluid (CSF) signals, and linear trends. Furthermore, we used CompCor method^65^ which extracted the first 5 principal components from a combined WM/CSF mask and feed these 5 components into nuisance regressors. Finally, we applied scrubbing^66–68^ within nuisance covariates regression, by identifying “bad” time points using a threshold of FD > 0.2 mm. Each “bad” time point was then modeled as a separate regressor in the nuisance regression model. Note that while the data from REST-meta-MDD I is publicly available (https://rfmri.org/REST-meta-MDD), the raw data was not shared, precluding us from performing CompCor and scrubbing preprocessing steps.

### Calculation of fractional amplitude of low-frequency fluctuations (fALFF)

We utilized DPARSF to calculate the fALFF. In particular, the filtered time series of a voxel underwent a fast Fourier transform process to convert it to the frequency domain. Subsequently, the ALFF value for the voxel was determined as the average square root of the power spectrum. To improve specificity and sensitivity, the fALFF value was calculated as the ratio of the power in the specific frequency band (0.01–0.1 Hz) to that of the entire detected frequency range. This approach aimed to suppress nonspecific signals in the resting-state fMRI data.

### Harmonization of site differences and covariates

To address site-specific artifacts and confounding effects, we employed the ComBat harmonization method^69,70^ to control for site differences and covariates in fALFF. This approach allowed us to preserve biological variability while eliminating site-induced variation. For each voxel value of fALFF, Combat model can be written as follows:

$$y={const}+{X}^{T}\beta +\gamma +\delta \varepsilon ,$$

in which const represents the constant term; ${X}^{T}$ is a design matrix for the covariates of interest (diagnosis, age, sex, education, head motion and medication dichotomous status (w/wo)); β is the vector of coefficients associated with X; γ represents additive site effects (location parameter), while δ denotes multiplicative site effects (scale parameter).

Accordingly, the harmonized voxel values of fALFF were defined as:

$${y}^{{Combat}}=\frac{y-\hat{{const}}-{X}^{T}\hat{\beta }-\hat{\gamma }}{\hat{\delta }}+\hat{{const}}+{X}^{T}\hat{\beta },$$

where $\hat{\delta }$ and $\hat{\gamma }$ represent the empirical Bayesian estimates of δ and γ, respectively.

### Architecture of brain contrastive variational autoencoder (BrainCVAE)

**Fig. 6:**
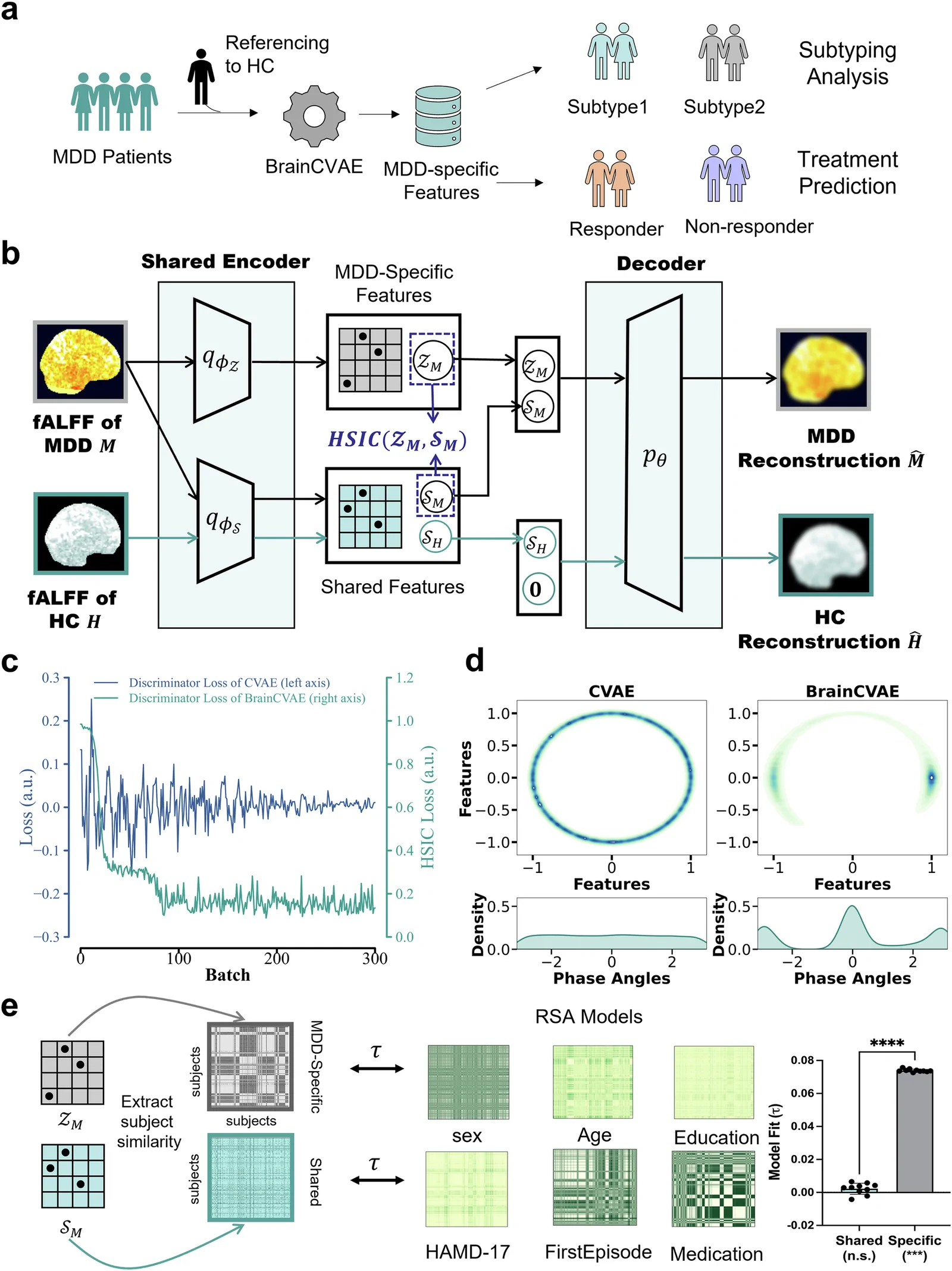
Overview of the participant-level analysis pipeline. **a** We used BrainCVAE framework to obtain MDD-specific features. MDD-specific features were subsequently utilized for subtype analysis and treatment outcome prediction. **b** Architecture for BrainCVAEs. The fALFF of MDD was fed into two sets of encoders ${q}_{{\Phi }_{{\mathscr{Z}}}}$ and ${q}_{{\Phi }_{{\mathscr{S}}}}$, which outputted the MDD-specific features ${Z}_{M}$ and shared features ${S}_{M}$ respectively. While the fALFF of HC was fed into ${q}_{{\Phi }_{{\mathscr{S}}}}$, which outputted shared features ${S}_{H}$. The latent variables ${Z}_{M}$ and ${S}_{M}$ for the MDD were concatenated and fed into a decoder network, while only the (zero-padded) latent variable ${S}_{H}$ was fed into the decoder network. The decoder generated reconstructions of the fALFF of MDD and HC. **c** Comparison of discriminator loss between CVAE and BrainCVAE. The figure illustrates the variation in discriminator loss during training over the first 300 batches. **d** Distribution of MDD-specific features from CVAE and BrainCVAE in ${{\mathbb{R}}}^{2}$. The features were first embedded onto a 2-dimensional unit hypersphere using t-SNE and their distributions were subsequently estimated by Gaussian kernel density estimation (KDE, top row). The density of the phase angles of the feature points on the unit hypersphere was also visualized (bottom row). **e** Representational Similarity Analysis (RSA). MDD-specific ${Z}_{M}$ and shared features ${S}_{M}$ were used to generate a dissimilarity matrix to represent the pairwise dissimilarity between participants. Then the dissimilarity matrix was compared to the dissimilarity matrix for each individual characteristic using the Kendall rank correlation coefficient (Kendall *τ*).

The BrainCVAE model utilized an adapted architecture derived from a recent study^71^. Related work is provided in Supplementary Note 1. The overall framework of BrainCVAE is illustrated in Fig. 6b. Here, the inputs of BrainCVAEs are fALFF rather than functional connectivity measures or structural MRI. The frequency range of fALFF, typically between 0.01 and 0.1 Hz, has been demonstrated to be a consistent indicator of spontaneous neuronal activity at rest^72^ and has a moderate to substantial test-retest reliability^73^. This highlights its validity as a measure for detecting individual differences in regional brain function^74^.

The inputs consisted of fALFF volumes of MDDs $M\in {{\mathbb{R}}}^{64\times 64\times 64}$ and HCs $H\in {{\mathbb{R}}}^{64\times 64\times 64}$, both resampled to a resolution of 64×64×64 in their native space. The BrainCVAE consists of three modules: an encoder ${q}_{{\Phi }_{{\mathscr{Z}}}}$ is used to learn MDD-specific features ${Z}_{M}\in {{\mathbb{R}}}^{32\times 1}$; an encoder ${q}_{{\Phi }_{{\mathscr{S}}}}$ is used to learn shared features ${S}_{M}\in {{\mathbb{R}}}^{32\times 1}$ and ${S}_{H}\in {{\mathbb{R}}}^{32\times 1}$ and a decoder ${p}_{\theta }$ is able to reconstruct fALFF images of MDD $\hat{M}\in {{\mathbb{R}}}^{64\times 64\times 64}$ and HCs $\hat{H}\in {{\mathbb{R}}}^{64\times 64\times 64}$ from the latent 64-dimensional features. Here, ${q}_{{\Phi }_{{\mathscr{Z}}}}$ and ${q}_{{\Phi }_{{\mathscr{S}}}}$ are convolutional layers, while ${p}_{\theta }$ is two deconvolutional layers. For reconstructing fALFF of MDD participants, the latent 64-dimensional representation was a concatenation of the MDD-specific features and shared features (${Z}_{M}$ and ${S}_{M}$). For reconstructing fALFF of HC participants, a concatenation of the shared features (${S}_{H}$) and 32 zeros was fed into ${p}_{\theta }$.

In addition, we forced the independence between MDD-specific features ${Z}_{M}$ and shared features ${S}_{M}$ through minimizing the mutual information between ${Z}_{M}$ and ${S}_{M}$. In general, the estimation of mutual information is commonly done by using the mutual information neural estimator (MINE), which requires the training of an auxiliary neural network f. However, the use of f can lead to instability in the estimation of mutual information between ${Z}_{M}$ and ${S}_{M}$, and in some cases, even result in negative values in the REST-meta-MDD II (Fig. 5c). To address this issue, we used Hilbert-Schmidt independence criterion (HSIC) to calculate the mutual information between ${Z}_{M}$ and ${S}_{M}$. HSIC is defined as the Hilbert-Schmidt norm of the cross-covariance operator between distributions in Reproducing Kernel Hilbert Space (RKHS). This allows us to directly estimate the mutual information between ${Z}_{M}$ and ${S}_{M}$ from data without the need for discrete probability density function (PDF) estimation or any auxiliary neural networks. The formulation of HSIC is:

1 $$HSIC({Z}_{M},{S}_{M})={(m-1)}^{-2}tr({K}_{{Z}_{M}}H{K}_{{S}_{M}}H),$$

where m is number of batch size, ${tr}(\cdot )$ is the trace operator, ${K}_{{Z}_{M}}\in {{\mathbb{R}}}^{m\times m}$ and ${K}_{{S}_{M}}\in {{\mathbb{R}}}^{m\times m}$ are kernel matrices with ${K}_{{Z}_{M}}^{i,j}={k}_{{Z}_{M}}({z}_{M}^{i},{z}_{M}^{j})$ and ${K}_{{S}_{M}}^{i,j}={k}_{{S}_{M}}({s}_{M}^{i},{s}_{M}^{j})$, respectively and $H\in {{\mathbb{R}}}^{m\times m}$ is the centering matrix $H={I}_{m}{l}_{m}{l}_{m}^{T}$. Here, we used Gaussian kernel $k(x,y)\sim \exp (-\frac{1}{2}{\Vert x-y\Vert }^{2}/{\sigma }^{2})$ to estimate kernel matrices. In Fig. 5c, we found that the use of HSIC mutual information significantly improved the stability of training compared to CVAE. In addition, we employed the t-SNE algorithm to visualize feature disentanglement and observed that HSIC effectively separates MDD-specific features from shared features, whereas CVAE fails to achieve such a distinction Fig. 5d.

The objective of our modified BrainCVAE can be defined:

2 $$\begin{array}{ll}{\mathrm {L}}_{\mathrm{CVAE}}={\bf{E}}\left[{\left\Vert M-\hat{M}\right\Vert }_{F}\right]+{\bf{E}}\left[{\left\Vert H-\hat{H}\right\Vert }_{F}\right]+\alpha \cdot \left(\begin{array}{lll}{\bf{E}}\left[{D}_{KL}\left[{q}_{{\varPhi }_{{\mathscr{Z}}}}\left({Z}_{M}|M\right)\parallel {p}_{\theta }\left({Z}_{M}\right)\right]\right]\\ +{\bf{E}}\left[{D}_{KL}\left[{q}_{{\varPhi }_{{\mathscr{S}}}}\left({S}_{M}|M\right)\parallel {p}_{\theta }\left({S}_{M}\right)\right]\right]\\ +{\bf{E}}\left[{D}_{KL}\left[{q}_{{\varPhi }_{{\mathscr{S}}}}\left({S}_{H}|H\right)\parallel {p}_{\theta }\left({S}_{H}\right)\right]\right]\end{array}\right) \\\quad\qquad+\beta \cdot HSIC({Z}_{M},{S}_{M}),\end{array}$$

where ${\mathbb{E}}$ is expectation, ${D}_{{KL}}$ is KL divergence and α and β are hyperparameters.

#### Optimization and implementation details

The BrainCVAE model was implemented using PyTorch^75^, and the Adam optimizer^76^ was used with an initial learning rate of 0.005. The model was trained for 15,000 batches with a batch size of 64. For the REST-meta-MDD I and REST-meta-MDD II datasets, we trained a new model using their respective data. For other datasets, we directly leveraged the BrainCVAE pre-trained on REST-meta-MDD II to extract MDD-specific and shared features without training additional models. Hyper-parameters and parameters of BrainCVAE for the REST-meta-MDD I and REST-meta-MDD II dataset are shown in Supplementary Tables 13–16.

### Representational similarity analysis

To explore the type of information represented in the latent features, we employed representational similarity analysis (RSA) to investigate the potential correlation between MDD-specific features and individual variations in clinical characteristics among MDD participants, such as age, education, first episode, and scores on the HAMD-17 and HAMA. The workflow of the RSA analysis is demonstrated in Fig. 6e. Initially, we calculated the pairwise dissimilarity between participants based on the BrainCVAE functional features (MDD-specific and shared features) to generate a dissimilarity matrix. This process was repeated for each clinical characteristic. For continuous variables such as age and HAMD scores, the subject distance was calculated as the absolute Euclidean distance between their measurements (i.e., the difference in age or HAMD score). As for categorical variables (such as sex or medication status), the subject distance was considered 0 if the categorical variables matched (e.g., same gender or medication status) and 1 otherwise. Finally, the BrainCVAE dissimilarity matrix was compared to the dissimilarity matrix for each individual characteristic using the Kendall rank correlation coefficient (Kendall τ). To ensure the reliability of the RSA analyses on various samples extracted from the BrainCVAE’s latent distributions, we randomly sampled the latent distribution for each MDD participant 10 times, which resulted in 10 different reconstructions of the dataset. RSA models were then fitted to each reconstruction separately. Please note that we deliberately limited the number of repetitions (10 times) to maintain a strict approach in these tests, as larger samples can result in significant effects even with minor differences.

### Cluster analysis

We used K-means clustering method to identify distinct MDD subtypes based on MDD-specific features. To determine the optimal number of clusters, we utilized the elbow plot method. We fitted a K-means clustering model with a range of clusters (1–9) and calculated the distortion as a measure of model fit. Distortion is defined as the average of the squared Euclidean distance from the respective cluster’s centroid^77^. We additionally evaluated the optimal cluster number using the Silhouette Score (difference between mean nearest-cluster and intra-cluster distances, normalized by the maximum of the two), the Variance Ratio Criterion (ratio of between-cluster to within-cluster sum of squares), and the Reverse Davies–Bouldin index (inverse of mean similarity ratio between each cluster and its most similar neighbor), with higher values indicating better clustering quality. Furthermore, the number of iterations is set to 300 and the initial centroid iterations are set to the default number of 10. Figure 1b and Fig. 1h show mean distortion for each number of clusters C in REST-meta-MDD II and REST-meta-MDD I, respectively. The average distortion shows a slower decrease after C = 2. We also compared the clustering performance of HYDRA and SuStaIn, with technical details provided in the supplementary Note 3. The stability of clustering solutions was evaluated using the Adjusted Rand Index (ARI^38^), the Fowlkes–Mallows Index (FMI^36^), and Cohen’s Kappa coefficient (KAP). Specifically, the ARI measures the degree to which pairs of samples are consistently assigned to the same or different clusters, correcting for the agreement expected by chance. The FMI reflects the geometric mean of precision and recall, providing a balanced measure of correctly co-assigned sample pairs. The KAP captures the overall agreement between categorical label, accounting for the proportion of concordance that could occur randomly. For all three indices, values close to 1 indicate strong clustering stability and high reproducibility, whereas values near 0 suggest low or random-level agreement.

### Case–control analysis of fALFF

In order to obtain the functional patterns of each subtype, we conducted group comparisons of fALFF values using Statistical Parametric Mapping (SPM, https://www.fil.ion.ucl.ac.uk/spm/software/spm12/). For each voxel, a general linear model (GLM) was used to investigate the difference in fALFF between each subtype and HC. The GLM analyses included gender, age, and education as covariates, with the primary focus on the effect of group on fALFF. Significance was determined by individual voxel *p* < 0.05 with FDR correction.

### Symptom network analysis

#### Symptom measurement

Symptom-level data were derived from the 17-item Hamilton Depression Rating Scale (HAMD-17), a widely used clinician-rated instrument for assessing depression severity. These items represent the following symptoms: depressed mood, guilty, suicide, insomnia (early), insomnia (middle), insomnia (late), anhedonia, retardation, agitation, anxiety (psychic), somatic anxiety, gastrointestinal, general somatic, genital, hypochondriasis, weight loss and insight. Each item was treated as an individual symptom node in the network, and raw item scores were used for analysis.

#### Network construction

We employed network analysis to investigate differences in network structure between affective and somatic symptoms across the two depression subtypes. Symptom networks were constructed by treating HAMD-17 item scores as nodes and pairwise Pearson correlations between items as edge weights.

To obtain sparse and interpretable network structures, a Gaussian graphical model was estimated using the graphical least absolute shrinkage and selection operator (LASSO), implemented with the Extended Bayesian Information Criterion (EBIC). The tuning parameter (γ) was set to 0.5 to balance model parsimony and sensitivity. This regularization approach shrinks weak associations to zero, retaining only the most robust connections between symptoms.

#### Centrality analysis

Network centrality indices were calculated to quantify the importance of each symptom node. Strength represents the sum of absolute edge weights connected to a node, indicating overall connectivity; closeness is defined as the inverse of the average shortest path length between a node and all other nodes, reflecting how directly a symptom is connected to others; and betweenness quantifies the frequency with which a node lies on the shortest paths between other nodes, capturing its role as a bridge within the network.

#### Implementation

All analyses were performed using the R packages bootnet and qgraph.

### Predictive modeling of treatment responses using machine learning approaches

Deep learning (DL) and machine learning (ML) techniques were employed to differentiate between patients who responded positively to medication treatment and those who did not. In this study, the REST-meta-MDD II dataset included 72 responders versus 72 non-responders for all medications, and 63 responders versus 63 non-responders for SSRI medications. We compared the performance of medication classification using common DL/ML methods. To address overfitting to noise and prevent inflated performance, ML methods first applied Principal Component Analysis (PCA) for dimensionality reduction of MDD-specific features (Z) with 512 dimensions, followed by classification of the corresponding labels (Y) using XGBoost, LightGBM, Logistic Regression, and Gradient Boosting. For DL methods, MDD-specific features (Z) were extracted and Z-score normalization was applied to eliminate dimensional discrepancies. A two-layer multi-layer perceptron was used for classification, with the input layer matching the dimensionality of Z, a hidden layer containing 256 neurons (ReLU activation), and an output layer with a single neuron (Sigmoid activation) for binary classification probability prediction. During training, the Adam optimizer (learning rate = 1e-3) was used to minimize binary cross-entropy loss over 50 epochs. Accuracy, area under the curve (AUC), F1 score, and recall were used as evaluation metrics. Furthermore, PCA and ML methods, including Light GBM, XGBoost, Random Forest, Support Vector Regression, Gradient Boosting, Ridge Regression, and Lasso Regression, were employed to predict clinical improvements in HAMD-17 scores after rTMS treatment. Clinical improvements were quantified by subtracting post-treatment scores from pre-treatment scores. All methods were implemented in Python using the scikit-learn package.

Evaluation of both classification and regression experiments was conducted using nested cross-validation, consisting of two nested cross-validation loops (Supplementary Fig. 16). The outer loop assesses the model’s generalization performance, with the dataset being split into 5 folds, where one subset is selected as the test set for each iteration to report the prediction results. The inner loop further divides the training set into 3 folds, where grid search is used to evaluate model performance across different hyperparameter combinations, outputting the optimal hyperparameter set. Details regarding the selection of hyperparameters for the DL and ML methods can be found in Supplementary Table 17, 18 and Fig. 15.

### Statistics

Linear mixed models (LMM) were employed to compare the clinical variables (age, education, illness duration, HAMD-17 total scores, each item of HAMD-17) and rTMS treatment outcomes (differences in HAMD-17 after undergoing rTMS treatment for 5 days) between distinct subtypes. The model was defined as y ∼1 + Diagnosis + Age + Sex + Education + Motion + (1|Site) + (Diagnosis|Site). This model provided *t* and *p* values for the fixed effect of Diagnosis. Cohen’s d effect size was also calculated as $d=\,\frac{T({n}_{1}+{n}_{2})}{\sqrt{{df}}\sqrt{{n}_{1}{n}_{2}}}$^78^. The diagnosis variable of LMM was defined as the subtype status. In addition, chi-square tests were used to compare the response rate, treatment-responsive rate, sex and family history between different subtypes. Multiple comparisons were controlled using the Benjamini–Hochberg false discovery rate (FDR) procedure, applied across all tests within each family of comparisons, with a significance threshold of $p\, < \,0.05$.

To compare the performance differences between our method and HYDRA and SuStaIn, we performed 100 bootstrap iterations for each method, with each iteration including 80% of the samples, and calculated the consistency index (FMI, ARI, KAP) for each iteration. A repeated measures one-way analysis of variance (ANOVA) was conducted to examine whether there were significant differences in the consistency indices among the three methods. Since Mauchly’s test of sphericity indicated a violation of the sphericity assumption, the Greenhouse-Geisser correction was applied to adjust the degrees of freedom. The significance level was set at 0.05.

To determine the statistical significance of the machine learning-based treatment prediction results, a nonparametric permutation test was employed. In the medication task, we randomly shuffle the labels of medication treatment response across participants 1000 times, then repeat the nested cross-validated classification procedure to obtain an accuracy distribution. The *p* value is defined as the proportion of cross-validated accuracy that is higher than those without permutation. Similarly, in the rTMS task, we randomly shuffle the observed HAMD-17 score changes across participants 1000 times, then repeat the cross-validated classification procedure to obtain a distribution of Pearson’s correlation coefficients. The *p* value is defined as the proportion of correlation coefficients that are higher than those without permutation.

## Supplementary information

Supplementary

## Data Availability

Deidentified and anonymized data were contributed from studies approved by local Institutional Review Boards. All study subjects provided written informed consent at their local institution. Data of REST-meta-MDD I are available at: http://rfmri.org/REST-meta-MDD. Data of REST-meta-MDD II, Xiangya, XJ-KG, XJ-QG and SAINT used during the current study are available from the corresponding author upon reasonable request.

## Code availability

Codes used during the current study are available at GitHub repository (https://github.com/ZKZ-Brain/Contrastive-Learning-for-MDD-subtyping).
