# Supplementary material for: Brain contrastive modeling reveals depression subtypes with distinct treatment response and progression
Source: NPJ Digit Med. 2026 Jul 20;9:883. doi: 10.1038/s41746-026-03011-8 (PMC13752283; doi:10.1038/s41746-026-03011-8)
Supplement: Supplementary file 1 [file 41746_2026_3011_MOESM1_ESM.pdf]

Supplementary Materials

Supplementary Note 1 Related Work.

Contrastive learning is a generic procedure in unsupervised or self-supervised learning, where the main idea is to compare (semantically) similar (positive) and dissimilar (negative) data pairs, encouraging the representations of similar pairs to be close, and those of dissimilar pairs to be more orthogonal [1, 2]. Recently, this concept has been applied in the field of neuroimaging to study the underlying neuroanatomical variations within autism [3]. The authors utilized contrastive variational autoencoders (CVAEs) [4, 5] to separate neuroanatomical variations specific to autism spectrum disorder (ASD). Unlike other unsupervised learning methods, this approach also allows for the inclusion of a healthy control group as a reference, enabling the separation of brain features into disease-specific and shared features. This prevents clustering based on disease-irrelevant features. In this study, we employed contrastive learning to obtain MDD-specific features, which can capture the underlying nature of MDD, without being affected by confounding variables. The MDD subtypes identified through the use of MDD-specific features are reliable, providing a strong foundation for establishing a link between subtypes and treatment outcomes.

Supplementary Note 2 Dataset Descriptions.

REST-meta-MDD Dataset

Datasets. The REST-meta-MDD datasets (REST-meta-MDD I and II) were provided by the DIRECT consortium [6], established in 2017. The consortium's objective is to aggregate neuroimaging data from multiple independent sites, thereby enhancing the

statistical power of data analyses and promoting the clinical application of neuroimaging study findings. The fMRI data for REST-meta-MDD I were collected from 24 hospitals or centers across China, while the fMRI data for REST-meta-MDD II were obtained from 23 hospitals or centers in China. All participants in both datasets were of Chinese descent. Supplementary Table 8 and 9 show the site information of REST-meta-MDD II and I, respectively. Supplementary Table 10 and 11 demonstrate the image acquisition of REST-meta-MDD II and I, respectively.

Exclusion criteria. Participants who (i) had incomplete necessary demographical information (i.e., age, sex, education, and head motion); (ii) had bad image coverage (< 90 % of the group mask) or excessive head motion (mean FD > 0.2mm) were excluded from the study. The final sample comprised 1590 MDD patients and 1308 healthy controls (HCs) in the REST-meta-MDD II dataset, and 1248 MDD patients and 1088 HCs in the REST-meta-MDD I dataset.

Xiangya Dataset

Inclusion Criteria: Participants included in the study were (i) aged 18 to 55 years; (ii) education ≥ 6 years; (iii) right-handed. For the MDD group; (iv) diagnosed with MDD with a current major depressive episode by an attending psychiatrist using Structured Clinical Interview for DSM-IV (SCID-IV); (v) currently with at least moderate depression severity, indicated by a 24-item Hamilton Depression Rating Scale (HAM-D_{24}) score ≥ 20; (vi) no psychotropic medication for at least 2 weeks (6 weeks for fluoxetine) before inclusion.

Exclusion criteria: Participants who (i) had incomplete necessary demographical

information (i.e., age, sex, education, and head motion); (ii) had bad image coverage (< 90 % of the group mask) or excessive head motion (mean FD > 0.2mm) were excluded from the study. (iii) did not undergo follow-up; (iv) did not undergo scale assessments (e.g., HAMD-17) during the follow-up period.

XJ-KG Dataset

Trial registration. The dataset was approved by the Ethics Committee of the First Affiliated Hospital, Fourth Military Medical University, and was registered with clinicaltrial.gov (identifier: NCT 05544071).

Inclusion Criteria: Participants included in the study (i) met the DSM-5 diagnostic criteria for Major Depressive Disorder (MDD) and were in an active episode; (ii) had a total score greater than 17 on the 17-item Hamilton Depression Rating Scale (HAMD-17) at both screening and baseline; (iii) had a total score greater than 20 on the Snaith-Hamilton Pleasure Scale (SHAPS) at both screening and baseline; and (iv) had not used any antidepressant medication for at least two weeks prior to screening.

Exclusion criteria: Participants who (i) had substance use disorders other than nicotine or caffeine; (ii) had current psychiatric disorders other than dysthymic disorder, generalized anxiety disorder, social anxiety disorder, panic disorder, agoraphobia, or specific phobias (including those with unstable conditions or those who had focused treatment on these conditions in the past 6 months); (iii) had a history of schizophrenia or schizoaffective disorder, or current or past depressive episodes with psychotic features; (iv) had other psychiatric disorders, personality disorders, or intellectual disabilities primarily presenting with depressive symptoms; (v) had significant clinical

abnormalities detected during screening that could impact study participation or safety, or interfere with the interpretation of study results; (vi) had any medical history that, in the investigator's judgment, could pose a risk to the participant or interfere with the interpretation of study results; (vii) were concurrently participating or had participated within the last month in other drug or device clinical trials; (viii) had metal or electronic devices in the head or skull; (ix) had a history of epilepsy; (x) had a history of cardiovascular disease or cardiovascular events; (xi) had a history of obsessive-compulsive disorder; (xii) had a history of autism spectrum disorder; (xiii) had prior exposure to rTMS; or (xiv) had any other conditions deemed inappropriate by the investigator for study participation were excluded from the study.

XJ-QG Dataset

This dataset was based on a clinical randomized double-blind trial, approved by the Ethics Committee of Xijing Hospital, Fourth Military Medical University, and conducted in accordance with the Declaration of Helsinki.

Inclusion Criteria: Participants who (i) were aged between 18 and 60 years and right-handed; (ii) met the DSM-V criteria for major depressive disorder (MDD); (iii) had a HAMD-17 total score greater than 17; (iv) had a BSI-CV score of 6 or higher; (v) showed normal results on physical examination, medical history, vital signs, routine blood, urine, and stool tests, liver and kidney function, electrolytes, Electrocardiogram (ECG), and Electroencephalogram (EEG); (vi) were willing to actively cooperate with treatment after fully understanding the safety of rTMS and signed the informed consent form.

Exclusion criteria: Participants who (i) were under 18 years of age or over 60; (ii) had a history of severe physical illness or depression induced by other psychoactive or non-addictive substances; (iii) had metal or electronic implants in their body, such as intracranial metal objects, cochlear implants, pacemakers, or stents; (iv) were at risk of seizures, had a history of cranial disease, head trauma, alcohol abuse, abnormal EEG, MRI evidence of brain structural abnormalities, or a family history of epilepsy; (v) had engaged in suicidal or serious self-harm behaviors requiring urgent intervention; (vi) had psychotic symptoms requiring the use of antipsychotic medication; (vii) had received Electroconvulsive Therapy (ECT) treatment within 2 months prior to enrollment; or (viii) were pregnant, breastfeeding, or planning to become pregnant during the trial.

SAINT Dataset

Trial registration. The dataset was approved by the Ethics Committee of the First Affiliated Hospital, Fourth Military Medical University, and was registered with clinicaltrial.gov (identifier: NCT 04653337).

Treatment. Each treatment was administered using a Black Dolphin Navigation Robot system (SmarPhin S-50, Solide Brain Control Medical Technology Co., Ltd., Xi'an, China). In a precise experimental protocol, three consecutive sessions of intermittent theta-burst stimulation (iTBS) were delivered at 90% of the resting motor threshold (RMT) within a 9 minute and 52 second interval. This regimen was repeated for a total of ten iTBS sessions, with a 50-minute interval between each session. Patients received this treatment daily for five consecutive days, resulting in a cumulative total of 90,000

pulses throughout the entire duration of treatment. For ethical and safety considerations, venlafaxine (75mg/d) or duloxetine (30mg/d) were prescribed at the start of treatment. Additionally, dexzopiclone or zolpidem was administered to improve sleep quality for individuals with severe insomnia. Supplementary Tab. S12 demonstrates MNI coordinates of the stimulation targets and the corresponding superficial depths for all 26 patients.

Inclusion Criteria: Participants included in the study were (i) between 18 and 60 years old; (ii) diagnosed with unipolar MDD according to the criteria of the Diagnostic and Statistical Manual of Mental Disorders, Fifth Edition (DSM-5) as assessed by the Mini-Neuropsychiatric Interview (MINI); (iii) right-handed; (iv) with a score > 17 on the HAMD-17; (v) with a score ≥ 6 on the Beck Scale for Suicidal Ideation-Chinese Version (BSI-CV); (vi) showed normal results on physical examination and electroencephalography.

Exclusion criteria: Participants who (i) had received antidepressant treatment within two months prior to the study; (ii) had any other current or past psychiatric axis-I or axis-II disorders; (iii) had severe physical illnesses; (iv) experienced psychotic symptoms, alcohol or drug abuse; (v) had a history of neurological disorders including seizures, cerebral trauma, or MRI evidence of structural brain abnormalities; (vi) had contraindications to MRI and rTMS, such as metallic implants in the body, cardiac pacemakers, or claustrophobia; (vii) were experiencing acute suicidal or self-injury behavior requiring immediate intervention; (viii) were pregnant, lactating, or planning a pregnancy, were excluded from the study.

Image Acquisition: The parameters for resting-state fMRI with eyes closed were as follows: slices = 35, repetition time = 2000 ms, echo time = 30 ms, slice thickness = 4 mm, matrix size = 64 × 64, field of view = 224 × 224 mm^2, and flip angle = 90°.

Supplementary Note 3 Supplementary Results

Symptom Network Analysis Results

Network analysis revealed distinct patterns in betweenness centrality between subtypes. In subtype 1, the majority of affective symptoms (including depressed mood, guilt, and psychic anxiety) exhibited higher betweenness centrality than somatic symptoms, whereas subtype 2 demonstrated the opposite pattern, with somatic symptoms (such as retardation, insomnia, and somatic anxiety) showing substantially greater betweenness, suggesting they serve as key bridge symptoms connecting different symptom domains within the network. To systematically compare network properties between subtypes, betweenness centrality differences were visualized using a dumbbell plot, where each symptom is represented by paired markers (circles for subtype 1, triangles for subtype 2) connected by lines to facilitate direct comparison of the relative importance of each symptom as a network bridge.

HYDRA and SuStaIn Cluster Results

Similar to the method proposed in this study, the Heterogeneity through Discriminative Analysis (HYDRA) is a nonlinear semi-supervised machine learning clustering method that constructs a convex polytope classifier by maximizing the boundary between the healthy control group and the disease group, thereby reducing clustering errors caused by irrelevant features. HYDRA identifies the optimal number

of clusters by maximizing the Adjusted Rand Index (ARI). We applied the HYDRA method to replicate the clustering and pattern analysis process on the REST-MDD-II dataset. The mean fALFF values of 116 ROIs from the AAL template were used as model inputs, and the influence of covariates (age, sex, education level) was removed. During model inference, the settings were as follows: maximum number of clusters = 3, regularization parameter = 0.25, number of iterations = 50, number of cross-validation folds = 10, and consensus steps = 10, with all other parameters set to default values.

We also included the clustering and staging results from Subtype and Stage Inference (SuStaIn). SuStaIn is a data-driven, unsupervised learning algorithm designed to identify individual subtypes with different biomarker progression patterns from cross-sectional data. We applied the linear z-score model reported by Young et al. (2018) to infer the clustering and staging results on the REST-MDD-II dataset. Specifically, we calculated the mean fALFF values of 116 ROIs from the AAL template, removed the influence of covariates (age, sex, education level), and compared the average fALFF values in these brain regions between MDD patients and healthy controls (HC) using a two-tailed, two-sample t-test. The comparative analysis identified 15 brain regions with significant differences ($p < 0.05$ after FDR correction). However, due to the time complexity of the SuStaIn algorithm being proportional to the fourth power of the input feature dimensions, we selected the 10 ROIs with the largest *p*-values as inputs for the SuStaIn model and performed regression to control for confounding variables (age, sex, education level). Subsequently, we calculated the *Z*-score values for

each of the 10 features for each MDD patient, as shown in the following formula:

$$Z(i) = \frac{ROI(i) - \mu_{HC}(i)}{\sigma_{HC}(i)}$$

Where $i \in [1,10]$, $ROI(i)$ represents the average fALFF value of the i-th ROI, $\mu_{HC}(i)$ and $\sigma_{HC}(i)$ represent the mean and standard deviation of the fALFF values for the HC group. Finally, we defined the staging results of the SuStaIn model based on Z-Score value severity thresholds [1, 2, 3]. The model inference process was repeated through 5 random starting points, 10-fold cross-validation, and 1000 iterations. The maximum number of clusters was set to 3, and the optimal number of clusters was determined by the out-of-sample log-likelihood value.

The HYDRA method also yielded the highest ARI (0.6696) when the number of clusters was set to 2, classifying 691 MDD patients into Subtype 1 and 899 MDD patients into Subtype 2. The difference in illness duration between the two groups was significant (Subtype 1: 30.84 ± 2870.89, Subtype 2: 46.40 ± 4592.51, $t = 5.113$, $p < 0.001$). Additionally, we replicated the voxel-level comparisons of the two subtypes after FDR correction, as reported in the original study.

The SuStaIn algorithm divided the MDD patients into two subtypes and 30 potential stages. Subtype 1 consisted of 1085 patients, while Subtype 2 had 505 patients. The difference in illness duration between the two groups was significant (Subtype 1: 30.99 ± 2780.94, Subtype 2: 45.47 ± 4519.88, $t = 4.268$, $p < 0.001$). Furthermore, although we did not observe a correlation between the SuStaIn model's staging results and illness duration ($r = -0.1458$), we noted that Stage 0 was predominantly distributed in the HC group and Subtype 1 (45.19% in HC, 30.14% in Subtype 1, 24.67% in

Subtype 2), while Stages 1–30 were primarily distributed in Subtype 2 (1.02% in Subtype 1, 54.41% in Subtype 2, $\chi^2 = 663.55$, $p < 0.0001$). This suggests a strong association between Subtype 2 and the disease progression stages.

Table Legends

Supplementary Table 1 A summary of MDD subtyping studies based on MRI.

Study	Sample Size (MDD)	Subtyping based on	Method	Number of subtypes	Subtype descriptions
Tozzi et al.[7], 2024	801	Task-free and task-evoked fMRI	Hierarchical clustering	6	Six biotypes defined by distinct profiles of intrinsic task-free functional connectivity within the default mode, salience and frontoparietal attention circuits, and of activation and connectivity within frontal and subcortical regions elicited by emotional and cognitive tasks
Cynthia et al.[8], 2024	685	T1-weighted MRI image	HYDRA	2	1. preserved gray and white matter<br>2. widespread subtle reductions in gray and white matter
Sun et al.[9], 2023	1148	Functional connectivity	normative model	2	1. positive deviations in the default mode, limbic, and subcortical areas and negative deviations in the sensorimotor and attention areas.<br>2. a moderate but converse deviation pattern.
Wen et al.[10], 2022	501	T1-weighted MRI image	HYDRA	2	1. preserved brain anatomy without WM disruptions<br>2. widespread brain atrophy and WM integrity disruptions
Zheng et al.[11], 2022	848	Functional connectivity	Graph neural networks and clustering by fast search and find of density peaks (CFDP)	3	1. subcortical-limbic,<br>2. DMN-FPN<br>3. VN-SMN
Wang et al.[12], 2021	115	Functional connectivity	Regularized CCA and K-means	2	1. hyperconnectivity within the ventral attention network<br>2. hypoconnectivity in the subcortical

					and dorsal attention networks
Liang et al.[13], 2020	690	Functional connectivity	K-means and PCA	2	1. increase DMN connectivity<br>2. decrease DMN connectivity
Drysdale et al.[14], 2017	220	Functional connectivity and HAMD-17	Hierarchical clustering	4	1. Reduced connectivity in frontoamygdala<br>2. Low anhedonia-related connectivity<br>3. Hyperconnectivity in thalamic and frontostriatal networks<br>4. Large, reduced connectivity in frontoamygdala, hyperconnectivity in thalamic and frontostriatal networks
Feder et al.[15], 2017	159	Functional connectivity	Hierarchical clustering and K-means	2	1. Higher connectivity<br>2. Lower connectivity
Price et al.[16], 2017	68	Functional connectivity	Group Iterative Multiple Model Estimation	2	1. Fewer specific connections.<br>2. greater overall connectivity
Price et al.[17], 2017	80	Effective connectivity	Group Iterative Multiple Model Estimation	2	1. Directed pathway linking two DMN hubs<br>2. Lacking within-DMN connectivity
Cheng et al.[18], 2014	61	Fractional anisotropy	Classification analysis	2	1. Stronger WM connectivity<br>2. WM microstructural deficits

Supplementary Table 2 Demographic and clinical variables for MDD and healthy control participants on the REST-meta-MDD II.

	Healthy controls	MDD participants	MDD subtype1	MDD subtype2
**Sample size**	1308	1590	802	788
**Age (yr)**	32.4	32.9	28.2	37.8
**Age range (yr)**	9-67	11-77	11-71	12-77
**Sex**				
**Male (number, percentage)**	562(42.9%)	578 (36.3%)	268 (33.4%)	310 (39.3%)
**Female (number, Percentage)**	746(57.0%)	1012 (63.6%)	534 (66.5%)	478 (60.6%)
**Years of education**	13.7 (12.9)	12.0 (13.9)	12.5 (12.5)	11.5 (14.9)
**Education range (yr)**	0-25	0-26	0-26	0-21

**HAMD-17**	——	22.6 (65.4）	22.0 (55.3)	23.2 (74.5)
**HAMA**	——	18.4 (73.9)	17.6 (65.3)	19.1 (80.9)
**Illness Duration (mo)**	——	41.2 (4048.0)	24.8 (1827.0)	55.2 (5518.3)
**Illness Duration range (mo)**	——	0-480	0-360	0-480
**First episode/recurrent MDD**	——	995/440	575/134	420/306
**Family History (number)**	——	93	36	57
**Treatment Responsive**	——	160	58	102

AAD = Atypical Antidepressant, Family History = Number of individuals with family history, Treatment Responsive = Number of patients with non-resistant treatment.

Supplementary Table 3 Demographic and clinical variables for MDD and normal control participants on the REST-meta-MDD I.

	Healthy controls	MDD participants	MDD subtype1	MDD subtype2
**Sample size**	1088	1248	559	689
**Age (yr)**	36.0	36.2	37.9	34.9
**Age range (yr)**	14-80	12-82	12-82	12-77
**Sex**				
**Male (number, percentage)**	456 (41.8%)	453 (36.3%)	213 (38.2%)	240 (35.1%)
**Female (number, Percentage)**	632 (58.2%)	795 (63.7%)	346 (61.8%)	449 (64.9%)
**Years of education**	12.3 (24.8)	11.3 (17.5)	11.4 (16.5)	11.2 (18.4)
**Education range (yr)**	0-23	3-21	0-20	0-21
**HAMD-17**	——	20.5 (58.6)	19.8 (68.1)	21.1 (50.4)
**HAMA**	——	19.0 (81.2)	17.2 (68.0)	20.0 (86.9)
**Illness Duration (mo)**	——	36.0 (3583.7)	30.1 (2701.6)	40.3 (4171.6)
**Illness Duration range (mo)**	——	0-480	0-360	0-480
**First episode/recurrent MDD**	——	527/269	254/95	273/174
**Medication (on/off)**	——	398/437	179/235	219/202

Supplementary Table 4 Demographic and clinical variables for MDD participants on the Xiangya.

	MDD participants	MDD subtype1	MDD subtype2
**Sample size**	60	25	35
**Age (yr)**	35.6	37.4	34.3
**Age range (yr)**	18-54	22-51	18-54
**Sex**			
**Male (number, percentage)**	24 (40.7%)	9 (36%)	15 (42.8%)
**Female (number, Percentage)**	36 (59.3%)	16 (64%)	20 (57.1%)
**Years of education**	10.3 (11.9)	10.6 (11.8)	10.1 (12.2)
**Education range (yr)**	5-17	5-16	6-17
**Baseline**			
**HAMD-17**	32.3 (62.0)	33.5 (82.1)	31.5 (47.9)
**HAMA**	18.7 (50.3)	19.2 (59.7)	18.3 (44.9)
**Illness Duration (mo)**	44.8 (3359.2)	58.4 (4724.3)	35.1 (2262.5)
**Illness Duration range (mo)**	1-243	3-243	1-170

Supplementary Table 5 Demographic and clinical variables for MDD participants on the XJ-KG.

	MDD participants	MDD subtype1	MDD subtype2
**Sample size**	73	30	43
**Age (yr)**	14.8	14.8	14.9
**Age range (yr)**	13-17	13-17	13-17
**Sex**			
**Male (number, percentage)**	23 (31.5%)	11 (36.6%)	12 (27.9%)
**Female (number, Percentage)**	50 (68.5%)	19 (63.4%)	31 (72.1%)
**Baseline**			
**HAMD-17**	21.8 (30.5)	21.2 (37.5)	22.3 (25.9)
**Two weeks after treatment**			
**HAMD-17**	14.7 (62.2)	11.6 (43.2)	17.0 (64.6)

Supplementary Table 6 Demographic and clinical variables for MDD participants on the XJ-QG.

	MDD participants	MDD subtype1	MDD subtype2
**Sample size**	44	24	20
**Age (yr)**	36.9	40.8	32.2
**Age range (yr)**	18-61	21-59	18-61
**Sex**			
**Male (number, percentage)**	11 (25.0%)	7 (29.1%)	4 (20.0%)
**Female (number, Percentage)**	33 (75.0%)	17 (70.9%)	16 (80.0%)
**Baseline**			
**HAMD-17**	16.7 (30.3)	18.3 (28.6)	14.7 (26.6)
**Two weeks after treatment**			
**HAMD-17**	10.5 (40.9)	9.0 (33.1)	12.4 (46.0)

Supplementary Table 7 Demographic and clinical variables for MDD participants on the SAINT.

	MDD participants	MDD subtype1	MDD subtype2
**Sample size**	26	15	11
**Age (yr)**	27.8	27.1	29
**Age range (yr)**	18-54	18-54	18-54
**Sex**			
**Male (number, percentage)**	6 (23.0%)	4 (26.7%)	2 (18.1%)
**Female (number, Percentage)**	20 (77.0%)	11 (73.3%)	9 (81.8%)
**Years of education**	12.2 (7.1)	12.5 (8.6)	11.8 (6.8)
**Education range (yr)**	6-16	9-16	6-16
**Baseline**			
**HAMD-17**	27.9 (17.0)	28.9 (17.1)	26.6 (15.3)
**MADRS**	36.6 (23.6)	37.4 (18.8)	35.5 (30.2)

**BDI-II**	36.0 (82.0)	37.6 (119.5)	33.9 (29.1)
**5 days after treatment**			
**HAMD-17**	9.4 (31.8)	8.4 (24.7)	10.7 (41.6)
**MADRS**	14.7(57.1)	13.2 (42.9)	16.6 (75.3)
**BDI-II**	21.3 (128.6)	19.7 (151.5)	23.6 (99.5)
**15 days after treatment**			
**HAMD-17**	8.2 (24.3)	7.8 (16.5)	8.8 (37.0)
**MADRS**	11.7 (37.9)	11.5 (25.7)	11.9 (58.9)
**BDI-II**	20.2 (124.9)	19.5 (147.4)	21.2 (104.2)
**30 days after treatment**			
**HAMD-17**	6.3 (20.8)	6.1 (18.3)	6.5 (26.5)
**MADRS**	9.5 (34.0)	8.9 (32.9)	10.2 (38.0)
**BDI-II**	15.4 (67.9)	14.3 (82.1)	17.0 (50.0)

Supplementary Table 8 Site information of REST-meta-MDD II.

Serial Number	Sites (cohorts)	Principal investigators	Data organizer	Number	
				MDD	HC
1	Faculty of Psychology, Southwest University	Jiang Qiu	Xin-Ran Wu	279	247
2	Department of Psychiatry, The First Affiliated Hospital of Chongqing Medical University	Li Kuang	Qian Huang	135	62
3	National Clinical Research Center for Mental Disorders (Peking University Sixth Hospital) & Key Laboratory of Mental Health, Ministry of Health (Peking University)	Tian-Mei Si	Yan-Kun Wu	20	20
4	Mental Health Center, West China Hospital, Sichuan University	Tao Li	Hua Yu	31	27
5	First Hospital of Shanxi Medical University	Ke-Rang Zhang	Ai-Xia Zhang	128	57
6	The Institute of Mental Health, Second Xiangya Hospital of Central South University	Zhe-Ning Liu	Yi-Cheng Long	59	102
7	Affiliated Brain Hospital of Guangzhou Medical University	Li-Ping Cao	Jian-Shan Chen	21	30
8	The First Affiliated Hospital of Xi'an Jiaotong University, Xi'an Central Hospital	Jian Yang / Xiao-Ping Wu	Peng-Feng Sun	43	42
9	Department of Psychosomatics and Psychiatry, Zhongda Hospital, School of Medicine, Southeast University	Yong-Gui Yuan	Xiao-Yun Liu	47	25
10	Beijing Anding Hospital, Capital Medical University	Chuan-Yue Wang	Feng Li	80	67
11	Department of Neurology, Affiliated ZhongDa Hospital of Southeast University	Chun-Ming Xie	Can-Can He	114	68
12	The Second Xiangya Hospital of Central South University	Guang-Rong Xie	Xi-Long Cui	62	32
13	Department of Clinical Psychology, Suzhou Psychiatric Hospital, The Affiliated Guangji Hospital of Soochow University	Yan-Song Liu	Feng-Nan Jia	32	29
14	The First Affiliated Hospital of Anhui Medical University	Yong-Qiang Yu	Jia-Jia Zhu	136	145
15	Xiangya 2nd Hospital, Central South University	Xiang Wang	Xin-Lei Ji	34	35
16	The First Affiliated Hospital of Jinan University	Ying Wang	Guan-Mao Chen	83	81
17	First Affiliated Hospital of Kunming Medical University	Xiu-Feng Xu / Yu-Qi Cheng	Zhao-Song Chu	44	49
18	Huaxi MR Research Center, West China Hospital of Sichuan University	Qi-Yong Gong	Tao-Lin Chen	33	32
19	The Second Xiangya Hospital of Central South University	Wen-Bin Guo/Jing-Ping Zhao	Xiao-Xiao Shan	32	32
20	The First Affiliated Hospital, College of Medicine, Zhejiang University	Yang Hong	Tao Chen	20	20
21	Xijing Hospital	Hua-Ning Wang/Bao-Juan Li	Bao-Juan Li	62	56
22	West China Hospital, Sichuan University	Wei Zhang	Min-Lan Yuan	35	24
23	Shenzhen Mental Health Center, Shenzhen Kangning Hospital	Jian-ping Lu	Zhen-Peng Xue	60	26

Supplementary Table 9 Site information of REST-meta-MDD I.

Serial Number	Sites (cohorts)	Principal investigators	Data organizer	Number	
				MDD	HC
1	National Clinical Research Center for Mental Disorders (Peking University Sixth Hospital) & Key Laboratory of Mental Health, Ministry of Health (Peking University)	Tian-Mei Si	Tian-Mei Si	74	73
2	Department of Clinical Psychology, Suzhou Suzhou Psychiatric Hospital, The Affiliated Guangji Hospital of Soochow University	Yan-Song Liu	Yan-Song Liu	29	30
3	The Second Xiangya Hospital of Central South University	Shu-Qiao Yao / Xiang Wang	Chang Cheng	25	37
4	Department of Psychiatry, Shanghai Jiao Tong University School of Medicine	Yi-Ru Fang / Dai-Hui Peng	Ru-Bai Zhou	13	11
5	Department of Psychiatry, Shanghai Jiao Tong University School of Medicine	Yi-Ru Fang / Jun-Juan Zhu	Ru-Bai Zhou	14	15
6	Sir Run Run Shaw Hospital, Zhejiang University School of Medicine	Wei Chen	Jia-Shu Yao	35	48
7	Department of Psychiatry, First Affiliated Hospital, China Medical University	Fei Wang	Jia Duan	75	74
8	The First Affiliated Hospital of Jinan University	Ying Wang	Guan-Mao Chen	48	49
9	First Hospital of Shanxi Medical University	Ke-Rang Zhang	Ai-Xia Zhang	50	32
10	Department of Psychiatry, The First Affiliated Hospital of Chongqing Medical University	Qing-Hua Luo / Hua-Qing Meng	Hai-Tang Qiu	31	29
11	Department of Psychiatry, The First Affiliated Hospital of Chongqing Medical University	Hua-Qing Meng / Qing-Hua Luo	Hai-Tang Qiu	32	6
12	The First Affiliated Hospital of Xi'an Jiaotong University, Xi'an Central Hospital	Jian Yang / Xiao-Ping Wu	Hong Zhang	24	15
13	The Second Xiangya Hospital of Central South University	Guang-Rong Xie	Xi-Long Cui	64	32
14	Department of Psychosomatics and Psychiatry, Zhongda Hospital, School of Medicine, Southeast	Yong-Gui Yuan	Zheng-Hua Hou / Ying-	50	50

	University		ying Yin		
15	Huaxi MR Research Center, West China Hospital of Sichuan University	Qi-Yong Gong / Kai-Ming Li	Kai-Ming Li	31	31
16	Department of Psychiatry, The First Affiliated Hospital of Chongqing Medical University	Li Kuang	Lan Hu	47	44
17	Department of Radiology, The First Affiliated Hospital, College of Medicine, Zhejiang University	Hong Yang	Yu-Shu Shi / Hai-Yan Xie	21	20
18	Anhui Medical University	Kai Wang	Tong-Jian Bai	51	36
19	Faculty of Psychology, Southwest University	Jiang Qiu	Xin-Ran Wu	270	244
20	Beijing Anding Hospital, Capital Medical University	Chuan-Yue Wang	Qi-Jing Bo / Feng Li	81	68
21	The Institute of Mental Health, Second Xiangya Hospital of Central South University	Zhe-Ning Liu	Yi-Cheng Long	30	20
22	Mental Health Center, West China Hospital, Sichuan University	Tao Li	Yi-Ting Zhou	32	30
23	First Affiliated Hospital of Kunming Medical University	Xiu-Feng Xu / Yu-Qi Cheng	Chao-Jie Zou	32	31
24	Department of Neurology, Affiliated ZhongDa Hospital of Southeast University	Zhi-Jun Zhang	Zhi-Jun Zhang	89	63

Supplementary Table 10 Image acquisition by site in the REST-meta-MDD II.

Serial Number	Scanner	Receive coil	TR (ms)	TE (ms)	Flip Angle (∘)	Thickness/gap (mm)	Slice number	Time points	Voxel size (mm)	FOV
1	Siemens Tim Trio 3T	12-channel	2000	30	90	3/1	32	242	3.44 × 3.44 × 4.00	220 × 220
2	GE Signa 3T	8-channel	2000	40	90	4/0	33	240	3.75 × 3.75 × 4.00	240 × 240
3	Siemens Tim Trio 3T	32-channel	2000	30	90	4.0/0.8	30	210	3.28 × 3.28 × 4.80	210 × 210
4	Philips Achieva 3T TX	8-channal	2000	30	90	4.0/0	38	240	3.75 × 3.75 × 4.00	240 × 240
5	Siemens Tim Trio 3T	32-channel	2000	30	90	3.00/1.52	32	212	3.75 × 3.75 × 4.52	240 × 240
6	Philips Gyroscan Achieva 3T	32-channel	2000	30	90	4/0	36	250	1.67 × 1.67 × 4.00	240 × 240
7	Philips Achieva X-series 3T	8-channel	2000	30	90	4.0/0.6	33	240	3.44 × 3.44 × 4.60	220 × 220
8	GE Excite 1.5T	16-channel	2500	35	90	4/0	36	150	4.00 × 4.00 × 4.00	256 × 256
9	Siemens Verio 3T	12-channel	2000	25	90	4/0	36	240	3.75 × 3.75 × 4.00	240 × 240
10	Siemens Tim Trio 3T	32-channel	2000	30	90	3.5/0.7	33	240	3.12 × 3.12 × 4.20	200 × 200
11	Siemens Verio 3T	12-channel	2000	25	90	4/0	36	240	3.75 × 3.75 × 4.00	240 × 240
12	Siemens Tim Trio 3T	32-channel	2500	25	90	3.5/0	39	200	3.75 × 3.75 × 3.50	240 × 240
13	Siemens Skyra 3T	32-channel	2000	30	90	3.5/0	32	240	3.50 × 3.50 × 3.50	224 × 224
14	GE Discovery MR750w 3T	24-channel	2000	30	90	3/1	35	185	3.40 × 3.40 × 4.00	220 × 220
15	Siemens Skyra 3T X	32-channel	2000	30	90	4/0	36	240	3.44 × 3.44 × 4.00	220 × 220
16	GE Discovery MR750 3T	8-channel	2000	25	90	3/1	35	200	3.75 × 3.75 × 4.00	240 × 240
17	Philips Achieva 3T	16-channel	4400	35	90	3	50	240	1.80 × 1.80 × 3.00	230 × 230
18	GE Signa 3T	8-channel	2000	30	90	5/0	30	200	3.75 × 3.75 × 5.00	240 × 240
19	Siemens Skyra 3T	32-channel	2000	30	90	4.0/0.6	33	240	3.40 × 3.40 × 4.60	220 × 220
20	GE Discovery 3T	16-channel	2000	30	90	3.2/0.4	43	200	3.40 × 3.40 × 3.40	220 × 220
21	GE Discovery MR750 3T	32-channel	2000	30	90	3.5/0	45	210	3.75 × 3.75 × 3.50	240 × 240

22	Philips Achieva 3T	8-channel	2000	30	90	4/0	38	240	3.75 × 3.75 × 4.00	240 ×240
23	GE MR750 3T	8-channel	2000	30	90	3.2/0	43	240	3.44 × 3.44 × 3.2	220 × 220

Supplementary Table 11 Image acquisition by site in the REST-meta-MDD I.

Serial Number	Scanner	Receive coil	TR (ms)	TE (ms)	Flip Angle (◦)	Thickness/gap (mm)	Slice number	Time points	Voxel size (mm)	FOV
1	Siemens Tim Trio 3T	32-channel	2000	30	90	4.0/0.8	30	210	3.28 × 3.28 × 4.80	210 × 210
2	Philips Achieva 3T	8-channel	2000	30	90	4.0/0	37	200	1.67 × 1.67 × 4.00	240 × 240
3	Siemens Magnetom Symphony scanner 1.5 T	16-channel	2000	40	90	5.0/1.25	26	150	3.75 × 3.75 × 6.25	240 × 240
4	GE Signa 3T	32-channel	3000	30	90	5.0/0	22	100	3.75 × 3.75 × 5.00	240 × 240
5	Siemens Tim Trio 3T	32-channel	2000	30	70	4/0	33	180	3.59 × 3.59 × 4.00	230 × 230
6	GE discovery MR750	8-channel	2000	30	90	3.2/0	37	184	2.29 × 2.29 × 3.20	220 × 220
7	GE Signa 3T	8-channel	2000	30	90	3.0/0	35	200	3.75 × 3.75 × 3.00	240 × 240
8	GE Discovery MR750 3.0T	8-channel	2000	25	90	3.0/1.0	35	200	3.75 × 3.75 × 4.00	240 × 240
9	Siemens Tim Trio 3T	32-channel	2000	30	90	3.0/1.52	32	212	3.75 × 3.75 × 4.52	240 × 240
10	GE Signa 3T	8-channel	2000	30	90	5	33	200	3.75 × 3.75 × 5.00	240 × 240
11	GE Signa 3T	8-channel	2000	30	90	5	33	240	3.75 × 3.75 × 4.00	240 × 240
12	GE Excite 1.5T	16-channel	2500	35	90	4/0	36	150	4.00 × 4.00 × 4.00	256 × 256
13	Siemens Tim Trio 3T	32-channel	2500	25	90	3.5/0	39	200	3.75 × 3.75 × 3.50	240 × 240
14	Siemens Verio 3.0T MRI	12-channel	2000	25	90	4/0	36	240	3.75 × 3.75 × 4.00	240 × 240
15	GE Signa 3T	8-channel	2000	30	90	5/0	30	200	3.75 × 3.75 × 5.00	240 × 240
16	GE Signa 3T	8-channel	2000	40	90	4.0/0	33	240	3.75 × 3.75 × 4.00	240 × 240
17	Philips Achieva 3.0 T scanner (Philips Healthcare, Netherlands)	8-channel SENSE head coil	2000	35	90	5.0/1.0	24	200	1.67 × 1.67 × 6.00	240 × 240
18	GE Signa 3T	8-channel	2000	22.5	30	4.0/0.6	33	240	3.44 × 3.44 × 4.60	220 × 220
19	Siemens Tim Trio 3T	12-channel	2000	30	90	3.0/1.0	32	242	3.44 × 3.44 × 4.00	220 × 220
20	Siemens Tim Trio 3T	32-channel	2000	30ms	90	3.5/0.7	33	240	3.12 × 3.12 × 4.20	200 × 200
21	Philips Gyroscan Achieva 3.0T	32-channel	2000	30	90	4.0/0	36	250	1.67 × 1.67 × 4.00	240 × 240
22	Philips Achieva 3.0T TX	8-channal	2000	30	90	4.0/0	38	240	3.75 × 3.75 × 4.00	240 × 240
23	GE Signa 1.5T	8-channel	2000	40	90	5/1	24	160	3.75 × 3.75 × 6.00	240 × 240
24	Siemens Verio 3T	12-channel	2000	25	90	4.0/0	36	240	3.75 × 3.75 × 4.00	240 × 240

Supplementary Table 12 MNI coordinates of the stimulation targets and the corresponding superficial depths for all 26 patients.

Patients	MNI coordinates			Superficial Depth (mm)
	x	y	z	
Patient 1	-27	54	24	20.49
Patient 2	-26	45	28	22.76
Patient 3	-26	48	25	24.86
Patient 4	-26	55	28	17.48
Patient 5	-31	50	33	22.34
Patient 6	-42	52	13	19.11
Patient 7	-28	48	32	21.53
Patient 8	-30	38	30	25.24
Patient 9	-27	50	23	21.15
Patient 10	-32	21	40	29.46
Patient 11	-28	52	25	20.67
Patient 12	-36	33	37	24.75
Patient 13	-29	45	32	21.92
Patient 14	-25	53	20	24.63
Patient 15	-36	44	34	17.79
Patient 16	-40	52	4	17.93
Patient 17	-30	48	35	17.91
Patient 18	-30	48	34	22.35
Patient 19	-26	48	27	23.17
Patient 20	-39	53	-1	20.38
Patient 21	-29	49	32	20.45
Patient 22	-40	53	6	25.88
Patient 23	-41	52	8	17.59
Patient 24	-40	50	1	20.75
Patient 25	-40	53	6	16.78
Patient 26	-41	53	3	17.95

Supplementary Table 13 Architecture and parameters of BrainCVAE on the REST-meta-MDD II dataset.

Layer	Output Shape	# Param	Activation	Bias	Input
**Shared encoder**					
Input Layer	(-1,1,64,64,64)	-	-	-	-
Cov3D-1	(-1,1,31,31,31)	28	ReLU	False	Input Layer
Cov3D-2	(-1,1,15,15,15)	28	ReLU	False	Cov3D-1
Flatten	(-1,3375)	0	-	False	Cov3D-2
Linear	(-1,128)	432128	ReLU	True	Flatten
S-mu	(-1,32)	4128	-	True	Linear

S-log_var	(-1,32)	4128	-	True	Linear
**Specific encoder**					
Input Layer	(-1,1,64,64,64)	-	-	-	-
Cov3D-1	(-1,1,31,31,31)	28	ReLU	False	Input Layer
Cov3D-2	(-1,1,15,15,15)	28	ReLU	False	Cov3D-1
Flatten	(-1,3375)	0	-	False	Cov3D-2
Linear	(-1,128)	432128	ReLU	True	Flatten
Z-mu	(-1,32)	4128	-	True	Linear
Z-log_var	(-1,32)	4128	-	True	Linear
**Decoder**					
Linear-1	(-1,128)	8320	LeakyReLU	True	[S_M, Z_M] or [S_H, 0]
Linear-2	(-1,3375)	435375	LeakyReLU	True	Linear-1
Reshape	(-1,1,15,15,15)	0	-	False	Linear-2
CovTranspose 3D-1	(-1,1,31,31,31)	28	LeakyReLU	False	Reshape
CovTranspose 3D-2	(-1,1,64,64,64)	28	LeakyReLU	False	CovTranspose 3D-1
Total params: 2,208,822					

Linear: Fully connected layer; Cov3D: Three-dimensional convolution layer; CovTranspose 3D: Three-dimensional transposed convolution layer; ReLU: Rectified Linear Unit; LeakyReLU: Leaky Rectified Linear Unit; S-mu/S-log_var: Fully connected layer outputting the mean / logarithm of the variance of shared features S; Z-mu/Z-log_var: Fully connected layer outputting the mean / logarithm of the variance of MDD-specific features Z.

Supplementary Table 14 Hyperparameters of BrainCVAE on the REST-meta-MDD II dataset.

Parameter	Training Value	Range	Description
Batch	15000	5000,10000,15000	Iteration steps
Learning_rate	0.005	[0.001:0.200]	Learning rate
Input_size	64, 64, 64	-	Dimension of input fALFF data
Batch_size	64	16,32,64	Batch size
Latent dimension	32	32,64,128,512	Dimension of Z/S
Kernel_size	3	[2:5]	Kernel size of cov3D && CovTranspose3D
Intermediate dimension	128	64，128，256	Dimension of the Intermediate layers in the Encoder and Decoder
negative_slope	0.45	[0.1:0.8]	Negative slope of LeakyReLU
α	1.5	[0.1:2.0]	Weight of KL loss
β	20000	[15000:25000]	Weight of disentangle loss

Supplementary Table 15 Architecture and parameters of BrainCVAE on the

REST-meta-MDD I dataset

Layer	Output Shape	# Param	Activation	Bias	Input
**Shared encoder**					
Input Layer	(-1,1,64,64,64)	-	-	-	-
Cov3D-1	(-1,1,31,31,31)	28	ReLU	False	Input Layer
Cov3D-2	(-1,1,15,15,15)	28	ReLU	False	Cov3D-1
Flatten	(-1,3375)	0	-	False	Cov3D-2
Linear	(-1,128)	432128	ReLU	True	Flatten
S-mu	(-1,32)	4128	-	True	Linear
S-log_var	(-1,32)	4128	-	True	Linear
**Specific encoder**					
Input Layer	(-1,1,64,64,64)	-	-	-	-
Cov3D-1	(-1,1,31,31,31)	28	ReLU	False	Input Layer
Cov3D-2	(-1,1,15,15,15)	28	ReLU	False	Cov3D-1
Flatten	(-1,3375)	0	-	False	Cov3D-2
Linear	(-1,128)	432128	ReLU	True	Flatten
Z-mu	(-1,32)	4128	-	True	Linear
Z-log_var	(-1,32)	4128	-	True	Linear
**Decoder**					
Linear-1	(-1,128)	8320	LeakyReLU	True	[S_M, Z_M] or [S_H, 0]
Linear-2	(-1,3375)	435375	LeakyReLU	True	Linear-1
Reshape	(-1,1,15,15,15)	0	-	False	Linear-2
CovTranspose 3D-1	(-1,1,31,31,31)	28	LeakyReLU	False	Reshape
CovTranspose 3D-2	(-1,1,64,64,64)	28	LeakyReLU	False	CovTranspose 3D-1
Total params: 2,208,822					

Linear: Fully connected layer; Cov3D: Three-dimensional convolution layer; CovTranspose 3D: Three-dimensional transposed convolution layer; ReLU: Rectified Linear Unit; LeakyReLU: Leaky Rectified Linear Unit; S-mu/S-log_var: Fully connected layer outputting the mean / logarithm of the variance of shared features S; Z-mu/Z-log_var: Fully connected layer outputting the mean / logarithm of the variance of MDD-specific features Z.

Supplementary Table 16 Hyperparameters of BrainCVAE on the REST-meta-MDD I dataset.

Parameter	Training Value	Range	Help
Batch	15000	5000,10000,15000	Iteration steps
Learning_rate	0.005	[0.001:0.200]	Learning rate
Input_size	64, 64, 64	-	Dimension of input fALFF data
Batch_size	64	16,32,64	Batch size

Latent dimension	32	32,64,128,512	Dimension of Z/S
Kernel_size	3	[2:5]	Kernel size of cov3D && CovTranspose3D
Intermediate dimension	128	64，128，256	Dimension of the Intermediate layers in the Encoder and Decoder
negative_slope	0.45	[0.1:0.8]	Negative slope of LeakyReLU
α	1.5	[0.1:2.0]	Weight of KL loss
β	20000	[15000:25000]	Weight of disentangle loss

Supplementary Table 17 Hyperparameters and range of the grid search in the inner loop of nested cross-validation.

Method	Parameter	Range	Description
MLP	learning rate	[1e-5, 1e-4, 1e-3, 1e-2,1e-1]	Step size for weight updates
	hidden layer sizes	[16, 32, 64, 128, 256, 512,1024]	Number of neurons in each hidden layer
Random Forest	n_estimators	[50, 100, 200, 500, 1000]	Number of trees
	max_depth	[5, 10, 20, 30]	Maximum tree depth
SVM	C	[0.001, 0.01, 0.1, 1, 10, 100]	Regularization parameter
	kernel	['rbf', 'linear']	Type of kernel
	gamma	['scale', 'auto', 0.001, 0.01, 0.1]	Kernel coefficient
XGBoost	n_estimators	[50, 100, 200, 500, 1000]	Number of trees
	max_depth	[3, 5, 7, 9]	Maximum tree depth
	Learning Rate	[1e-2, 5e-2, 1e-1, 5e-1]	Step size for weight updates
LightGBM	n_estimators	[50, 100, 200, 500, 1000]	Number of trees
	max_depth	[3, 5, 7, 9]	Maximum tree depth
	Learning Rate	[1e-2, 5e-2, 1e-1, 5e-1]	Step size for weight updates
Logistic Regression	C	[0.001, 0.01, 0.1, 1, 10, 100]	Regularization parameter
	solver	['liblinear', 'saga']	Optimization method
Gradient Boosting	n_estimators	[50, 100, 200, 500, 1000]	Number of trees
	max_depth	[3, 5, 7, 9]	Maximum tree depth
	learning rate	[1e-2, 5e-2, 1e-1, 5e-1]	Step size for weight updates
SVR	C	[0.001, 0.01, 0.1, 1, 10, 100]	Regularization parameter
	kernel	['rbf', 'linear']	Type of kernel
	gamma	['scale', 'auto', 0.001, 0.01, 0.1]	Kernel coefficient
Rigde	alpha	[0.0001, 0.001, 0.01, 0.1, 1, 10]	Regularization parameter
	max_iter	[500,1000, 5000, 10000]	Maximum iterations
Lasso	alpha	[0.0001, 0.001, 0.01, 0.1, 1, 10]	Regularization parameter

	max_iter	[500,1000, 5000, 10000]	Maximum iterations

Supplementary Table 18 Hyperparameters of various ML methods.

Method	Parameter
LightGBM	n_estimators = 1000, max_depth = 3, learning_rate = 5e-2
XGBoost	n_estimators = 1000, max_depth = 3, learning_rate = 5e-2
Random Forest	n_estimators = 500, max_depth = 10
Gradient Boosting	n_estimators = 500, max_depth = 3, learning_rate = 5e-2
SVR	C = 1.0, kernel = ”rbf”, epsilon = 0.1
Ridge	alpha = 1, max_iter = 1000
Lasso	alpha = 1, max_iter = 1000

Figure Legends

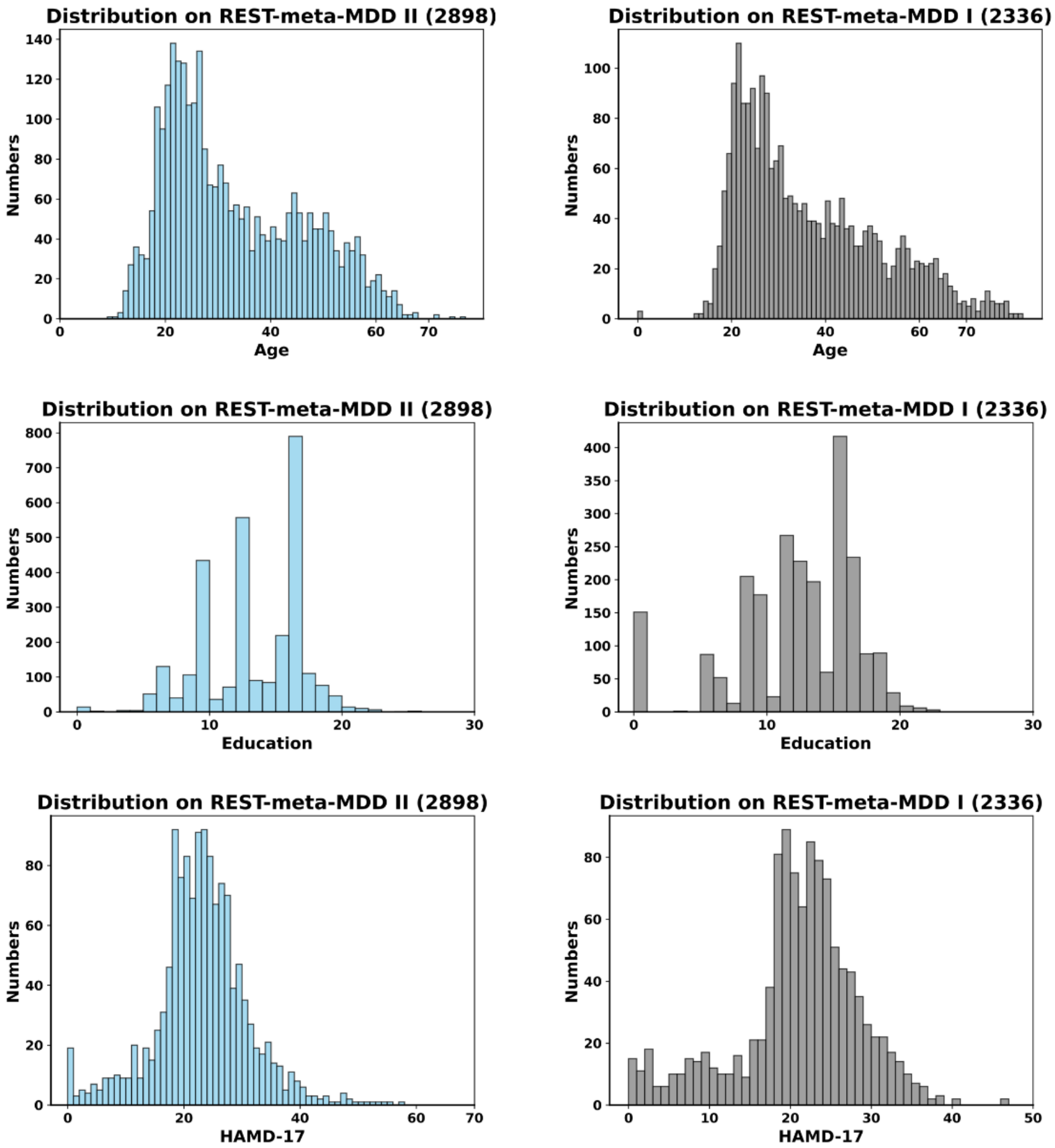

Supplementary Figure 1 Subject ages, educations and HAMD-17 scores for REST-meta-MDD II and REST-meta-MDD I, respectively.

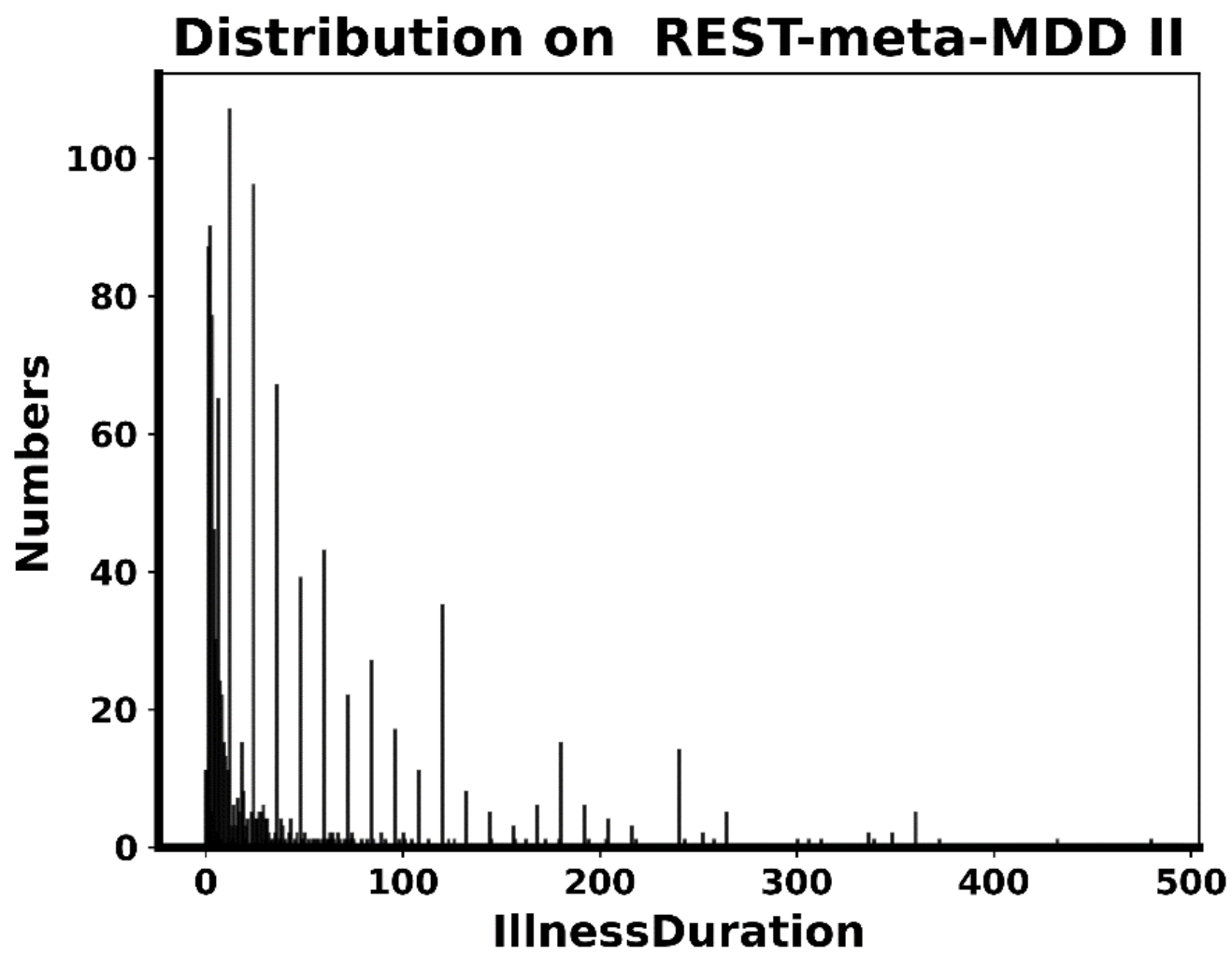

Supplementary Figure 2 Subject illness duration for REST-meta-MDD II.

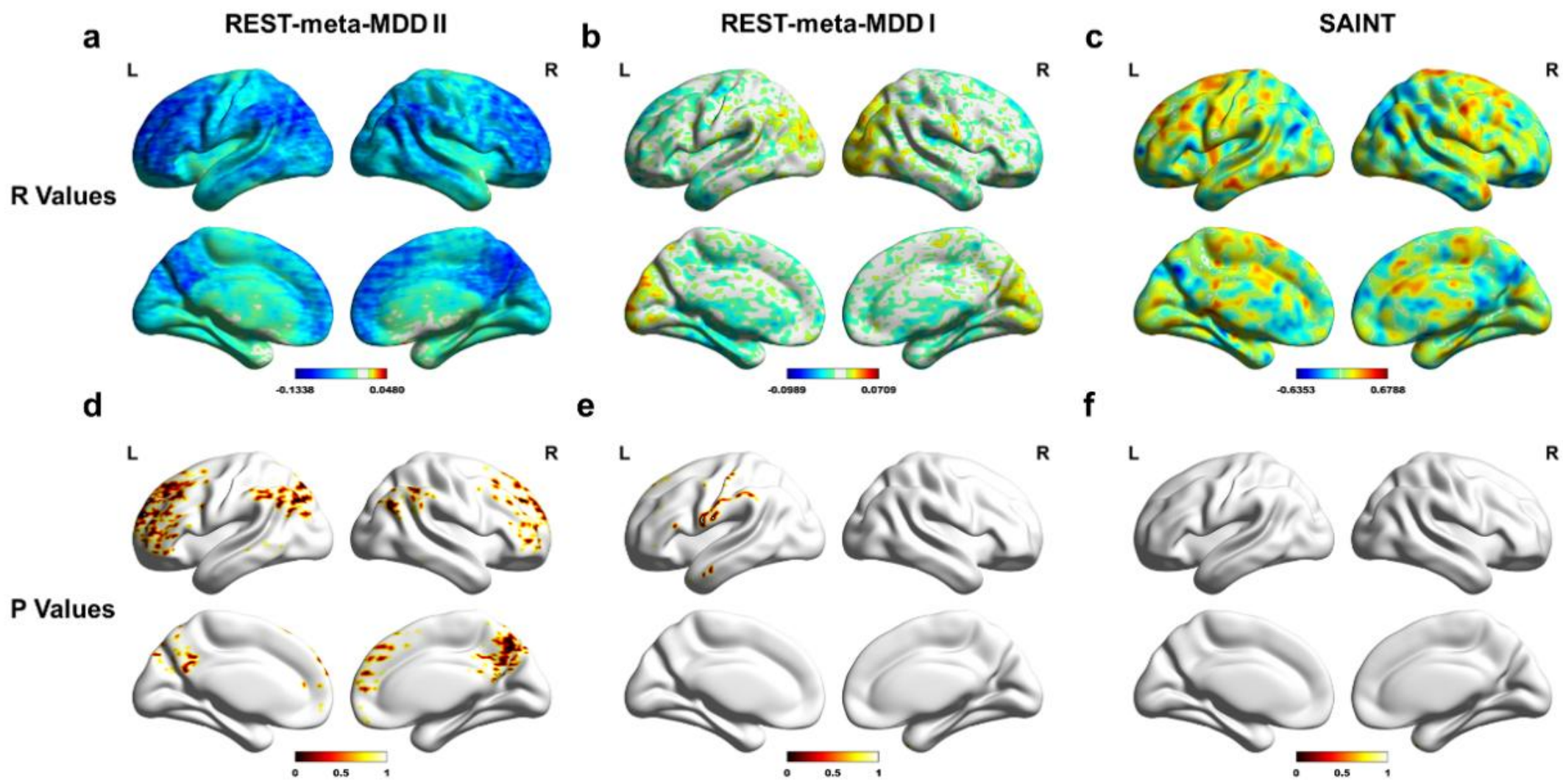

Supplementary Figure 3 Relationship between fALFF values of each voxel and mean framewise displacement (FD) across all subjects on the REST-meta-MDD II dataset (a and d), REST-meta-MDD I dataset (b and e) and SAINT dataset (c and f). a-c show Pearson correlation coefficients between fALFF values of each voxel and the mean FD across all subjects. d-e display the corresponding P values, adjusted for multiple comparisons using the Bonferroni correction, between the fALFF values of each voxel and the mean FD across all subjects. Blue indicates regions with negative correlations, while red indicates regions with positive correlations.

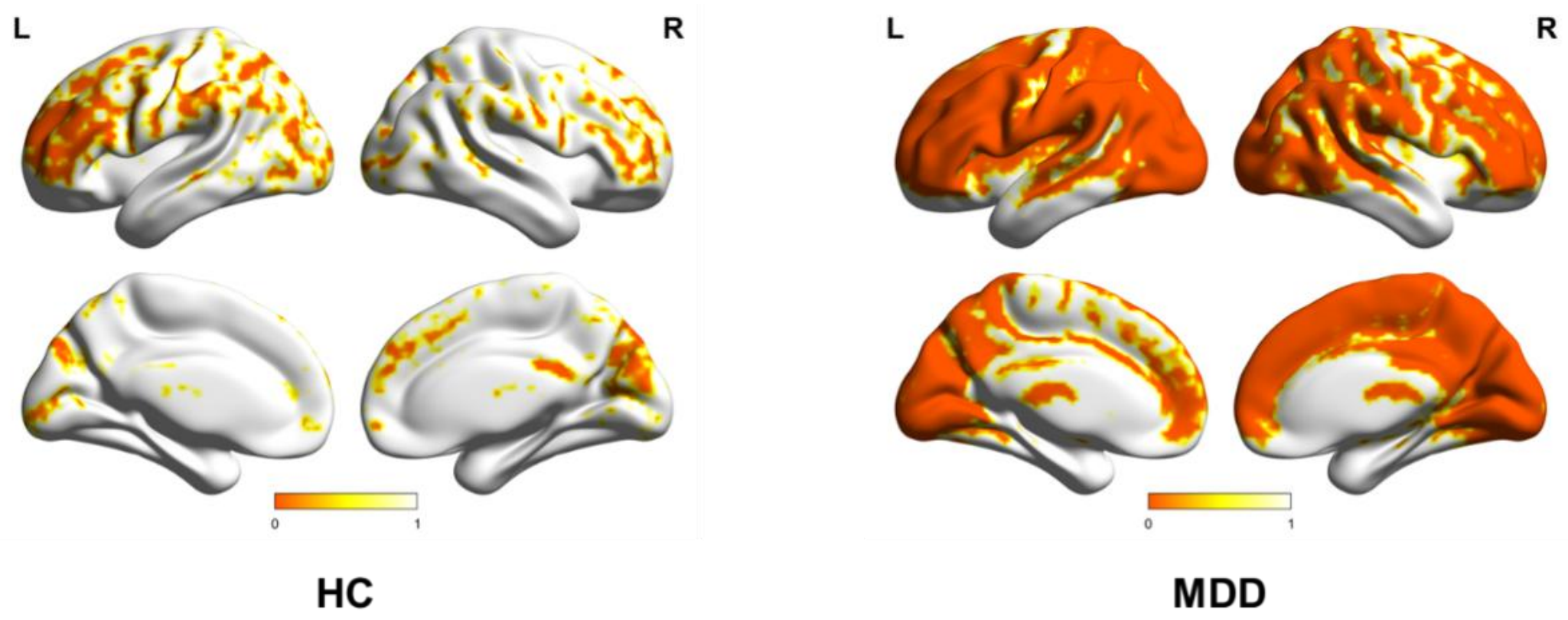

Supplementary Figure 4 Pearson correlation coefficients between fALFF values of each voxel and age across all subjects in patients with MDD and healthy controls.

Regions with deeper red indicate stronger positive correlations.

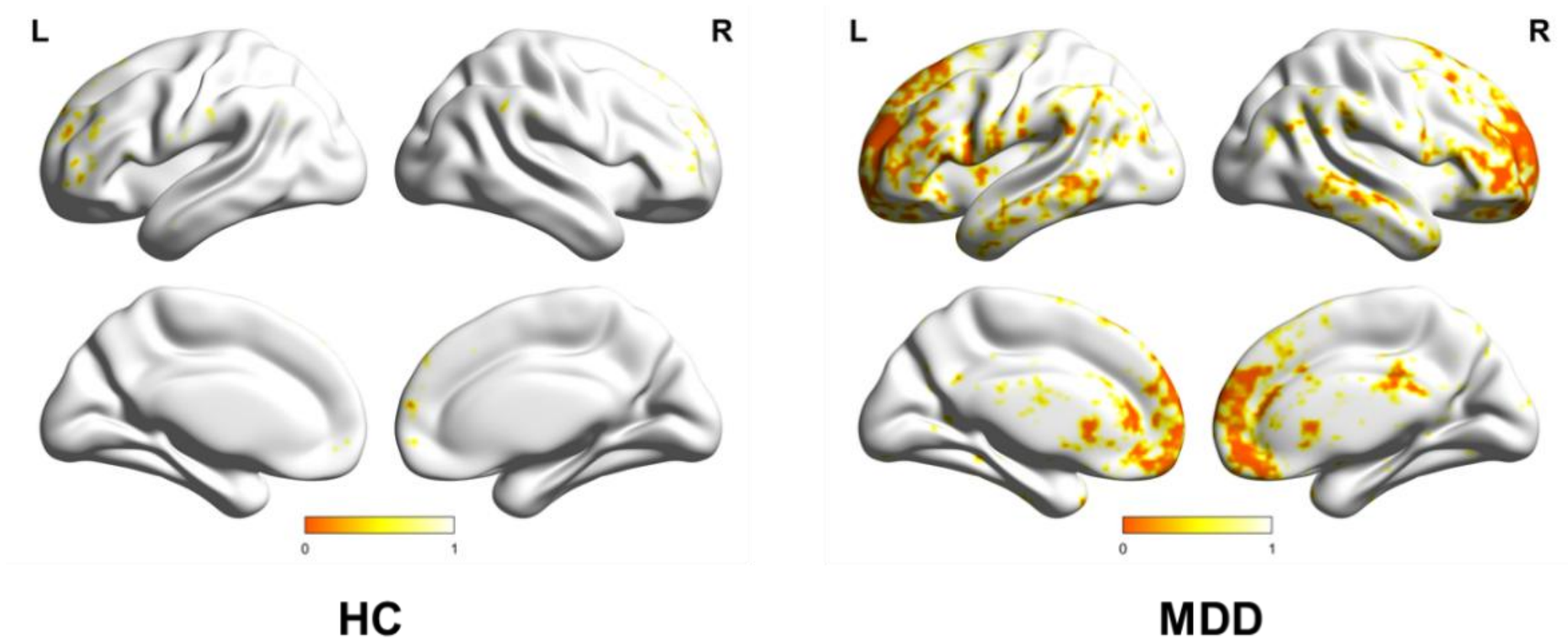

Supplementary Figure 5 Relationship between fALFF values of each voxel and education across all subjects in patients with MDD and healthy controls. Regions with deeper red indicate stronger positive correlations.

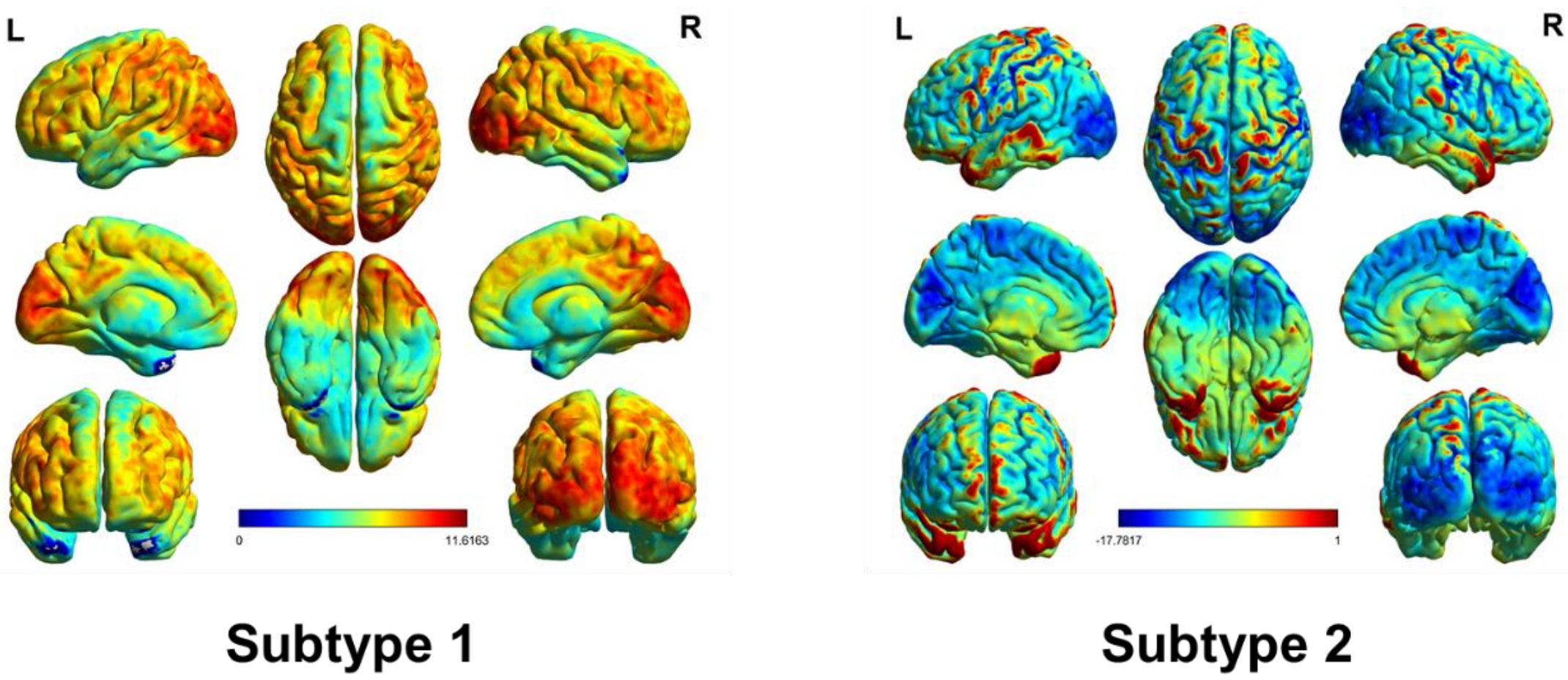

Supplementary Figure 6 Surface plot of Subtype1 and Subtype2 on the REST-meta-MDD II. The color bar illustrates the degree of group differences (t-statistic) between distinct MDD subtypes and healthy control participants.

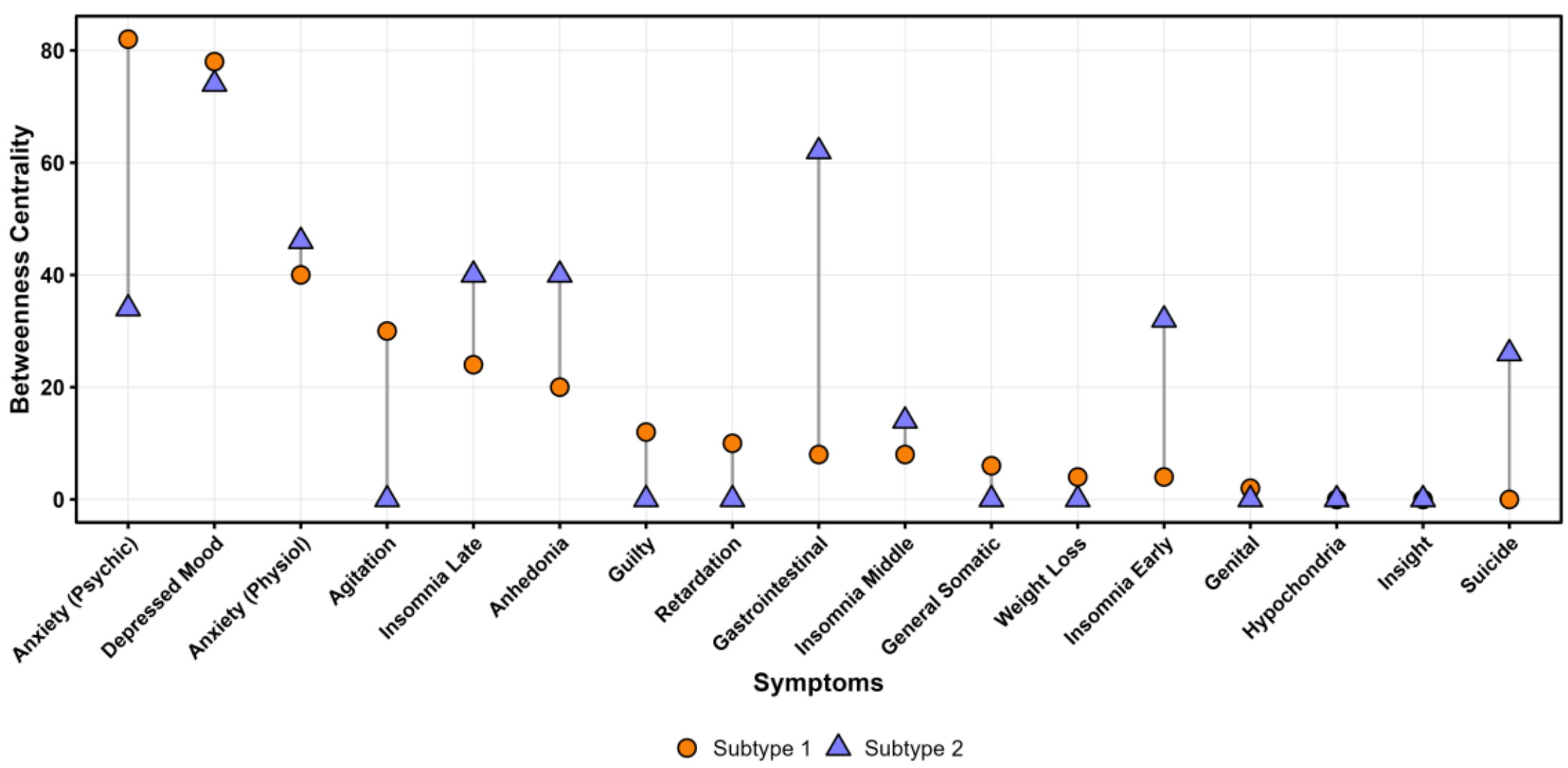

Supplementary Figure 7 Betweenness centrality of HAMD-17 symptoms across two subtypes. Betweenness centrality quantifies the frequency with which a node lies on the shortest paths between other nodes, capturing its role as a bridge within the network.

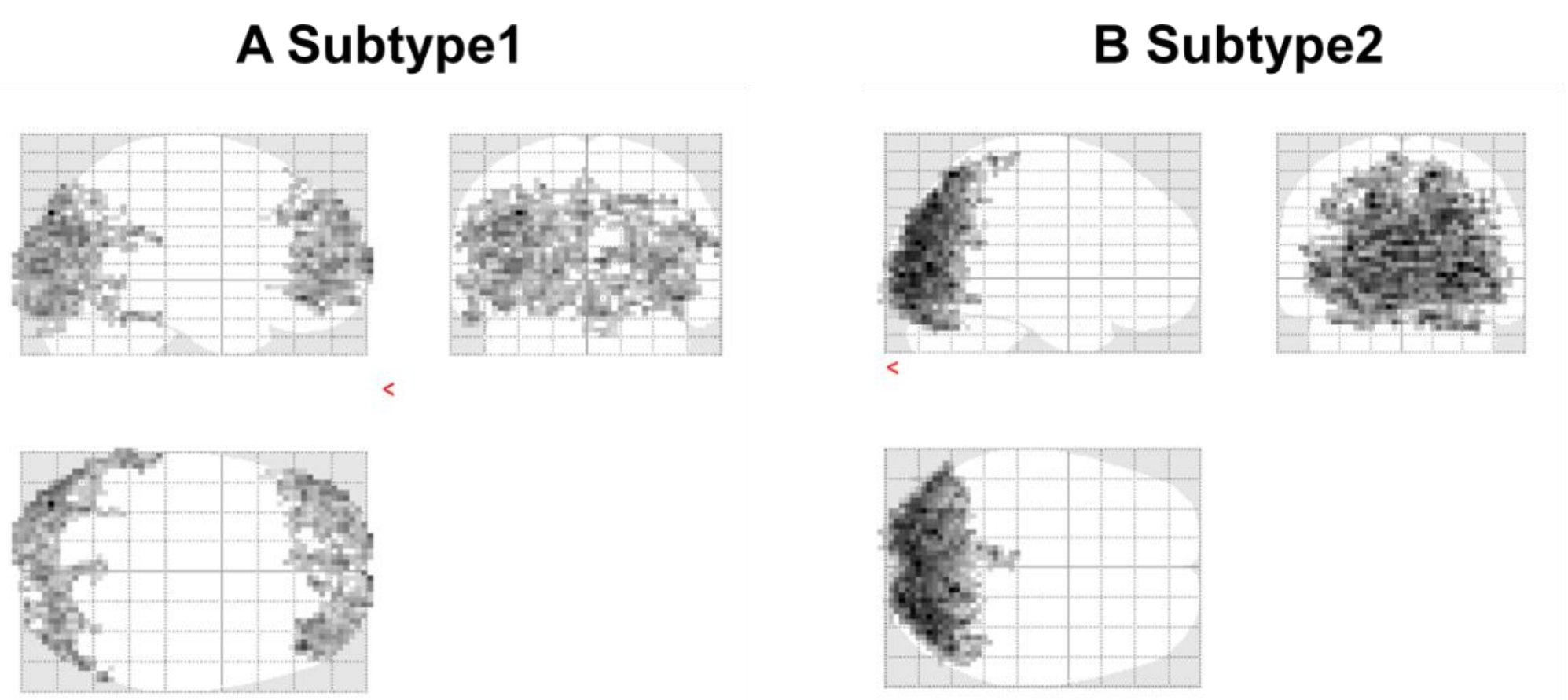

Supplementary Figure 8 Glass brain representation of subtype1 and subtype2 on the REST-meta-MDD I. Voxel-wise comparison of functional volume differences between subtype 1 (Left) and subtype 2 (Right) and controls are displayed on transverse, sagittal, and coronal sections.

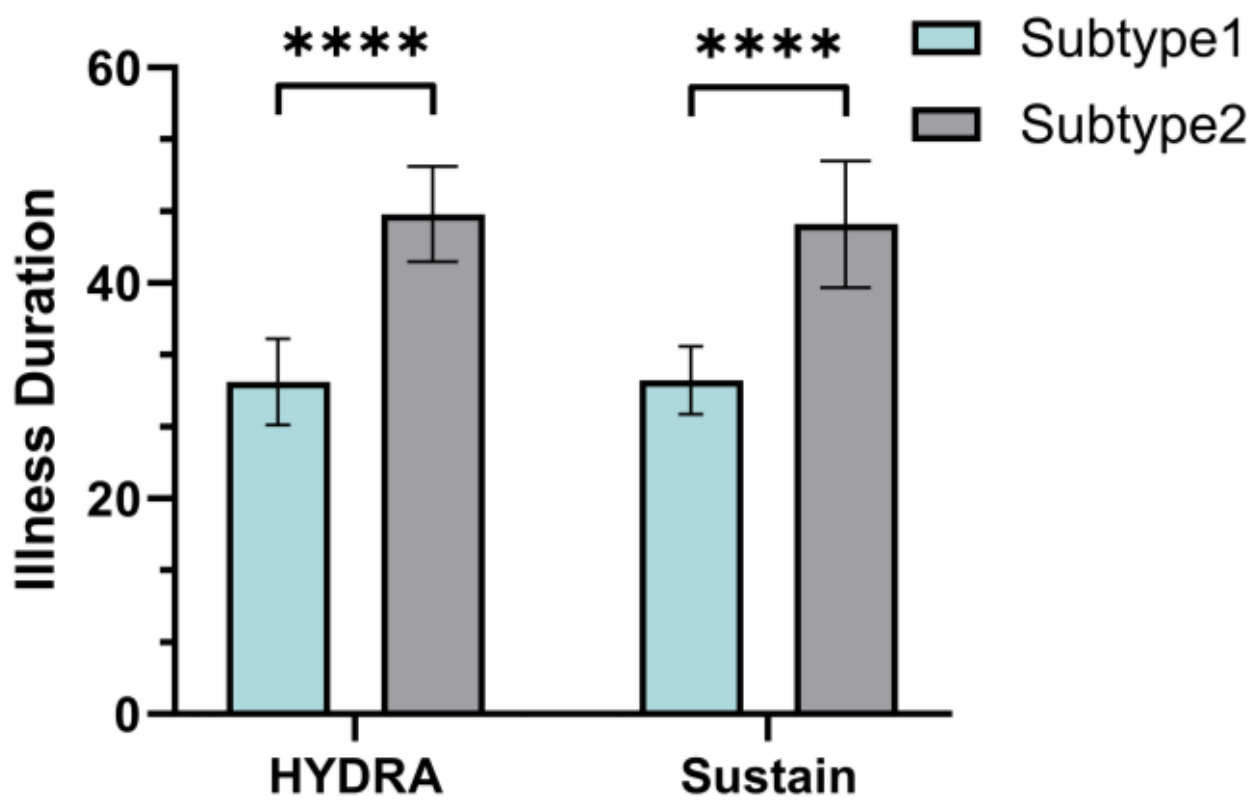

Supplementary Figure 9 Illness duration differences between subtypes identified by HYDRA and SuStaIn on the REST-meta-MDD II. ****p < 0.0001. We found that the two subtypes identified using HYDRA and SuStaIn also showed significant differences in illness duration, further supporting the robustness of our findings.

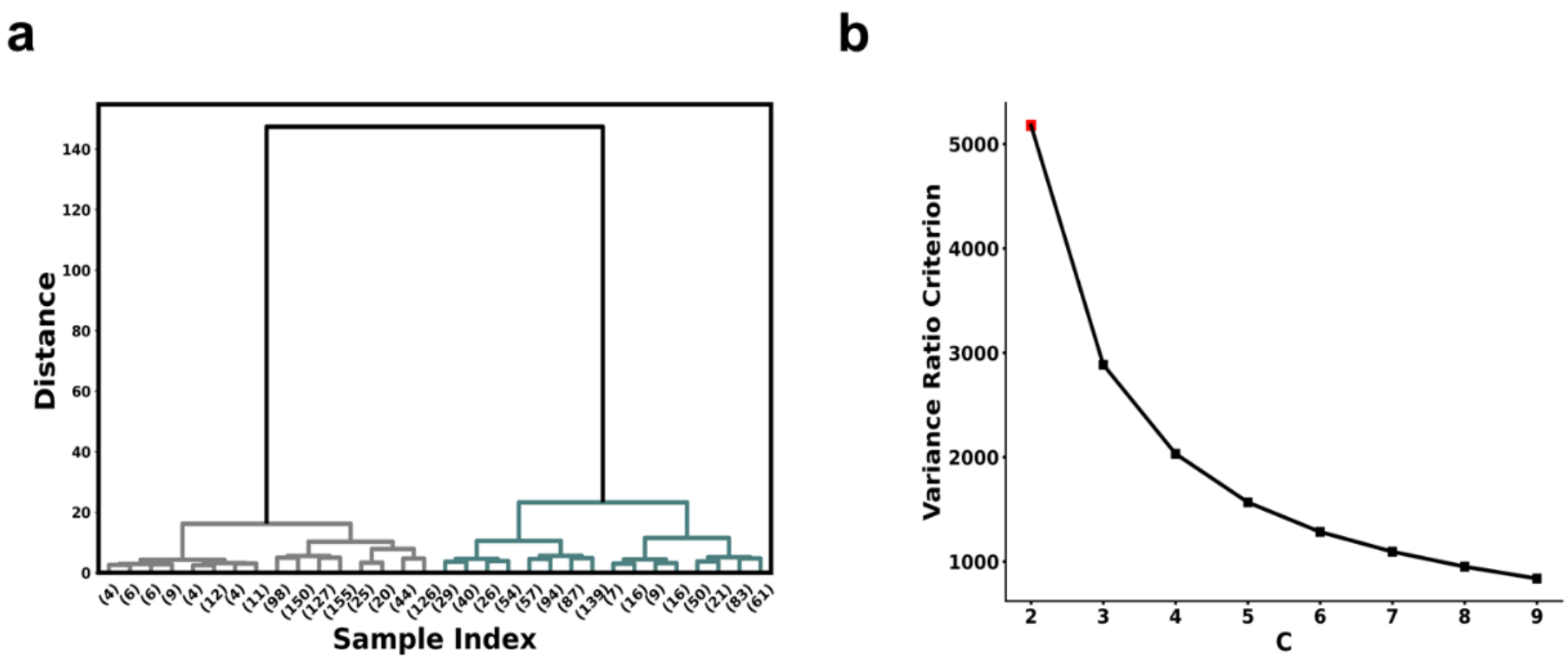

Supplementary Figure 10 Hierarchical clustering results on the REST-meta-MDD II. To assess whether our results were influenced by the clustering method, we repeated the analysis using hierarchical clustering. **a** Tree structure of hierarchical clustering. The height of each linkage in the dendrogram represents the distance between the clusters joined by that link. Note that, due to the large volume of data, only a four-level tree structure is shown. **b** Optimal number of hierarchical clustering using Variance Ratio Criterion.

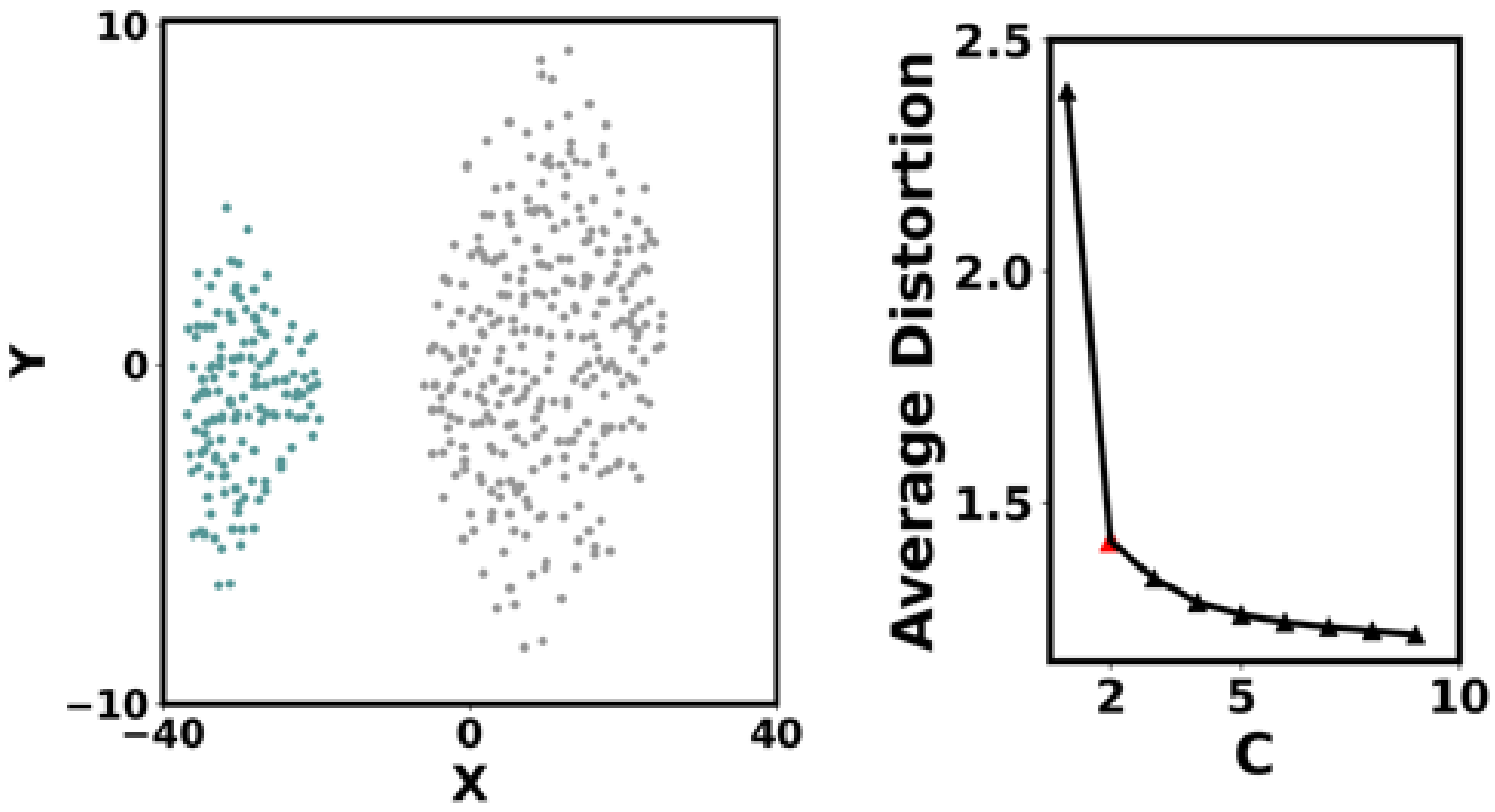

Supplementary Figure 11 Optimal clustering among patients with recurrent MDD on the REST-meta-MDD II. To examine whether illness stage may influence the subtype analysis, we restricted the analysis to patients with recurrent MDD. **a** Optimal clustering. MDD-specific features are best captured by two clusters. Scatterplot depicts individual subjects' fALFF data from subtype 1 (green) and subtype 2 (gray) groups, projected onto two-dimensional *t*-distributed stochastic neighbor embedding (*t*-SNE) dimensions computed from the MDD-specific features. **b** Clinical outcomes across the subtypes and different medication types on the patients with recurrent MDD.

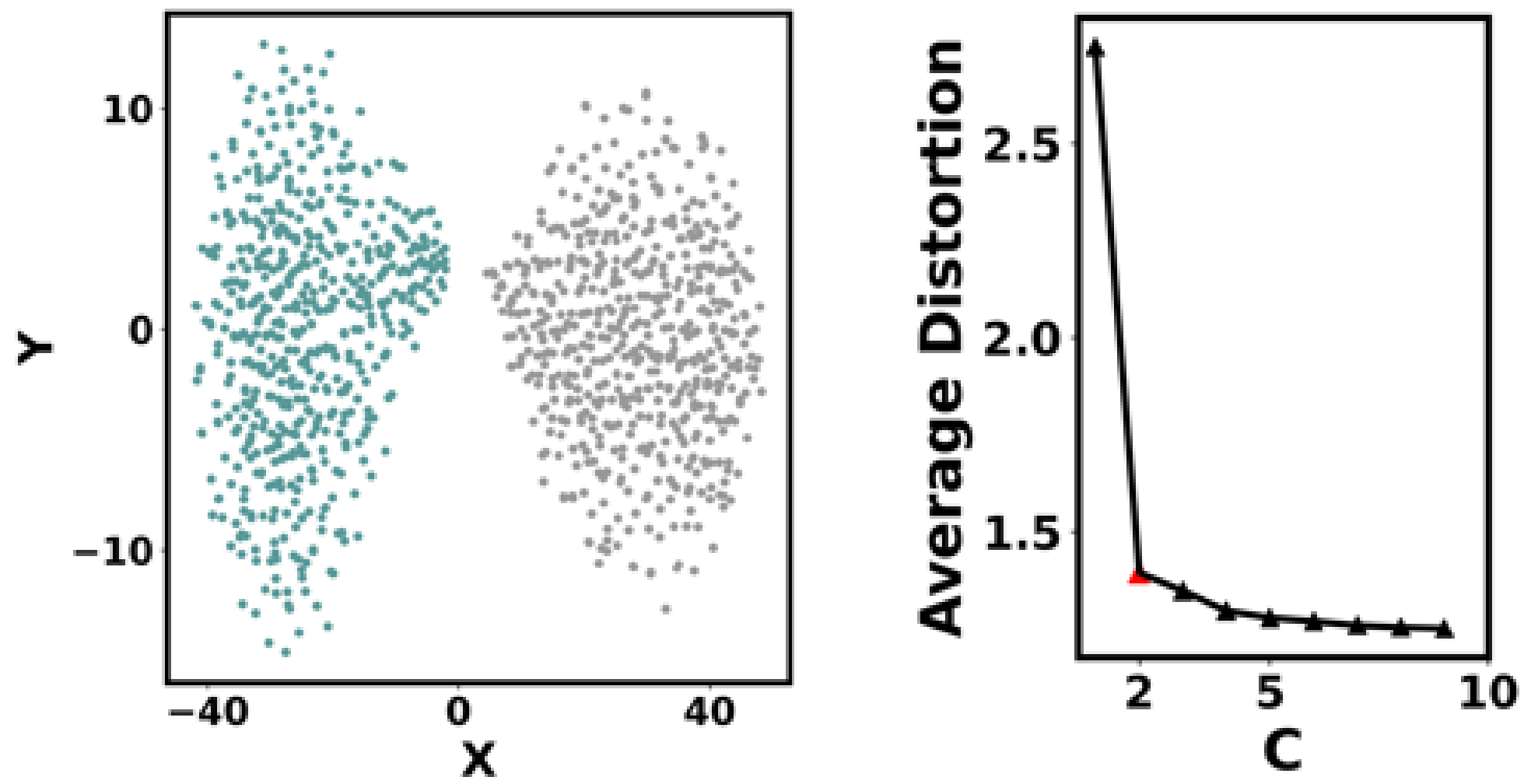

Supplementary Figure 12 Optimal clustering among patients with aged between

20 and 50 years on the REST-meta-MDD II. To assess whether age may influence subtype analysis, we excluded participants younger than 20 years or older than 50 years to repeat analysis. MDD-specific features are best captured by two clusters. Scatterplot depicts individual subjects' fALFF data from subtype 1 (green) and subtype 2 (gray) groups, projected onto two-dimensional *t*-distributed stochastic neighbor embedding (*t*-SNE) dimensions computed from the MDD-specific features.

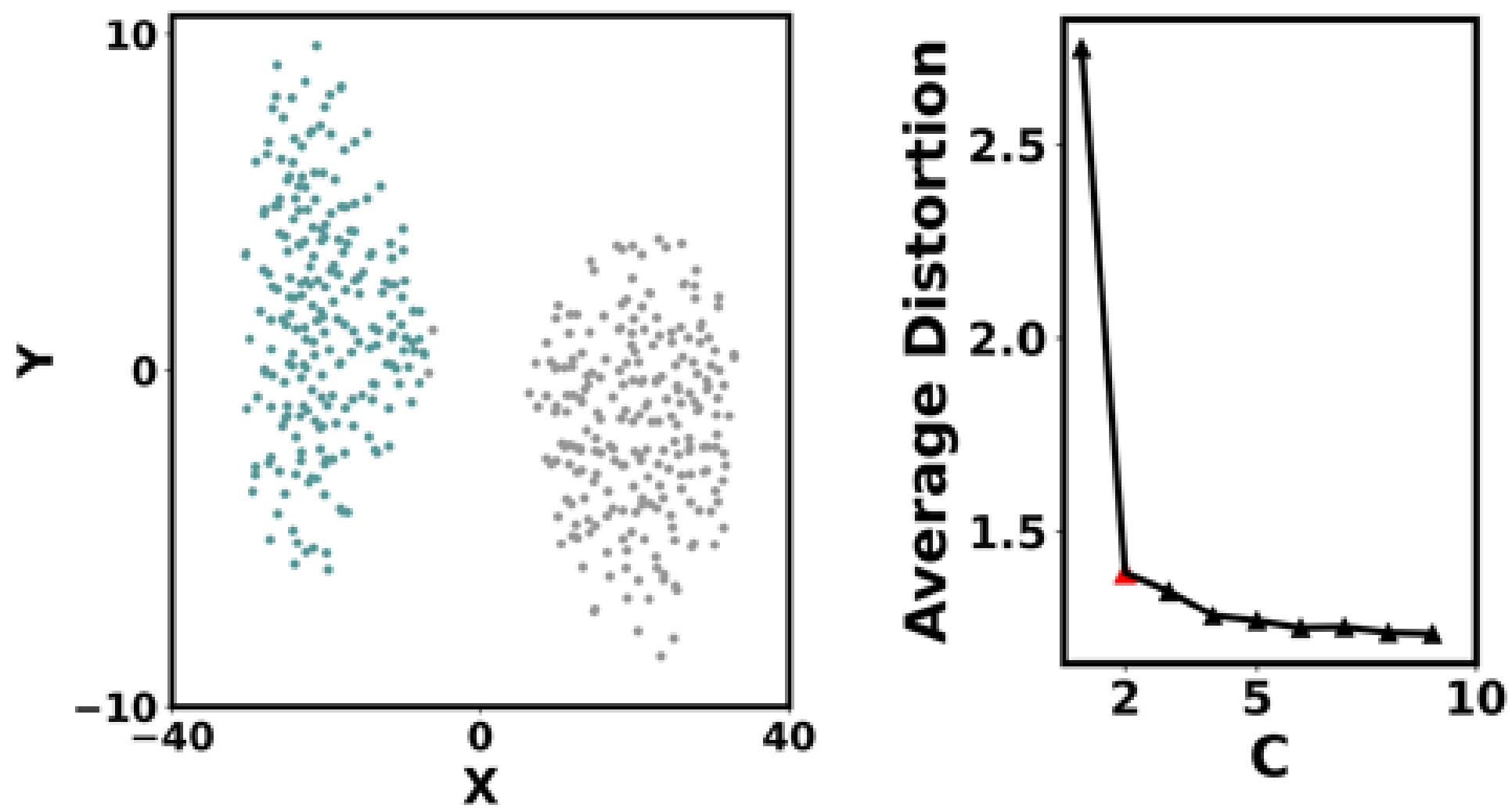

Supplementary Figure 13 Optimal clustering among patients with illness duration between 10 and 50 months on the REST-meta-MDD II. To assess whether the subtype analysis was influenced by an uneven distribution of illness duration, we excluded participants with illness duration shorter than 10 months or longer than 50 months to repeat analysis. MDD-specific features are best captured by two clusters. Scatterplot depicts individual subjects' fALFF data from subtype 1 (green) and subtype 2 (gray) groups, projected onto two-dimensional *t*-distributed stochastic neighbor embedding (*t*-SNE) dimensions computed from the MDD-specific features.

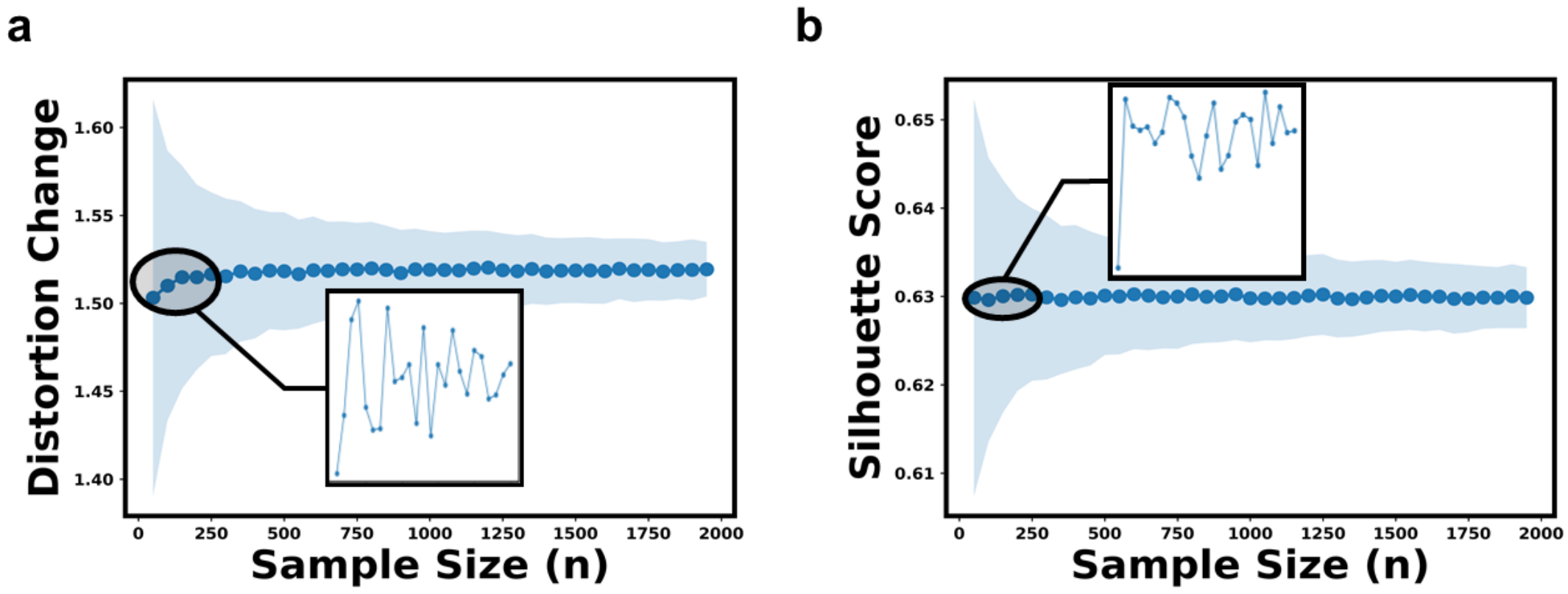

Supplementary Figure 14 Relationship between Distortion Change, the silhouette coefficient and sample size on the REST-meta-MDD II. To assess whether sample size may influence subtype analysis, we performed repeated analyses using randomly sampled subsets ranging from 50 to 2,000 participants, and evaluated the results using distortion change and the silhouette coefficient. Bootstrap analysis of (a) distortion change and (b) silhouette score across varying sample sizes. For each sample size ($n = 50 - 2{,}000$, increments of 50), 1,000 bootstrap resamples were drawn to assess clustering robustness. Blue lines represent mean values across bootstrap iterations; shaded regions show the 95% CI. Both metrics stabilize beyond $n \approx 500$.

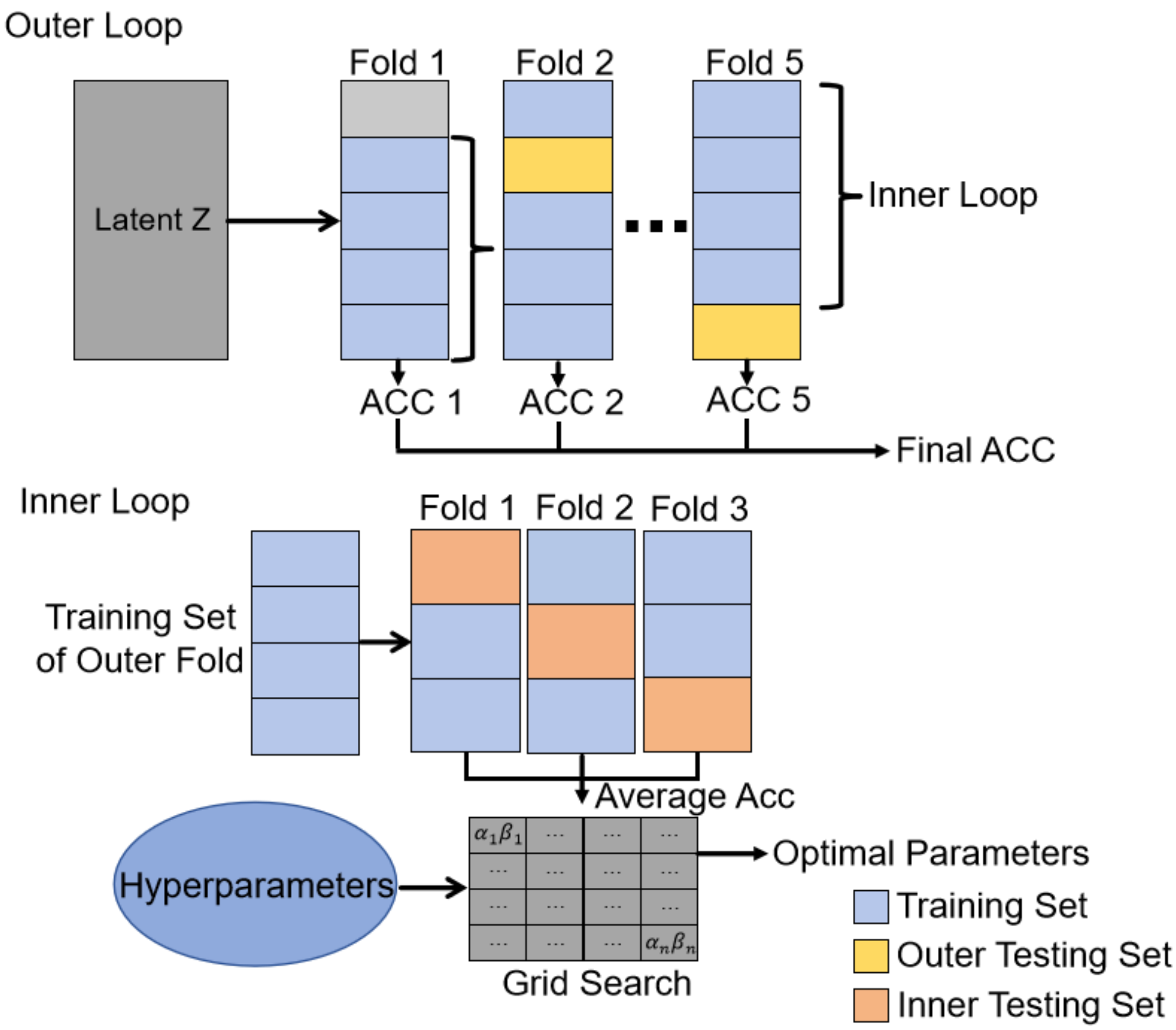

Supplementary Figure 15 Schematic of Nested Cross-Validation Process. Evaluation of both classification and regression experiments was conducted using nested cross-validation, consisting of two nested cross-validation loops. The outer loop assesses the model's generalization performance, with the dataset being split into 5 folds, where one subset is selected as the test set for each iteration to report the prediction results. The inner loop further divides the training set into 3 folds, where grid search is used to evaluate model performance across different hyperparameter combinations, outputting the optimal hyperparameter set.

Reference

1. Chuang, C.-Y., Robinson, J., Lin, Y.-C., Torralba, A. & Jegelka, S. Debiased contrastive learning. *Advances in neural information processing systems* **33**, 8765-8775 (2020).
2. Tian, Y. et al. What makes for good views for contrastive learning? *Advances in neural information processing systems* **33**, 6827-6839 (2020).
3. Aglinskas, A., Hartshorne, J.K. & Anzellotti, S. Contrastive machine learning reveals the structure of neuroanatomical variation within autism. *Science* **376**, 1070-1074 (2022).
4. Severson, K.A., Ghosh, S. & Ng, K. in Proceedings of the AAAI Conference on Artificial Intelligence, Vol. 33 4862-4869 (2019).
5. Abid, A. & Zou, J. Contrastive variational autoencoder enhances salient features. *arXiv preprint arXiv:1902.04601* (2019).
6. Chen, X. et al. The DIRECT consortium and the REST-meta-MDD project: towards neuroimaging biomarkers of major depressive disorder. *Psychoradiology* **2**, 32-42 (2022).
7. Tozzi, L. et al. Personalized brain circuit scores identify clinically distinct biotypes in depression and anxiety. *Nature medicine* **30**, 2076-2087 (2024).
8. Fu, C.H. et al. Neuroanatomical dimensions in medication-free individuals with major depressive disorder and treatment response to SSRI antidepressant medications or placebo. *Nature Mental Health*, 1-13 (2024).
9. Sun, X. et al. Mapping neurophysiological subtypes of major depressive disorder using normative models of the functional connectome. *Biological Psychiatry* **94**, 936-947 (2023).
10. Wen, J. et al. Characterizing heterogeneity in neuroimaging, cognition, clinical symptoms, and genetics among patients with late-life depression. *JAMA psychiatry* **79**, 464-474 (2022).
11. Zheng, K. et al. Redefining and subtyping of major depression based on brain functional connectivity signatures with high generalization: an ensemble hybrid framework. *bioRxiv*, 2022.2006. 2007.495073 (2022).
12. Wang, Y. et al. Data-driven clustering differentiates subtypes of major depressive disorder with distinct brain connectivity and symptom features. *The British Journal of Psychiatry* **219**, 606-613 (2021).
13. Liang, S. et al. Biotypes of major depressive disorder: Neuroimaging evidence from resting-state default mode network patterns. *NeuroImage: Clinical* **28**, 102514 (2020).
14. Drysdale, A.T. et al. Resting-state connectivity biomarkers define neurophysiological subtypes of depression. *Nature medicine* **23**, 28-38 (2017).
15. Feder, S. et al. Sample heterogeneity in unipolar depression as assessed by functional connectivity analyses is dominated by general disease effects. *Journal of affective disorders* **222**, 79-87 (2017).
16. Price, R.B. et al. Parsing heterogeneity in the brain connectivity of depressed and healthy adults during positive mood. *Biological psychiatry* **81**, 347-357 (2017).
17. Price, R.B., Gates, K., Kraynak, T.E., Thase, M.E. & Siegle, G.J. Data-driven subgroups in depression derived from directed functional connectivity paths at rest. *Neuropsychopharmacology* **42**, 2623-2632 (2017).
18. Cheng, Y. et al. Delineation of early and later adult onset depression by diffusion tensor imaging. *PloS one* **9**, e112307 (2014).

CONSORT checklist

Section/Topic	Item No	Checklist item	Reported on page No
**Title and abstract**			
	1a	Identification as a randomised trial in the title	P.1
	1b	Structured summary of trial design, methods, results, and conclusions (for specific guidance see CONSORT for abstracts)	NA
**Introduction**			
Background and objectives	2a	Scientific background and explanation of rationale	P.4-P.6
	2b	Specific objectives or hypotheses	P.6-P.7
**Methods**			
Trial design	3a	Description of trial design (such as parallel, factorial) including allocation ratio	NA
	3b	Important changes to methods after trial commencement (such as eligibility criteria), with reasons	NA
Participants	4a	Eligibility criteria for participants	P.30-P.31
	4b	Settings and locations where the data were collected	P.30-P.31
Interventions	5	The interventions for each group with sufficient details to allow replication, including how and when they were actually administered	NA
Outcomes	6a	Completely defined pre-specified primary and secondary outcome measures, including how and when they were assessed	P.32-P.42
	6b	Any changes to trial outcomes after the trial commenced, with reasons	P.32-P.42
Sample size	7a	How sample size was determined	P.30-P.31
	7b	When applicable, explanation of any interim analyses and stopping guidelines	P.30-P.31
Randomisation:			
Sequence generation	8a	Method used to generate the random allocation sequence	NA
	8b	Type of randomisation; details of any restriction (such as blocking and block size)	NA

Allocation concealment mechanism	9	Mechanism used to implement the random allocation sequence (such as sequentially numbered containers), describing any steps taken to conceal the sequence until interventions were assigned	NA
Implementation	10	Who generated the random allocation sequence, who enrolled participants, and who assigned participants to interventions	P.30-P.31
Blinding	11a	If done, who was blinded after assignment to interventions (for example, participants, care providers, those assessing outcomes) and how	P.32-P.42
	11b	If relevant, description of the similarity of interventions	NA
Statistical methods	12a	Statistical methods used to compare groups for primary and secondary outcomes	P.41-P.42
	12b	Methods for additional analyses, such as subgroup analyses and adjusted analyses	P.41-P.42
**Results**			
Participant flow (a diagram is strongly recommended)	13a	For each group, the numbers of participants who were randomly assigned, received intended treatment, and were analysed for the primary outcome	P.7
	13b	For each group, losses and exclusions after randomisation, together with reasons	P.7
Recruitment	14a	Dates defining the periods of recruitment and follow-up	P.30-P.31
	14b	Why the trial ended or was stopped	P.30-P31
Baseline data	15	A table showing baseline demographic and clinical characteristics for each group	P.58-P.59
Numbers analysed	16	For each group, number of participants (denominator) included in each analysis and whether the analysis was by original assigned groups	P.58-P.59
Outcomes and estimation	17a	For each primary and secondary outcome, results for each group, and the estimated effect size and its precision (such as 95% confidence interval)	P.7-P.20
	17b	For binary outcomes, presentation of both absolute and relative effect sizes is recommended	P.7-P.20

Ancillary analyses	18	Results of any other analyses performed, including subgroup analyses and adjusted analyses, distinguishing pre-specified from exploratory	P.7-P.20
Harms	19	All important harms or unintended effects in each group (for specific guidance see CONSORT for harms)	NA
**Discussion**			
Limitations	20	Trial limitations, addressing sources of potential bias, imprecision, and, if relevant, multiplicity of analyses	P.28- P.29
Generalisability	21	Generalisability (external validity, applicability) of the trial findings	P.9- P.10
Interpretation	22	Interpretation consistent with results, balancing benefits and harms, and considering other relevant evidence	P.7-P.20
**Other information**			
Registration	23	Registration number and name of trial registry	P.3
Protocol	24	Where the full trial protocol can be accessed, if available	P.11-P.17
Funding	25	Sources of funding and other support (such as supply of drugs), role of funders	P.28
